# Mechanical scission of a knotted polymer

**DOI:** 10.1038/s41557-024-01510-3

**Published:** 2024-04-22

**Authors:** Min Zhang, Robert Nixon, Fredrik Schaufelberger, Lucian Pirvu, Guillaume De Bo, David A. Leigh

**Affiliations:** 1 School of Chemistry and Molecular Engineering, East China Normal University, Shanghai, China; 2 Department of Chemistry, University of Manchester, Manchester, UK

**Keywords:** Interlocked molecules, Polymer chemistry

## Abstract

Molecular knots and entanglements form randomly and spontaneously in both biological and synthetic polymer chains. It is known that macroscopic materials, such as ropes, are substantially weakened by the presence of knots, but until now it has been unclear whether similar behaviour occurs on a molecular level. Here we show that the presence of a well-defined overhand knot in a polymer chain substantially increases the rate of scission of the polymer under tension (≥2.6× faster) in solution, because deformation of the polymer backbone induced by the tightening knot activates otherwise unreactive covalent bonds. The fragments formed upon severing of the knotted chain differ from those that arise from cleavage of a similar, but unknotted, polymer. Our solution studies provide experimental evidence that knotting can contribute to higher mechanical scission rates of polymers. It also demonstrates that entanglement design can be used to generate mechanophores that are among the most reactive described to date, providing opportunities to increase the reactivity of otherwise inert functional groups.

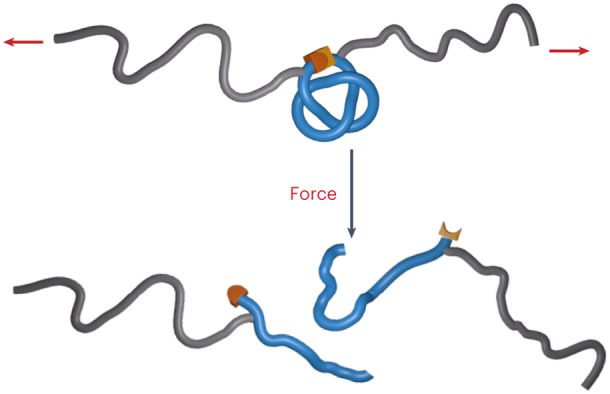

## Abstract (web summary)

Knots reduce the tensile strength of macroscopic threads and fibres. Now it has been shown that the presence of a well-defined overhand knot in a polymer chain can substantially increase the rate of scission of the polymer under tension, as deformation of the polymer backbone induced by the tightening knot activates otherwise unreactive covalent bonds.

## Main

Upon tightening, knots weaken macroscopic strands until they fracture at the entrance to the entanglement^1,2^. This reduces the tensile strength of knotted materials from ropes used in sailing and mountaineering to fishing lines. Computer simulations^3^, theory^4,5^ and intuition^5^ suggest that similar weakening may occur at the nanoscale, but such processes have not previously been explored experimentally. The effects of mechanical pulling^6–9^ on the conformation of molecular knots^10–13^ have been probed by force microscopies, but the smallest-scale knot-breaking experiments to date were carried out on actin filaments 0.4–0.8 μm in diameter using optical tweezers^14^. In polymer mechanochemistry, polymers are used to stretch mechanosensitive molecular structures (mechanophores) at the nanoscale^15^. This can be achieved in solution using ultrasound-induced cavitation because of the elongational flow generated in the vicinity of collapsing bubbles^16^. This technique has been used to investigate the effect of interlocking components on the mechanical strength of covalent bonds^17^ in catenanes^18,19^ and rotaxanes^20–23^ (which respectively reduce or enhance tensional stress). Recent advances in molecular knot synthesis^24–34^ mean that small-molecule knots are now accessible structural motifs that can be integrated into more complex molecular systems^35–37^.

**Fig. 1:**
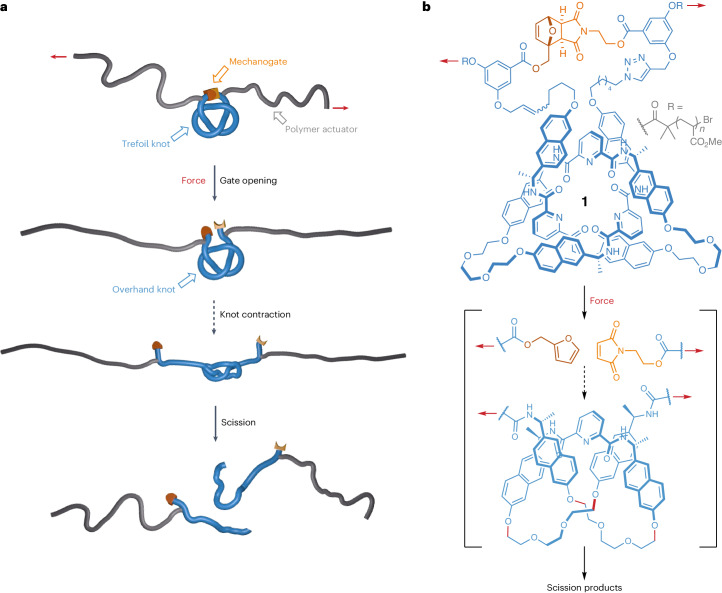
Mechanical scission of an overhand knot in a polymer by cavitation-induced elongational flow. **a**, Cavitation-induced elongational flow is used to stretch a gated overhand knot (a trefoil knot) derivatized with actuating polymer chains. **b**, Scission of the Diels–Alder gate in trefoil knot **1** reveals a transient overhand knot that contracts and eventually breaks upon continuous elongation. Red arrows indicate the direction of the force. Plain and dashed reaction arrows indicate covalent and non-covalent processes, respectively. Potential scissile bonds of the knot are shown in red.

We decided to incorporate a knotted molecular building block into a polymer to investigate the effect of a structurally well-defined overhand (trefoil, 3_1_) knot, the simplest and most abundant knot formed spontaneously in linear polymer chains^13,38^, on the rate of scission of a polymer chain under tension (Fig. 1a), and to determine which bonds were broken. The study was carried out using sonication on polymers in solution, but the order of scission rates of functional groups and other structural elements in solution mechanochemistry generally correlates with the order of their breaking rates in the condensed phase^39^.

The central region of a polymer experiences the largest forces during sonication^17^, making it the most suitable location to incorporate a knot for these experiments. However, open knots, such as the overhand trefoil knot, are dynamic structures that can expand and contract or translocate along the polymer backbone^13,40,41^. To avoid unknotting occurring before the polymer chain was under tension, we gated^42^ the overhand knot with a mechanically labile unit (a mechanophore built around a furan/maleimide Diels–Alder adduct) that would maintain the integrity of the knotted architecture in the form of a closed-loop trefoil knot (Fig. 1)^43^. Upon stretching, tension builds up along the polymer backbone, ultimately reaching the knot. Because the gate mechanophore is situated along the shortest path connecting the two sides of the polymer, it will be activated before covalent bonds in other regions of the knot^19^. Dissociation of the gate converts the closed-loop knot into an open overhand knot and enables the polymer to further stretch and tighten the knot until the eventual rupture of a covalent bond occurs, breaking the polymer chain (Fig. 1a). This outcome can only be realized if this sequence takes place during the same elongation event (that is, the overhand knot does not have time to unravel).

## Results and discussion

The gated overhand knot (the closed-loop trefoil knot) was assembled from a lanthanide-complexed overhand knot^26^, a versatile motif that has previously been used for the assembly of complex knots^31,34^ and investigation of their properties^44,45^. The overhand knot was macrocyclized to form the trefoil knot by connection to the gate through successive Cu-catalysed azide–alkyne cycloaddition and ring-closing olefin metathesis reactions (Supplementary Section 3.4). The chain-centred knot (**1**, *M*_n_ (number average molecular weight) = 62 kDa, *Đ* (dispersity) = 1.27) was obtained by single electron transfer living radical polymerization^46^ of methyl acrylate initiated from both sides of the gating unit (Fig. 1b).

### Sonication kinetics

**Fig. 2:**
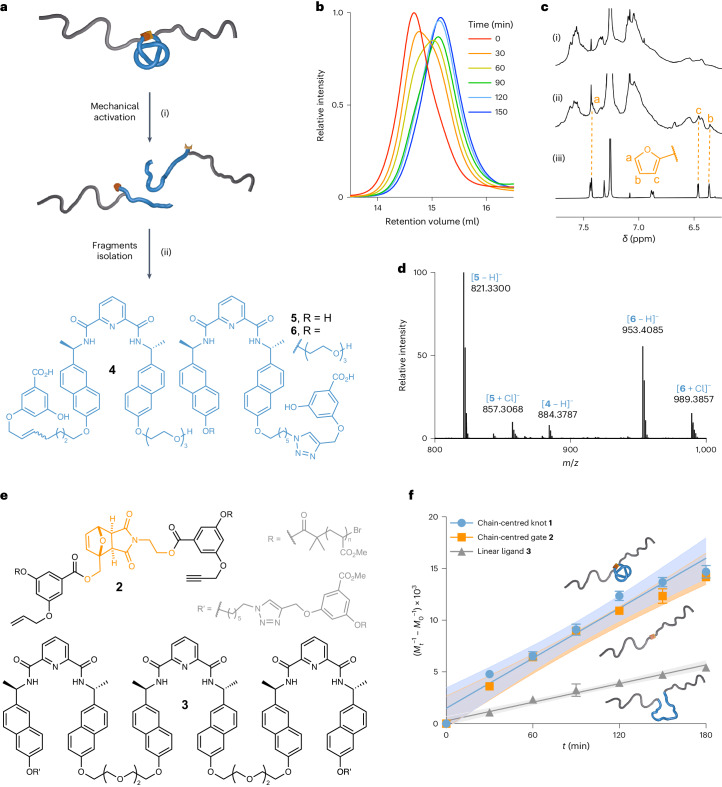
Mechanical activation of chain-centred knot 1, gate 2 and linear ligand 3. **a**, Mechanical activation of chain-centred knot **1** and isolation of the resulting fragments. Conditions: (i) ultrasound (20 kHz, 11.5 W cm^−^^2^, 1 s on/2 s off), CH_3_CN, 5–10 °C, 180 min; (ii) NaOH. **b**, The overlay of gel-permeation chromatography traces at various sonication times of chain-centred knot **1** (tetrahydrofuran, 1 ml min^−1^) is consistent with a rupture in the central region of the polymer. **c**, Partial ^1^H NMR (500 MHz, CDCl_3_) spectra of knot **1** before (i) and after (ii) sonication, along with a reference compound (iii), indicate opening of the gate adduct through mechanical activation. **d**, Mass spectrometry (electrospray ionisation high-resolution mass spectrometry, negative ion mode) identification of fragments **4**, **5** and **6** in the hydrolysed post-sonication mixture. **e**, Structure of chain-centred gate (**2**) and linear ligand (**3**). **f**, Dissociation kinetics of chain-centred knot (**1**), gate (**2**) and linear ligand (**3**). *M*_*t*_ = *M*_n_ at time *t*, *M*_0_ = *M*_n_ at 0 min. Solid lines correspond to a linear fit; *R*^2^ (goodness of fit) = 0.963, 0.976 and 0.988 for **1**, **2** and **3**, respectively. Each point corresponds to the average of three sonication experiments. Data are presented as means ± s.e.m. Coloured areas indicate 95% confidence levels. Source data

To assess the effect of the knot on the mechanical strength of a polymer chain, we compared the rate of dissociation upon mechanical activation of chain-centred knot **1**, gate unit **2** (*M*_n_ = 71 kDa, *Đ* = 1.23) and (unknotted) linear ligand **3** (*M*_n_ = 67 kDa, *Đ* = 1.13) (Fig. 2a–c). Mechanical activation was performed in acetonitrile at 5–10 °C, using high-intensity ultrasound (20 kHz, 11.5 W cm^−^^2^, 1 s on/2 s off, 180 min). The progress of the reaction was monitored by gel-permeation chromatography (Fig. 2b) and the conversion plotted using the Nalepa method^47^ (Fig. 2f). The apparent dissociation rate (k*) is obtained from the slope of the traces in Fig. 2f. Comparison of the reaction rates (k* = 8.1 ± 0.7, 7.7 ± 0.5 and 3.0 ± 0.1 min^−1^ kDa^−1^ 10^5^ for **1**, **2** and **3**, respectively) show that knot **1** and gate **2** cleave at similar rates, whereas the dissociation of the polymer containing the linear ligand (**3**) is substantially slower. These results show that the structure of the linear ligand used to assemble the knot is mechanically stronger than gate **2**. Indeed, **2** can readily cleave via a retro-cycloaddition pathway to restore the furan and maleimide rings composing the initial Diels–Alder adduct, whereas the ligand segment in **3** does not contain any obviously weak bonds and cleaves in the poly(methyl acrylate) backbone (Supplementary Section 4.3). We found that once embedded in knot **1**, the same segment cleaves substantially faster. In other words, the knot architecture accelerates the dissociation of this segment, and the cleavage of the gating mechanophore is the rate-determining step of the dissociation process. This also indicates that in knot **1**, both the gate and the resulting overhand knot must cleave in the same elongation event because the intermediate overhand knot (Fig. 1) would unravel under force-free conditions and the resulting linear ligand would then cleave at a slower rate.

### Fragments analysis

Because the knotted architecture of **1** clearly enhances the mechanochemical reactivity of its constituent covalent bonds (the unknotted polymer, **3**, is much more slowly cleaved), we then sought to determine the scission point(s) to establish the origin of the enhanced reactivity. The scission of the Diels–Alder gate was confirmed by ^1^H NMR spectroscopy (Fig. 2c), but the structural complexity of the various components (knot, polymer, mechanophore, fragments and so on) prevented further insights from NMR. Hence, we attempted to isolate the fragments of the broken knot, because hydrolysis of the post-sonication mixture should lead to removal of the polymer arms and gate elements. The post-sonication mixture was hydrolysed with NaOH and then adjusted to pH 3 and the resulting solution extracted with CH_2_Cl_2_. Three major species (**4**, **5** and **6**) (Fig. 2a) were identified by high-resolution mass spectrometry from the CH_2_Cl_2_ fraction (Fig. 2d). Their structures are indicative of mechanical scission of the C–O bond adjacent to one of the naphthyl groups (Fig. 1b), resulting in naphthol (**5**) and ethylene glycol (**4**, **6**) fragments (Fig. 2a). We were able to isolate fragments **4** and **6** and confirm their identity by ^1^H NMR spectroscopy and high-resolution mass spectrometry (Supplementary Section 4.9). The fact that only the naphthyl ethers connected to the ethylene glycol linkers are observed to cleave suggests that the knot contracts mainly around the central pyridyl unit (Fig. 1b). We did not isolate or detect any scission products that would occur from migration of the knot along the strand while it is under tension during the elongation event, although we cannot rule out that occurring to a minor extent.

### Calculations

**Fig. 3:**
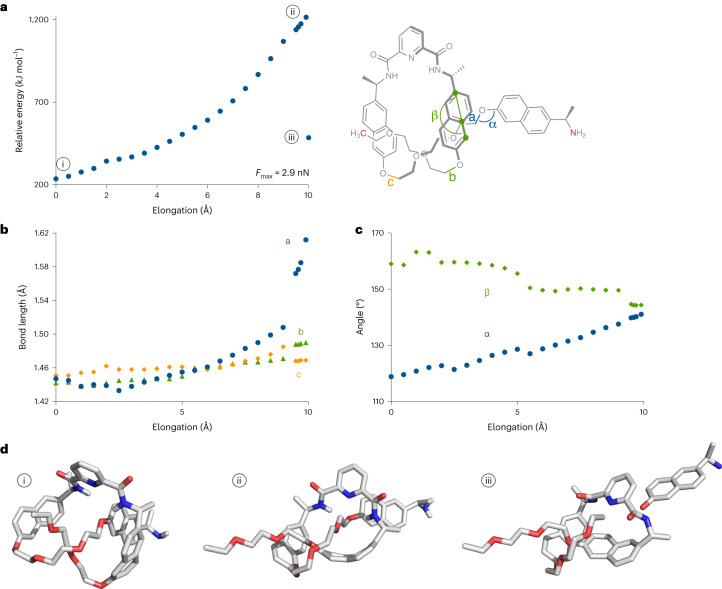
Computational analysis of knot scission. **a**, CoGEF simulation (UB3LYP/6-31G*, gas phase) on a model knot; anchor atoms are shown in red. **b**, Tensile deformation of scissile (labelled a) and non-scissile (labelled b and c) C–O_napht_ bonds. **c**, Angular deformation around the scissile bond (α) and a naphthyl group (β). **d**, CoGEF structures at onset (i), maximal deformation (ii) and after scission (iii) correspond to the states highlighted in **a**. Source data

Constrained geometries simulate external force (CoGEF)^48^ calculations gave further insight into the scission process. The extension of the knot was first simulated by molecular mechanics (Merck molecular force field (MMFF)) on a model knot lacking the gate unit (Supplementary Section 9). The actual scission of the knot was simulated on a shorter model extracted from a contracted intermediate of the MMFF profile. More specifically, the intermediate stretched by 40 Å was isolated and excised of the unknotted sections of the ligand (caused by contraction of the knot; Supplementary Section 9). The elongation of this shorter model was then simulated by density functional theory (UB3LYP/6-31G*, gas phase) until bond scission occurred (Fig. 3a). Although the CoGEF method does not allow for a detailed mechanistic interpretation of the dissociation process (because it does not account for dynamic or thermal effects), it has proved successful in mapping^19,21,22^ conformation and bond deformation, as well as in identifying the scissile bond(s) in mechanophores^49^. The CoGEF profile displays a steady increase in energy from the starting structure (Fig. 3a(i)) to the state of maximal deformation (Fig. 3a(ii)), which is followed by a heterolytic bond scission (Fig. 3d(iii)). As the central cavity of the knot contracts, the mobility of the ethylene glycol linkers becomes increasingly restricted, and deformation of the backbone increases. Notably, the C–O–C angle (denoted as α in Fig. 3a,c) containing the scissile bond is distorted to ~140° (from ~109° in the tension-free knot) and the naphthyl groups (denoted as β in Fig. 3a,c) are bent to ~145° (from ~180° in the tension-free knot) in the state of maximal deformation (Fig. 3a(ii),d(ii)). Ultimately, scission occurs at the C–O bond (denoted as a in Fig. 3a,b) of the external naphthyl group.

The models suggest a heterolytic scission in which the developing positive and negative charges, on the ethylene glycol carbon and naphthol oxygen, respectively, are stabilized by the pyridine lone pair and the amide NH (Fig. 3d(ii,iii)). The scission occurs where the strand exits the loop formed by the knot (Fig. 3d(ii)). This position is similar to that predicted computationally in previous studies^3,4^. The knot topology dramatically reduces the force required to break the otherwise unreactive covalent bond. The calculated force at maximum deformation (*F*_max_) is reduced from ≥5.6 nN for models of the linear ligand (Supplementary Sections 9.3 and 9.4) to 2.9 nN for the same ligand in a knotted topology, where the cleaved bond differs from the linear analogue (suggesting an even larger difference in reactivity between the scissile bond observed in the knot and the same bond in the linear ligand). In fact, the looped arrangement of the knot causes the activation of an otherwise unreactive covalent bond because of the force-induced bending and stretching deformations (see above)^17^. Remarkably, as a mechanophore the knot (*F*_max_ = 2.9 nN) is even more reactive than the Diels–Alder mechanophore used in the gate (*F*_max_ = 3.7 nN) and ranks among the most reactive scissile mechanophores described to date^49^.

Taken together, these results provide a picture of the overall process. As the chain-centred knot enters the flow field surrounding a collapsing cavitation bubble, the chain is stretched until the gating unit opens. This results in the initial closed-loop trefoil knot opening into an overhand knot. Unlike the closed-loop trefoil knot, the overhand knot is potentially able to expand and diffuse along the polymer chain^50^. However, the kinetic data indicate that opening of the gate and the knot scission occur in the same elongation event. In other words, the overhand knot formed after the gate opening does not have time to relax and rearrange in the high-strain environment of the cavitation field. Indeed, a cavitation bubble collapses much faster (microsecond timescale^51^) than the relaxation time of a knot (which, for example, occurs on the seconds timescale for DNA^41^). Moreover, knot diffusion along a strand is suppressed at relatively low tension^52^. Other dynamic ‘memory effects’ have previously been observed with cyclic polymers^53^. The implication for the current system is that the overhand knot is likely to contract around the central region, although not necessarily symmetrically, of the knotted strand (Fig. 1), a picture supported by the CoGEF simulations (Fig. 3). As the knot enters the final stage of its contraction, a large amount of backbone deformation (in the form of bond bending and stretching) is observed, conspicuously, via the substantial bending of the flanking naphthyl groups as well as at the naphthyl/ethylene glycol junction where the strand exits the knot cavity. Ultimately, this leads to the heterolytic scission of a C–O bond in the latter section, where the developing charges are stabilized by hydrogen bonding of the amide and the pyridine lone pair. This nature of the scissile bond was confirmed experimentally by isolation of the knot fragments post-sonication.

## Conclusions

Matter often behaves very differently at different length scales. For example, the friction and inertia that both maintain and localize knots in macroscopic strands do not do so at the nanoscale. However, we find that the stress distribution and scission point in a molecular overhand knot under stretching are very similar to that observed for knotted fishing lines and cooked spaghetti^2^; in other words, overhand knots induce closely related modes of strand weakening across molecular (nm), microscopic (μm)^14^ and macroscopic (mm)^1,2^ scales. The effect that knotting has on the mechanical strength of covalent bonds in a polymer chain is dramatic, reducing the scission force from ≥5.6 nN to 2.9 nN, which results in a scission rate at least 2.6× higher than an unknotted counterpart, producing one of the most reactive scissile mechanophores known. Knot activation is so effective that scission of the knotted polymer chain involves a different set of chemical bonds from those that break in an unknotted polymer under mechanical stress.

Knots are found extensively in biomacromolecules^10^ and form spontaneously^13,38,54–58^ in many synthetic polymers. However, the probability of finding a randomly formed knot is only significant at molecular masses >1 MDa (refs. ^13,38^). Many of the polymers that have previously been investigated by sonication are <200 kDa and so have a low frequency of being knotted. Our results provide experimental evidence that knotting may be intrinsically detrimental to the mechanical strength of high molecular mass and other knotted polymers. This study was carried out on polymers in solution, but we note that the order of scission rates of structural elements in solution mechanochemistry generally correlates with the order of their breaking rates in the condensed phase^39^.

## Methods

Detailed methods and protocols are given in the Supplementary Information.

### Mechanical activation

The polymer (15 mg) was dissolved in CH_3_CN (15 ml) and added to a modified Suslick cell. The solution was sonicated using a Sonics VCX 500 ultrasonic processor equipped with a 13-mm-diameter solid probe or replaceable-tip probe (20 KHz, 11.5 W cm^−2^, 1 s on/2 s off, 5–10 °C). Nitrogen was gently bubbled through the solution as it was sonicated. After 180 min of sonication time, the mixture was concentrated and dried under high vacuum for an extended period (~24 h); the polymer was washed with methanol (5 ml) before drying again.

### CoGEF calculation

CoGEF calculations were performed on Spartan 14/20 following Beyer’s method^48^. The knot was constructed in Spartan 14 and minimized using molecular mechanics (MMFF). The distance between the anchor groups was constrained and increased in increments of 0.5 Å. At each step, the energy was minimized by molecular mechanics (MMFF) with Spartan 14 and then density functional theory (UB3LYP/6-31G*, gas phase) with Spartan 20. The relative energy of each intermediate was determined by setting the energy of the unknotted state to 0 kJ mol^−^^1^. The *F*_max_ value was determined from the slope of the final 40% of the energy/elongation curve (that is, from 0.6*E*_max_ to *E*_max_, where *E*_max_ is the maximum relative energy immediately before bond rupture).

## Online content

Any methods, additional references, Nature Portfolio reporting summaries, source data, extended data, supplementary information, acknowledgements, peer review information; details of author contributions and competing interests; and statements of data and code availability are available at 10.1038/s41557-024-01510-3.

### Supplementary information

Supplementary information Supplementary Figs. 1–9, experimental procedures and characterization data, Schemes 1–6, Tables 1 and 2, and Spectra 1–75.

### Source data

Source Data Fig. 2 Dissociation kinetics over time.

Source Data Fig. 3 CoGEF simulation over time.

## Data Availability

The data that support the finding of this study are available within the paper and its Supplementary Information or are available from the figshare data repository: 10.6084/m9.figshare.21548319 (ref. ^59^). Source data are provided with this paper.
