# Supplementary material for: Mechanical scission of a knotted polymer
Source: Nat Chem. 2024 Apr 22;16(8):1366–72. doi: 10.1038/s41557-024-01510-3 (PMC11321991; doi:10.1038/s41557-024-01510-3)
Supplement: Supplementary file 1 — Supplementary Figs. 1–9, experimental procedures and characterization data, Schemes 1–6, Tables 1 and 2, and Spectra 1–75. [file 41557_2024_1510_MOESM1_ESM.pdf]

nature chemistry

Article

https://doi.org/10.1038/s41557-024-01510-3

Mechanical scission of a knotted polymer

In the format provided by the authors and unedited

Table of Contents

Table of Contents 1

1 Abbreviations and nomenclature 2

2 General experimental details 3

3 Reaction schemes 5
- 3.1 Synthesis of gate **S9** 5
- 3.2 Synthesis of knot ligand **S16** 6
- 3.3 Synthesis of linear ligand **S20** 7
- 3.4 Synthesis of gated trefoil knot **S23** 8

4 Additional data 9
- 4.1 General procedure for sonication experiments 9
- 4.2 Sonication experiments of gate polymer **2** 10
- 4.3 Sonication experiments of linear ligand polymer **3** 11
- 4.4 Sonication experiments of gated knot polymer **1** 12
- 4.5 Polymer chain detachment on linear ligand **3** 13
- 4.6 Hydrolysis of the gated knot **S23** 14
- 4.7 Detachment of the fragments from the sonicated samples 15
- 4.8 MS spectra of the detached fragments 17
- 4.9 NMR spectra and MS spectra of the isolated fragments **4** and **6** 20
- 4.10 MS of sonicated solution before hydrolysis 23
- 4.11 Representative NMR spectra of sonicated polymers 24

5 Synthetic procedures 26
- 5.1 Synthetic procedures and characterization details 26
- 5.2 Demetallation of trefoil knot Lu-3_1 **S23** 37

6 Polymers 39
- 6.1 Representative procedure for the synthesis of mechanophore-linked PMA 39
- 6.2 List of polymers synthesized 39

7 MS spectra 41

8 NMR Spectra 44

9 CoGEF Calculations 69
- 9.1 General procedure 69
- 9.2 Gate model 69
- 9.3 Ligand model 1 70
- 9.4 Ligand model 2 71
- 9.5 Knot 72
  - 9.5.1 MMFF 72
  - 9.5.1 DFT (short model) 73

10 References 75

1 Abbreviations and nomenclature

Abbreviations: COSY correlation spectroscopy; DCM dichloromethane; DEPT distortionless enhancement by polarization transfer; DMF *N*,*N*-dimethylformamide; DMSO dimethylsulfoxide; DOSY diffusion-ordered spectroscopy; HMBC heteronuclear multiple-bond correlation; HSQC heteronuclear single quantum coherence; MALDI-TOF matrix assisted laser desorption/ionization time-of-flight; MeCN acetonitrile; PE petroleum ether; pdc 2,6-pyridine dicarboxamide; DIPEA N,N-diisopropylethylamine; EDCI *N*-(3-Dimethylaminopropyl)-*N′*-ethylcarbodiimide hydrochloride; EDTA ethylenediaminetetraacetic acid; DMAP 4-(Dimethylamino)pyridine; Me_6TREN Tris [2- (dimethylamino)ethyl]amine; RT room temperature; GPC gel permeation chromatography; TLC thin layer chromatography; CoGEF constrained geometries simulate external force; MMFF molecular mechanics force field.

2 General experimental details

Unless stated otherwise, reagents were obtained from commercial sources and used without purification. Reactions were carried out in anhydrous solvents and under an N_2 atmosphere. Anhydrous solvents were obtained by passing the solvent through an activated alumina column on a Phoenix SDS (solvent drying system; JC Meyer Solvent Systems, CA, USA). Cu(0) wire (diameter: 0.25 mm, purity 99.9%) was purchased from Sigma-Aldrich. Me_6TREN was purchased from Alfa Aesar. Compounds **S4**[1], **S10**[2] and **S17**[3] were synthesized as previously described.

Gel permeation chromatography (GPC) analyses were performed in THF solution (~0.6 mg mL^{-1}) at 35 °C using a GPC Agilent 1260 Infinity II with 2 × PL gel 10 µm mixed-B and a PL gel 500 Å column, and equipped with a differential refractive index (DRI) detector employing narrow polydispersity polystyrene standards (Agilent Technologies) as a calibration reference. Samples were filtered through a Whatman Puradisc 4 mm syringe filter with 0.45 µm PTFE membrane before injection to equipment, and experiments were carried out with injection volume of 100 µL, flow rate of 1 mL min^{-1}. Results were analyzed using Malvern OmniSEC 5.10 software.

Ultrasound experiments were performed using a Sonics VCX 500 ultrasonic processor equipped with a 13 mm diameter solid or replaceable-tip probe. The distance between the titanium tip and the bottom of the Suslick cell was 2 cm. The ultrasonic intensity was calibrated using the method outlined by Hickenboth *et al.*[4] The Suslick cells were fabricated by the Department of Chemistry glass workshop at the University of Manchester.

^{1}H NMR and ^{13}C NMR spectra were recorded on a Bruker Avance III instrument with an Oxford AS600 magnet equipped with a cryoprobe [5 mm CPDCH ^{13}C-^{1}H/D] (600 MHz) or a Bruker Avance III 400 MHz Prodigy instrument at the University of Manchester. Chemical shifts are reported in parts per million (ppm) from high to low frequency using the residual solvent peak as the internal reference ($CDCl_3$ = 7.26 ppm, $(CD_3)_2CO$ = 2.05 ppm, MeOD-d_4 = 3.31 ppm, DMSO-d_6 = 2.50 ppm and MeCN-d_3 = 1.94 ppm). All ^{1}H resonances are reported to the nearest 0.01 ppm. The multiplicity of ^{1}H signals are indicated as: s = singlet; d = doublet; t = triplet; q = quartet; m = multiplet; br = broad; app = apparent; or combinations thereof. Coupling constants (J) are quoted in Hz and reported to the nearest 0.1 Hz. Where appropriate, averages of the signals from peaks displaying multiplicity were used to calculate the value of the coupling constant. ^{13}C NMR spectra were recorded on the same 600 MHz or 400 MHz spectrometer with the central resonance of the solvent peak as the internal reference (MeCN-d_3 = 118.26 ppm, $CDCl_3$ = 77.16 ppm, $(CD_3)_2CO$ = 29.84 ppm, MeOD-d_4 = 49.00 ppm and DMSO-d_6 = 39.52 ppm). All ^{13}C resonances are reported to the nearest 0.01 ppm. DEPT, COSY, HSQC and HMBC experiments were used to aid structural determination and spectral assignment. Fully characterized compounds were chromatographically homogeneous.

Flash column chromatography was carried out using Silica 60 Å (particle size 40–63 µm, Sigma Aldrich, UK) as the stationary phase. Size exclusion chromatography was carried out using Biobeads S-X1 (CH_2Cl_2) support beads as the stationary phase. Preparative TLC was performed on precoated silica gel plates: 2 mm, UNIPLATE GF, Analtech Inc., DE, USA. Analytical TLC was performed on precoated silica gel plates (0.25 mm thick, 60 F_{254}, Merck, Germany) and visualized using both short and long wave ultraviolet light in combination with standard laboratory stains (basic potassium permanganate, acidic ammonium molybdate and

ninhydrin). Low resolution ESI mass spectrometry was performed with a Thermo Scientific LCQ Fleet Ion Trap Mass Spectrometer or an Agilent Technologies 1200 LC system with either an Agilent 6130 single quadrupole MS detector or an Advion Expression LCMS single quadrupole MS detector. High resolution mass spectrometry was performed on a Thermo Exactive Plus Orbitrap Extended Mass Range mass spectrometer. MALDI-TOF was performed on a Shimadzu Biotech Axima Confidence Matrix Assisted Laser Desorption Ionisation mass spectrometer. High-resolution mass spectrometry (HR-MS) and MALDI-TOF (matrix assisted laser desorption/ionization time-of-flight) were carried out by staff at the Mass Spectrometry Service, Department of Chemistry, University of Manchester.

3 Reaction schemes

3.1 Synthesis of gate S9

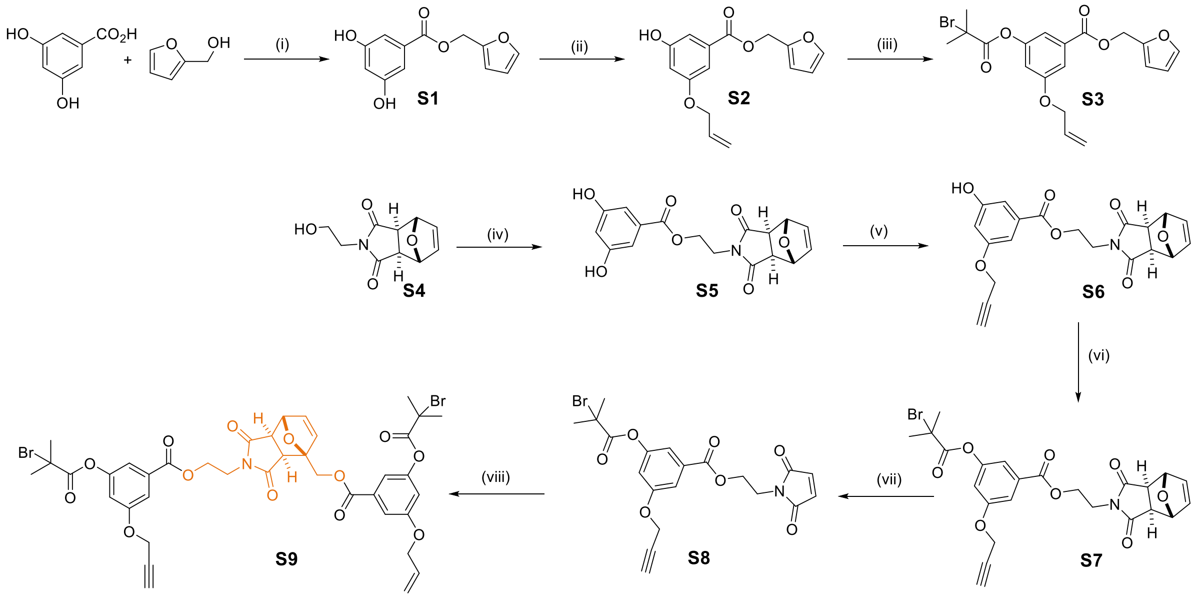

Scheme 1. Synthesis of gate **S9**. Reagents and conditions: (i) EDCI, DMAP, 60 °C, 4 h, 15%. (ii) 3-Bromo-1-propene, K_2CO_3, MeCN, 80 °C, 4 h, 31%. (iii) α-Bromoisobutyryl bromide, Et_3N, DCM, 0 °C → RT, 2 h, 91%. (iv) EDCI, DMAP, DMF, 80 °C, 16 h, 10%. (v) Propargyl bromide, K_2CO_3, MeCN, 80 °C, 4 h, 26%. (vi) α-Bromoisobutyryl bromide, Et_3N, DCM, 0 °C -> RT, 2 h, 82%. (vii) Toluene, 105 °C, 16 h, quantitative. (viii) **S3**, toluene, 75 °C, 48 h, 41%.

3.2 Synthesis of knot ligand S16

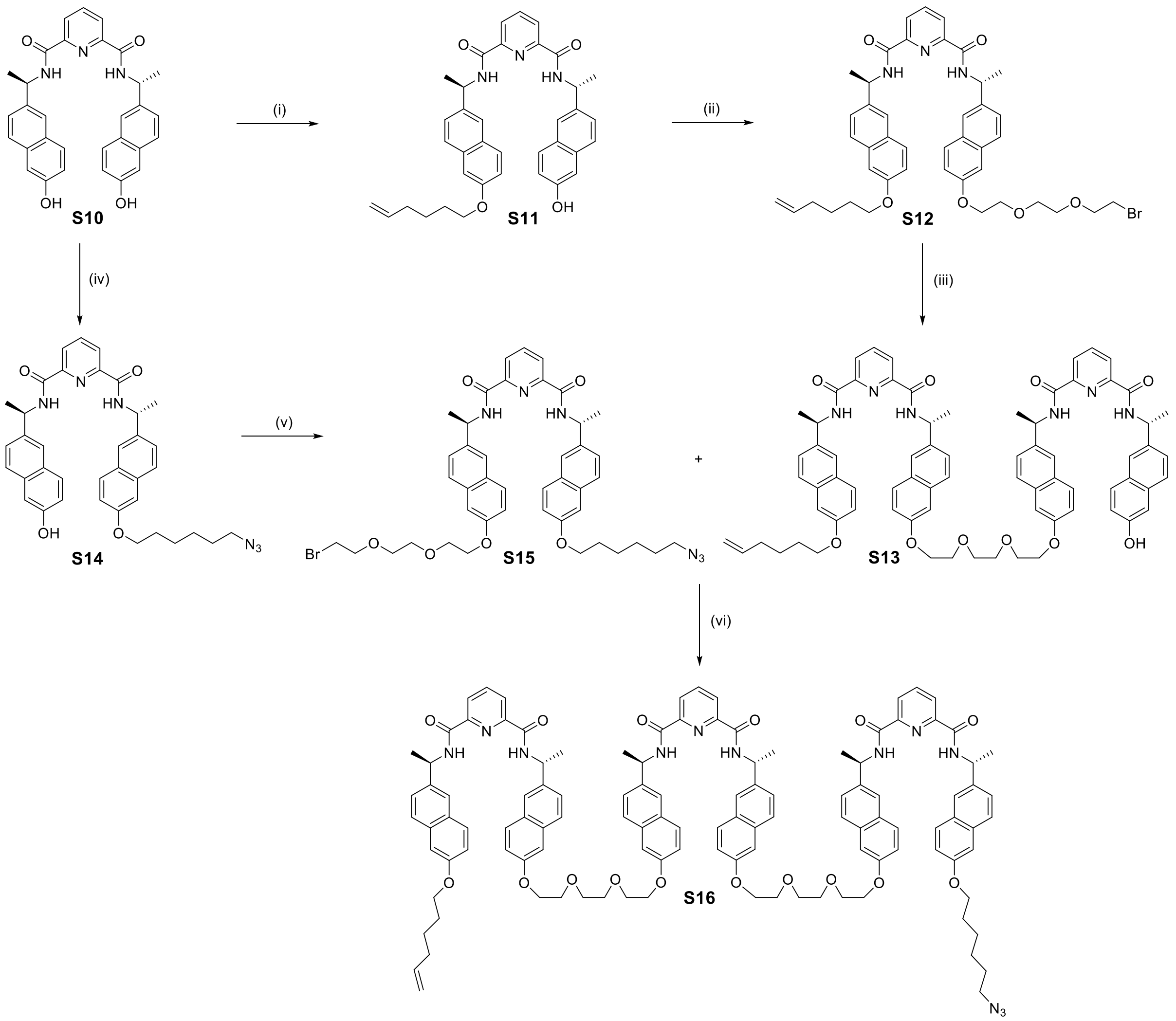

Scheme 2. Synthesis of knot ligand **S16**. Reagents and conditions: (i) 6-Bromo-hex-1-ene, K_2CO_3, 80 °C, 4 h, 35%. (ii) 1,2-Bis(2-bromoethoxy)ethane, K_2CO_3, RT, 18 h, 75%. (iii) **S10**, K_2CO_3, 80 °C, 16 h, 22%. (iv) 1-Azido-6-bromohexane, K_2CO_3, 80 °C, 4 h, 21%. (v) 1,2-Bis(2-bromoethoxy)ethane, K_2CO_3, MeCN, RT, 18 h, 76%. (vi) K_2CO_3, 80 °C, 16 h, 80%.

3.3 Synthesis of linear ligand S20

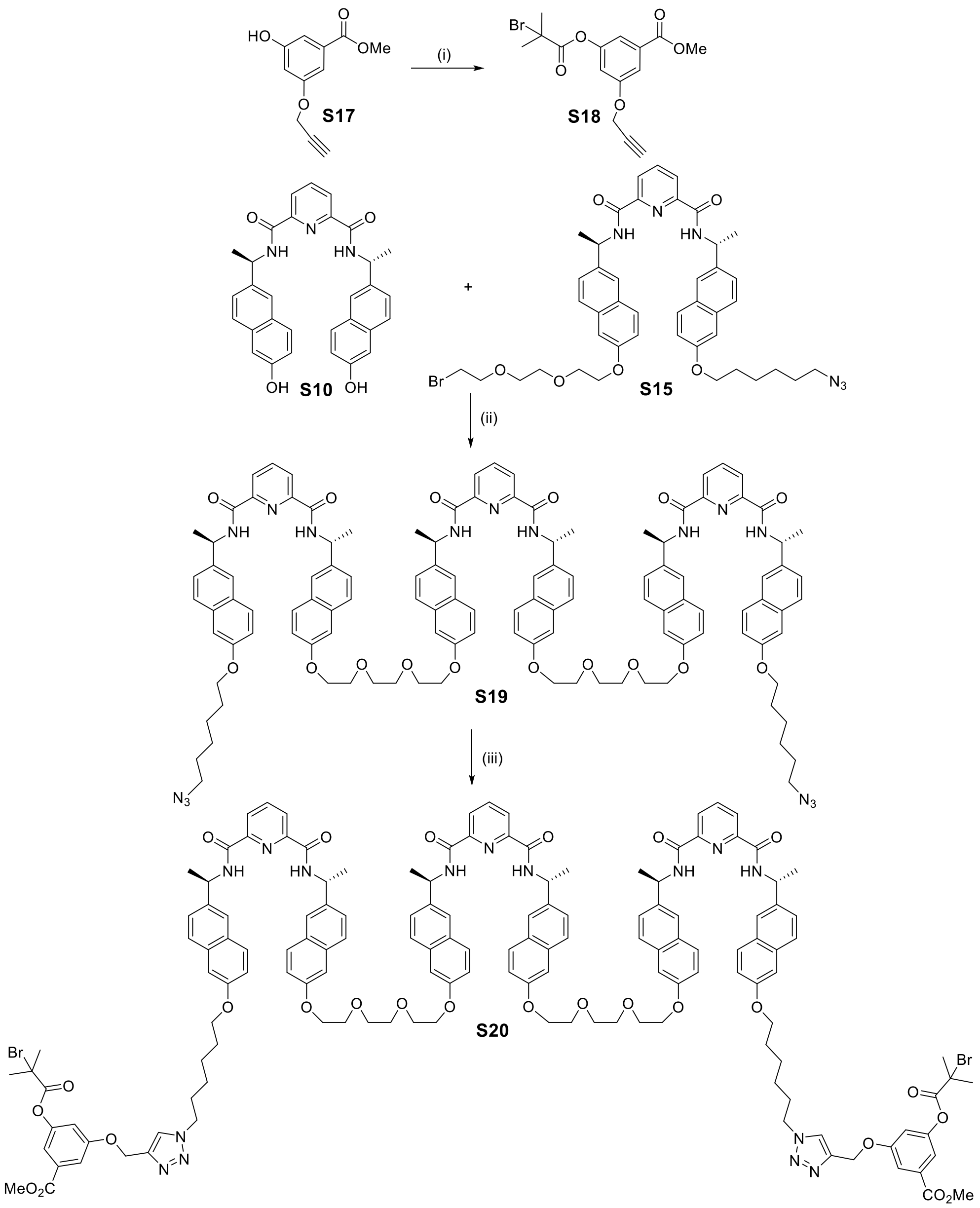

Scheme 3. Synthesis of the linear ligand **S20**. Reagents and conditions: (i) α-Bromoisobutyryl bromide, Et_3N, DCM, 0 °C → RT, 2 h, 87%. (ii) K_2CO_3, DMF, 80 °C, 16 h, 80%. (iii) **S18**, $Cu(MeCN)_4PF_6$, DCM/MeCN (*v*/*v* = 10:1), RT, 16 h, 60%.

3.4 Synthesis of gated trefoil knot S23

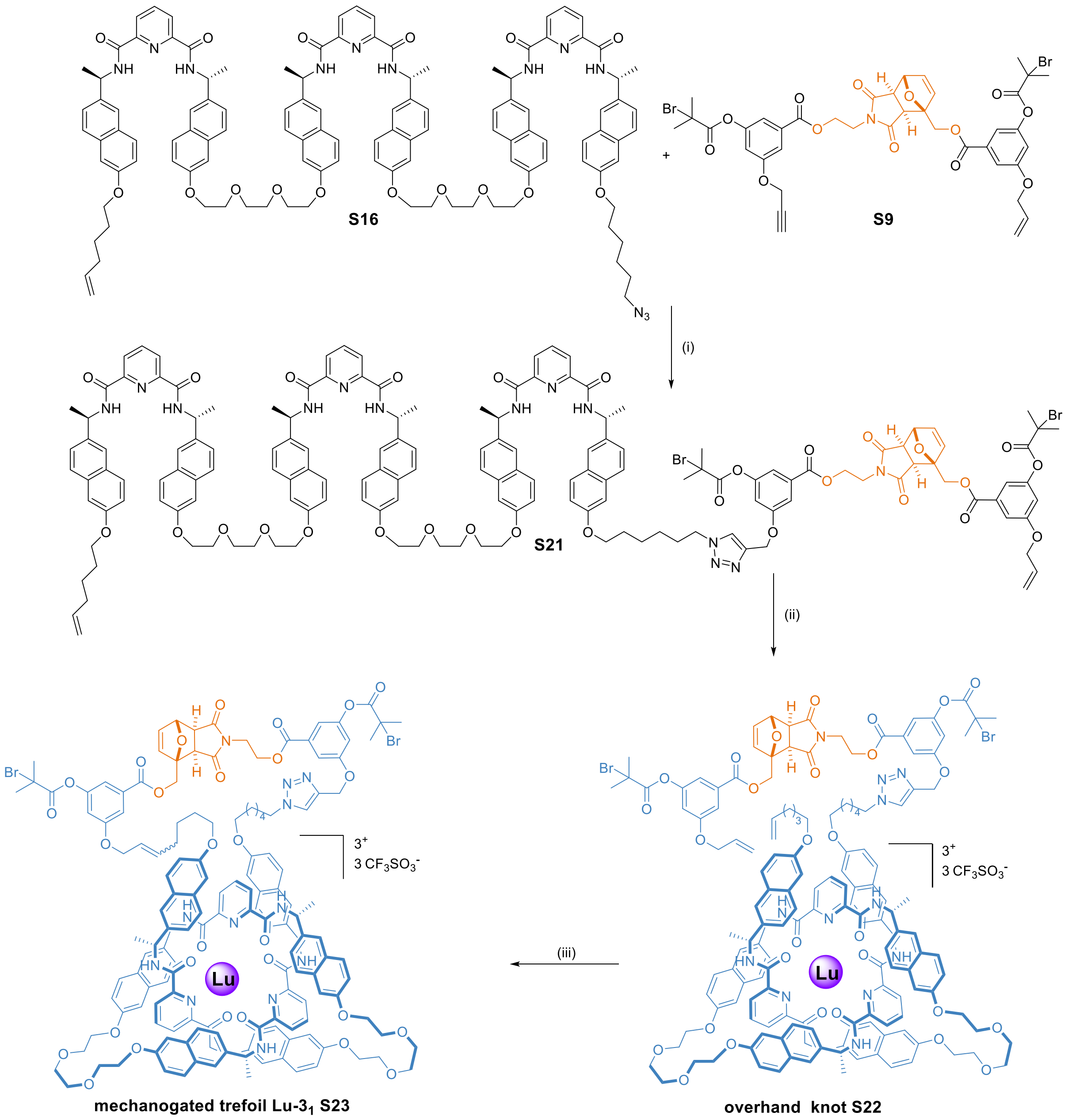

Scheme 4. Synthesis of gated trefoil knot Lu-3_1 **S23**. Reagents and conditions: (i) $Cu(MeCN)_4PF_6$, DCM/MeCN (*v*/*v* = 10:1), RT, 16 h, 70%. (ii) $Lu(CF_3SO_3)_3$, MeCN, 70 °C, 16 h, 95%. (iii) Hoveyda-Grubbs 2nd generation catalyst, CH_3NO_2/CH_2Cl_2 1:1, 50 °C, 16 h, 25%.

4 Additional data

4.1 General procedure for sonication experiments

The relevant polymer (15 mg) was added to a Suslick cell and dissolved in anhydrous MeCN (15 mL). The solution was degassed by purging with a N_2 stream for a minimum of 10 min prior to the start of sonication, and bubbling was maintained throughout the experiment. The three arms of the Suslick cell were sealed with NMR tube septa. The cell was cooled with an ice bath throughout the duration of the sonication to maintain a temperature of ~5-10 °C. Pulsed ultrasound was applied to the system (1s on, 2s off, 25% amplitude [11.5 W cm^{-2}], 20 kHz). Aliquots of 300 µL were withdrawn from the cell at a minimum of every 30 min sonication time; the solvent in these aliquots was evaporated off under a stream of N_2 and the resulting residue redissolved in 500 µL of THF. Each sample was then filtered through a syringe filter (PTFE, 0.45 µm pore size) and analysed by GPC.

After 180 min sonication time, the solvent of the sonication mixture was evaporated and the remaining polymer residue washed with MeOH. The sample was then analyzed by NMR spectroscopy.

4.2 Sonication experiments of gate polymer 2

Sonication of the gate polymer **2** was performed using the procedure described in section 4.1. The polymer underwent retro-Diels-Alder reactions under high tension (Supplementary Figure 1). By comparing the NMR of the pre- and post-sonication samples with the two reference polymers **S30** and **S31**, the appearance of maleimide peak H_a (6.72 ppm) and furan peaks H_e (6.46 ppm) and H_d (6.36 ppm) can easily be identified. Accordingly, the CH_2 linkers shift dramatically, as can be seen from the shift of H_g shifts from 5.18 ppm and 4.68 ppm to 5.26 ppm.

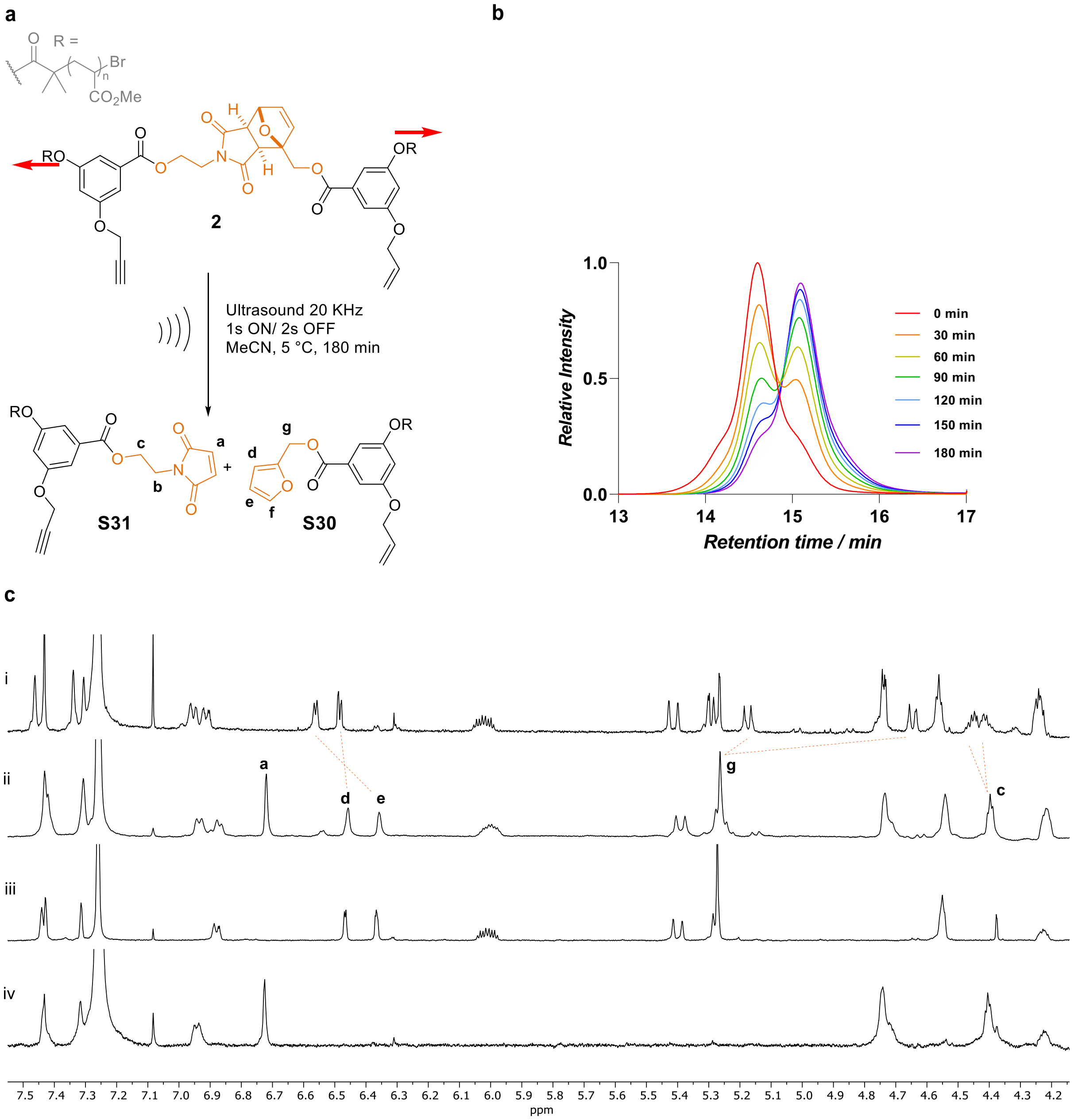

Figure 1. a) Sonication of gate polymer **2** (after 180 min). b) GPC analysis of the mixture over the course of the sonication event. c) Truncated ^1H NMR spectra (600 MHz, $CDCl_3$) of the pre- (i) and post- (ii) sonication samples, as well as reference polymers **S30** (iii) and **S31** (iv).

4.3 Sonication experiments of linear ligand polymer 3

Sonication of the linear ligand polymer **3** was performed using the procedure described in section 4.1. The NMR spectra of the pre- and post-sonication samples were almost identical, which combined with the calculations shown in (section 9.3 and 9.4), indicates that the ligand structure is inert under force. A small quantity of cleavage observed via GPC analysis suggests the polymer is cleaved randomly along the backbone.

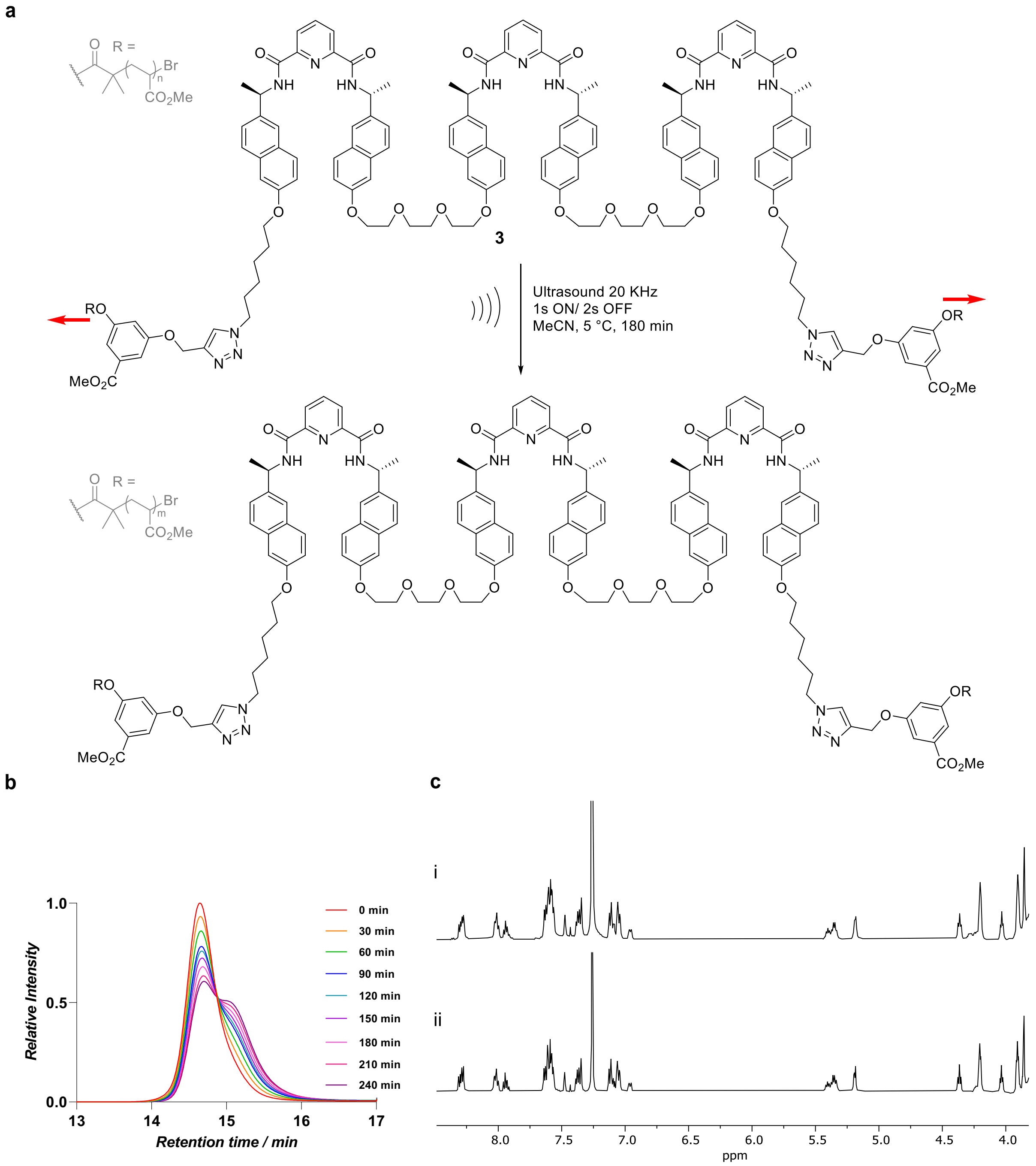

Figure 2. a) Sonication of linear ligand polymer **3** (after 240 min). b) GPC analysis of the mixture over the course of sonication. c) ^{1}H NMR (600 MHz, $CDCl_3$) comparison of the pre- (i) and post- (ii) sonication samples.

4.4 Sonication experiments of gated knot polymer 1

Sonication of the gated knot polymer **1** was performed using the procedure described in section 4.1. GPC analysis showed the polymer cleaved at a rate indicative of the inclusion of a mechanically weak moiety. By comparing ^{1}H NMR spectra of the pre- and post-sonication samples, the appearance of the maleimide protons at 6.68 ppm and the furan protons at 7.42 ppm, 6.47 ppm and 6.35 ppm (see Supplementary Figure 3c), as well as the disappearance of the Diels-Alder adduct double bond protons (around 6.5-6.6 ppm, see the red box range in Supplementary Figure 3d) indicate that the gate has been cleaved via retro-Diels-Alder reactions. However, these complex spectra are populated by too many overlapping peaks to confidently identify any further cleavage sites.

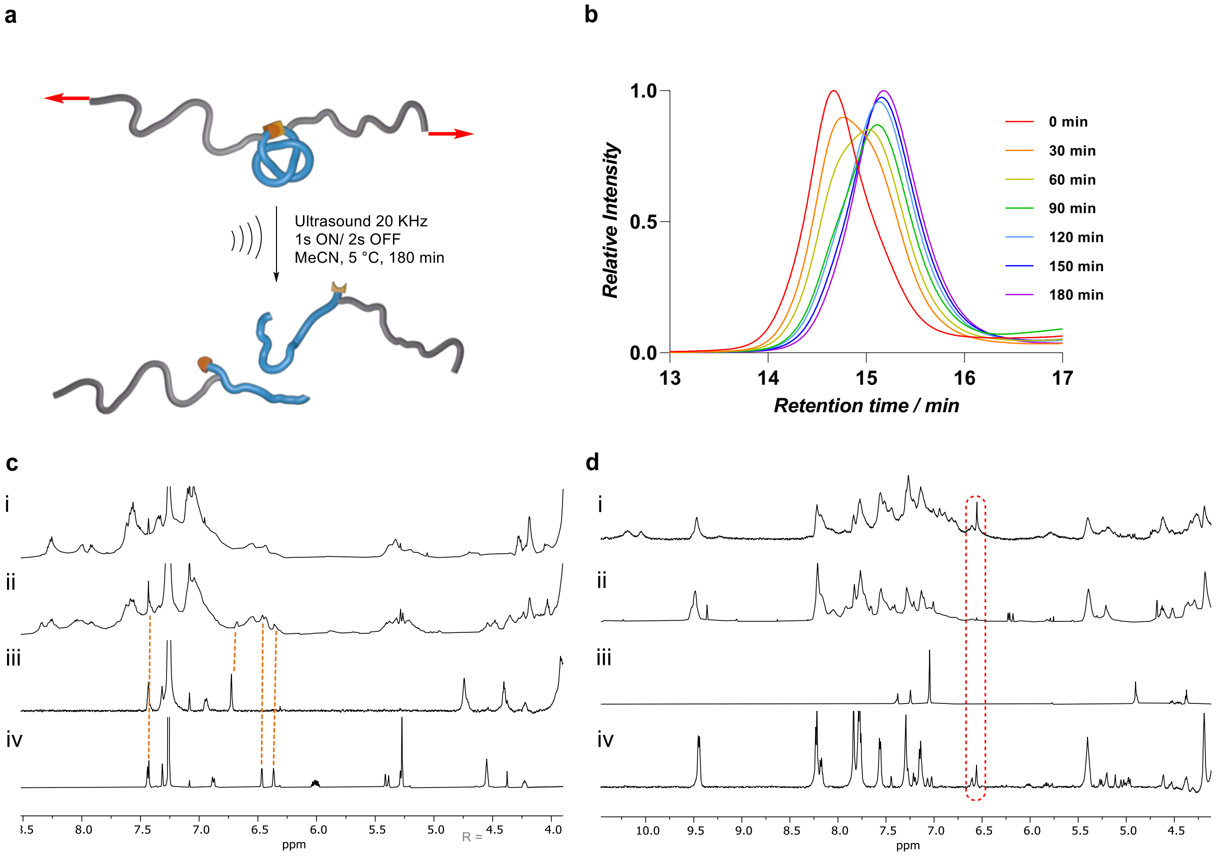

Figure 3. a) Sonication of gated knot polymer **1** (after 180 min). b) GPC analysis of the mixture over the course of the sonication event. c) ^{1}H NMR (600 MHz, $CDCl_3$) comparison of the pre- (i) and post- (ii) sonication samples, and the reference polymers **S31** (iii) and **S30** (iv). The maleimide protons at 6.7 ppm was appeared after sonication, as well as the furan protons at 7.45 ppm, 6.48ppm and 6.37 ppm. d) ^{1}H NMR (600 MHz, DMSO-d_6) comparison of the pre- (i) and post- (ii) sonication samples, as well as the reference polymers **S31** (iii) and **S32** (iv).The DA adduct double bond protons at 6.56 ppm and 6.61 ppm were disappeared after sonication.

Comparison of sonication kinetics for the three polymers described in Section 4.2-4.4 (Supplementary Table 1), demonstrates that the molecular knot (**1**) cleaves much faster than its linear analogue (**3**). This is also consistent with simulation data.[5]

Table 1: M_n/M_p, k*and conversion values for polymers **1**, **2** and **3**. k* is defined as the slope of the linear fit in Figure 2f. GPC conversion was calculated based on the GPC traces before and after sonication. [a] Sonication for 180 min. [b] Sonication for 240 min.

Polymer	M_n / M_p (kDa)	k* / $min^{-1}\cdot kDa^{-1}\cdot 10^5$				GPC Conversion (%)
		Run 1	Run 2	Run 3	Average	
1	62 / 77	8.10	7.93	8.21	8.08	95 ± 2[a]
2	71 / 82	8.00	7.51	7.60	7.70	97 ± 2[a]
3	67 / 80	2.95	3.10	2.96	3.00	52 ± 3[b]

4.5 Polymer chain detachment on linear ligand 3

The detachment of the homopolymer parts from the knot ligand fragments in the post-sonication sample was done by hydrolysis under alkaline conditions following a modified procedure.[6] This procedure was initially optimized on the linear analogue polymer as follows: linear ligand **3** (50 mg) was dissolved in THF/H_2O (4 mL, *v*/*v* = 3:1). NaOH (137 mg, 6 equivalents per monomer unit) was added and the mixture was stirred at 50 °C overnight. The solvent was removed, and the resulting residue acidified by 1M HCl to pH = 3. The mixture was extracted with DCM (5 x 5 mL). The organic phases were combined and concentrated. MALDI-TOF indicates successful detachment of the analogue ligand from the polymer, along with full ligand stability under the hydrolysis conditions with no fragmentations observed.

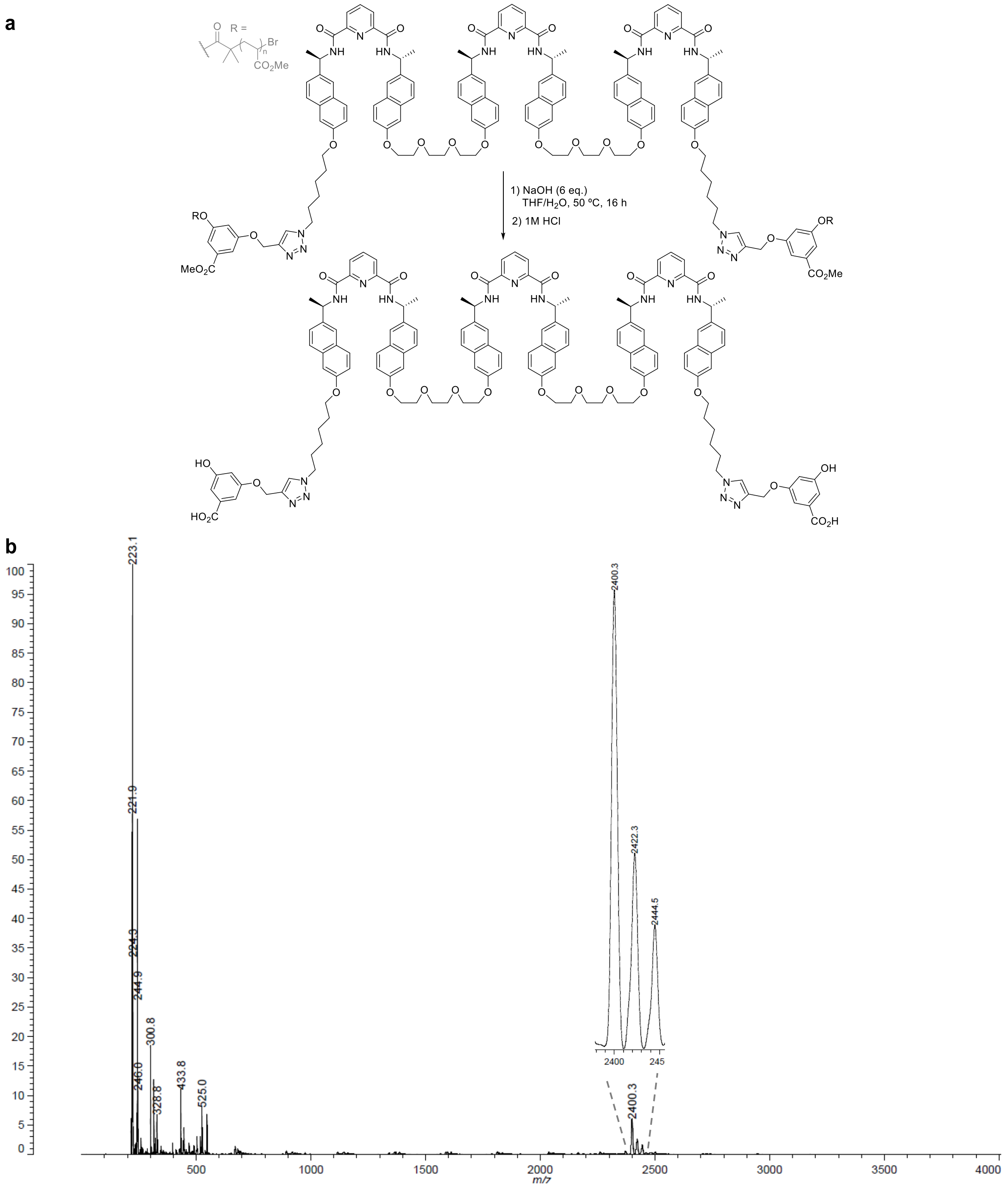

Spectrum 1. a) Detachment of the chain-centered ligand from linear ligand polymer **3** by hydrolysis. b) MALDI-TOF MS (positive mode, α-Cyano-4-hydroxycinammic acid matrix) of the detached ligand. Peak observed as the $[M+Na]^+$, $[M-H+2Na]^+$ and $[M-2H+3Na]^+$ adducts. Calculated peaks (*m*/*z*): 2401.0, 2423.0, 2445.0.

4.6 Hydrolysis of the gated knot S23

The control experiment on hydrolysis of the gated knot was done using the conditions described in section 4.5 to test the stability of the knotted structure under this alkaline environment. Gated knot **S23** (5 mg) was dissolved in THF/MeOH (1 mL, *v*/*v* = 4:1), NaOH (0.6 mg, in 0.3 mL H_2O) was added and the mixture was stirred at 50 °C overnight. The solvent was removed, and the resulting residue acidified by 1M HCl to pH = 3. The mixture was extracted with DCM (5 x 5 mL). The organic phases were combined and concentrated. HRMS identified the product with one of the two carboxyl functional groups forming the methyl ester (The ester was likely formed either during the reaction process or the work up step in presence of MeOH). This shows successful hydrolysis of the knot without any fragmentation being detected, which indicates the knotted structure itself is inert under this basic condition.

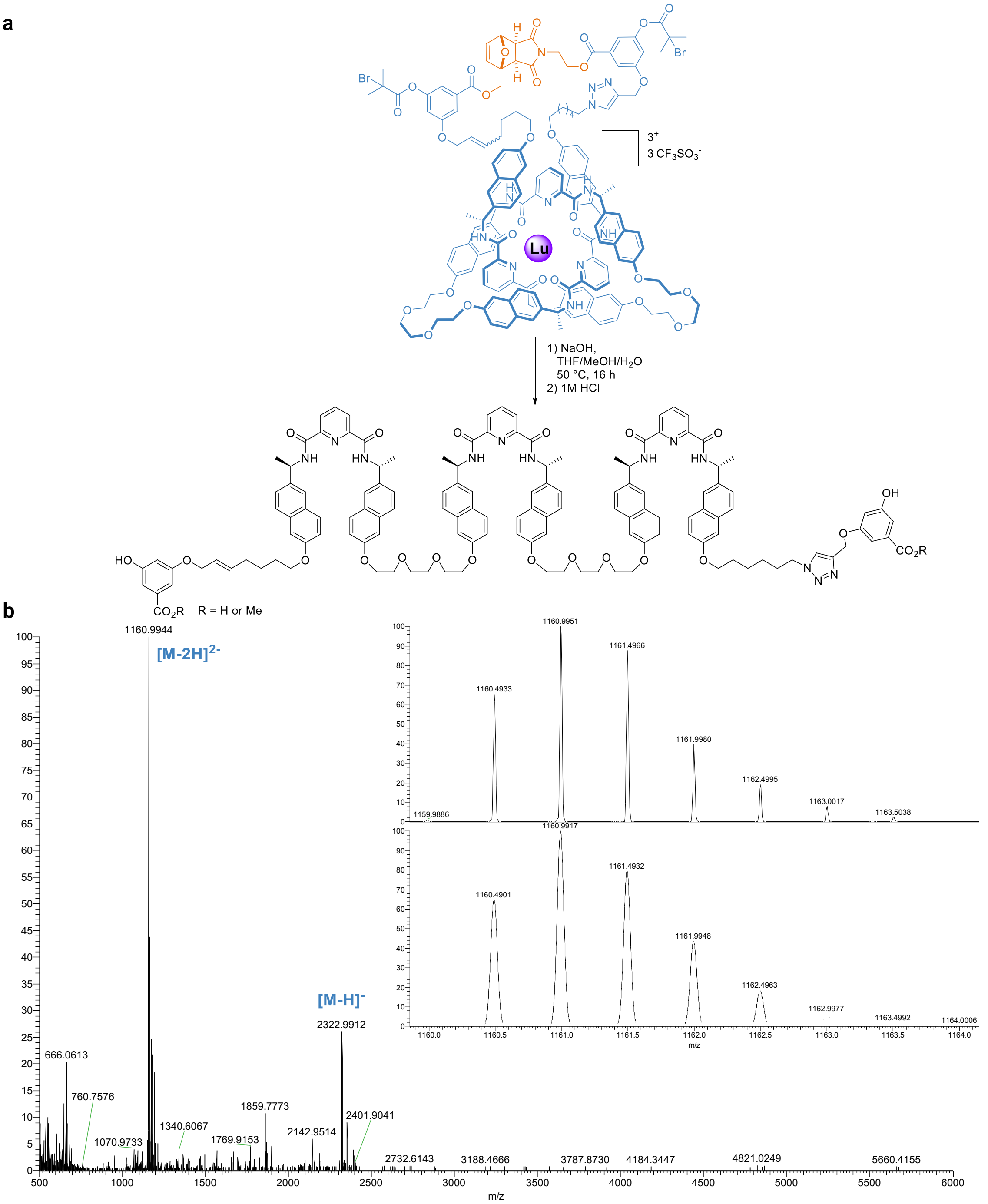

Spectrum 2. a) Hydrolysis of gated knot **S23**. b) High-resolution ESI-MS (-) of the hydrolyzed sample. Insert is the isotopic distribution of the major peak at 1160.9944. Top: Measured isotopic distribution for $C_{136}H_{136}N_{12}O_{24}$ ([M-2H]$^{2-}$, -ESI). Bottom: Simulated isotopic distribution for $C_{136}H_{136}N_{12}O_{24}$.

4.7 Detachment of the fragments from the sonicated samples

The detachment of fragments from the post-sonication sample of knotted polymer **1** was performed using the procedure outlined in section 4.5. A larger quantity of polymer (120 mg) was used for this purpose to more easily detect any fragment species. Initially, the collected organic phase from the detachment procedure was analyzed by HRMS. From this analysis, three main species including compounds **4**, **5** and **6** have been identified, compound **S25** has also been detected albeit with a relatively low intensity. All of these are indicative of mechanical cleavage of the C-O bond next to one of the napthyl group. Due to the pseudo-symmetry of the knot, any of the four C-O bond could be cleaved during a single stretching event, meaning there are at least 8 potential fragments (when alternative cleavage pathways, such as homolytic or heterolytic bond scission, are taken into account there may naturally also be more fragments). Further purification by preparative-TLC (10% MeOH in DCM) allowed isolation of two of the fragments in pure form. ^{1}H NMR spectroscopy and HRMS allowed assignment of the identity of these two fragments as compounds **4** and **6**, respectively.

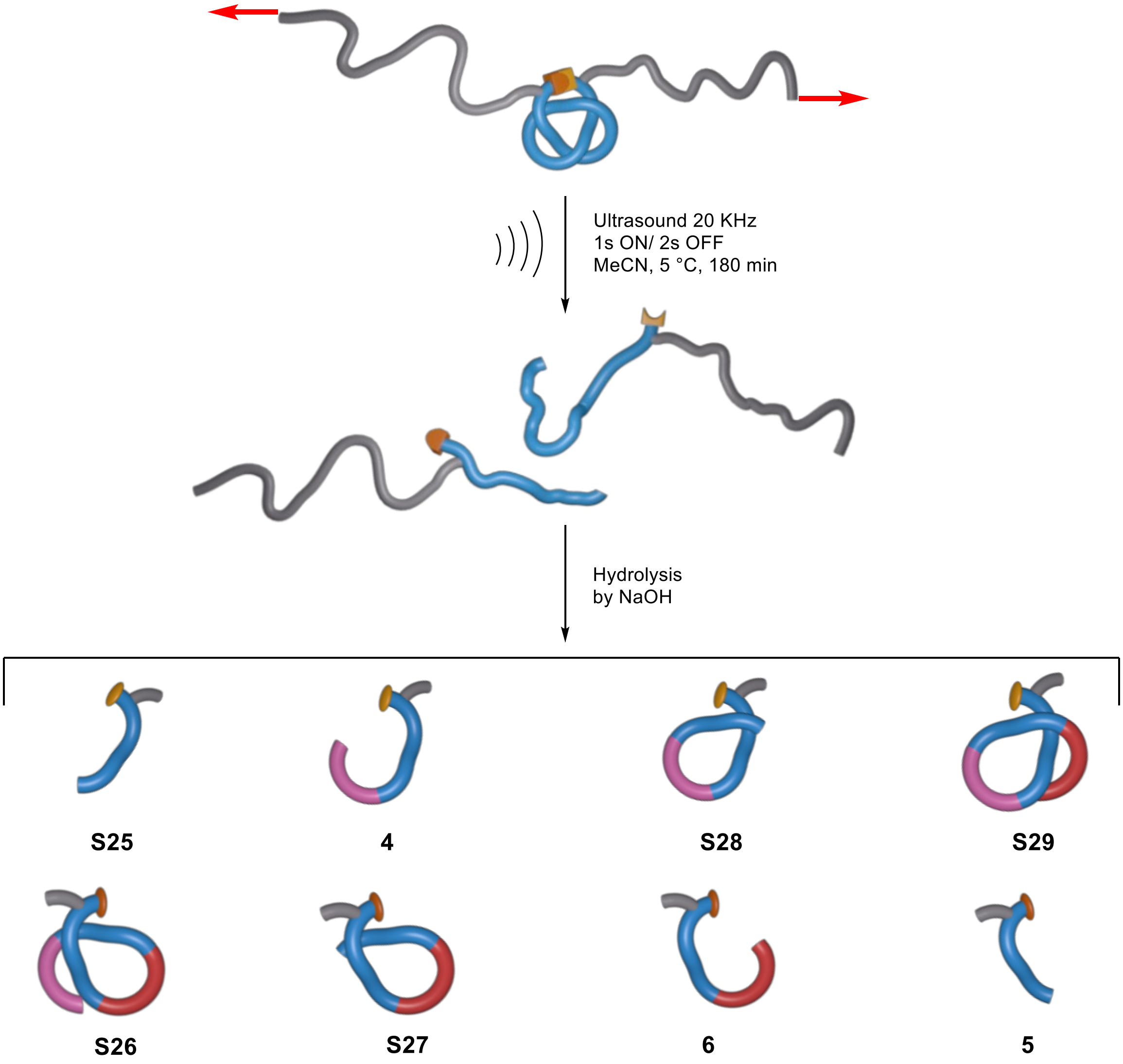

Scheme 5. Cartoon illustrations of the detachment of the fragments from post-sonication gated knot polymer **1**.

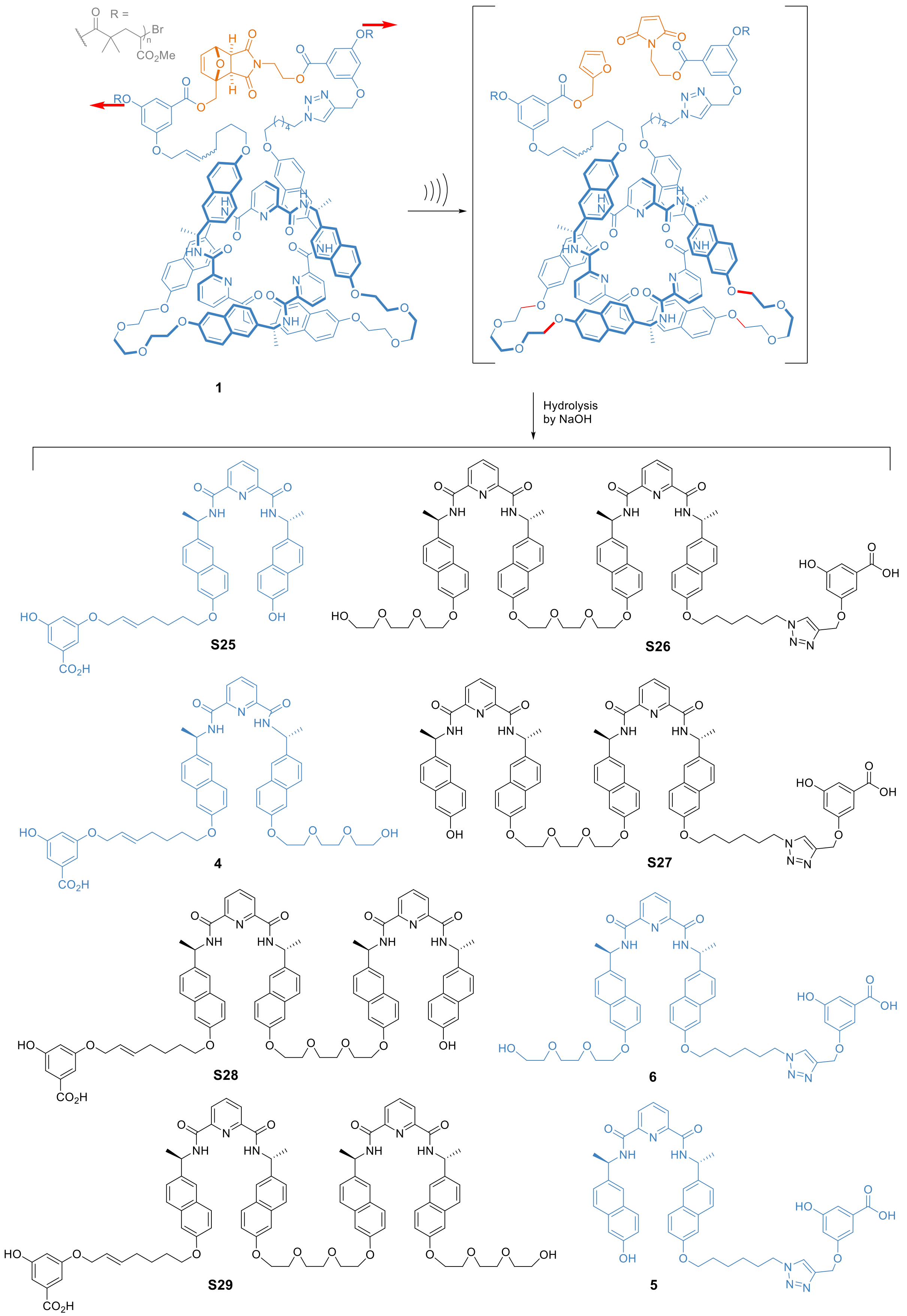

Scheme 6. Detachment of the fragments from post-sonication gated knot polymer **1**. The red-coloured bonds are the scissile bonds. The blue-coloured species have been detected by HRMS.

4.8 MS spectra of the detached fragments

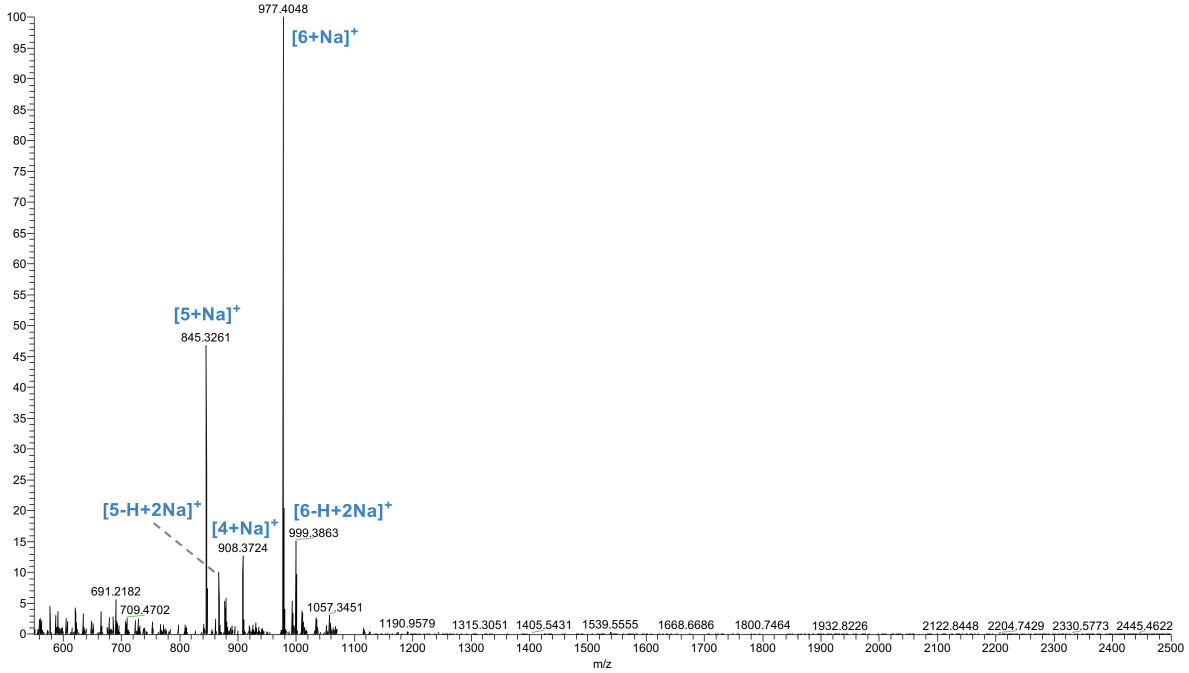

Spectrum 3. High-resolution ESI-MS(+) of the detached fragments obtained via the process described in Section 4.7.

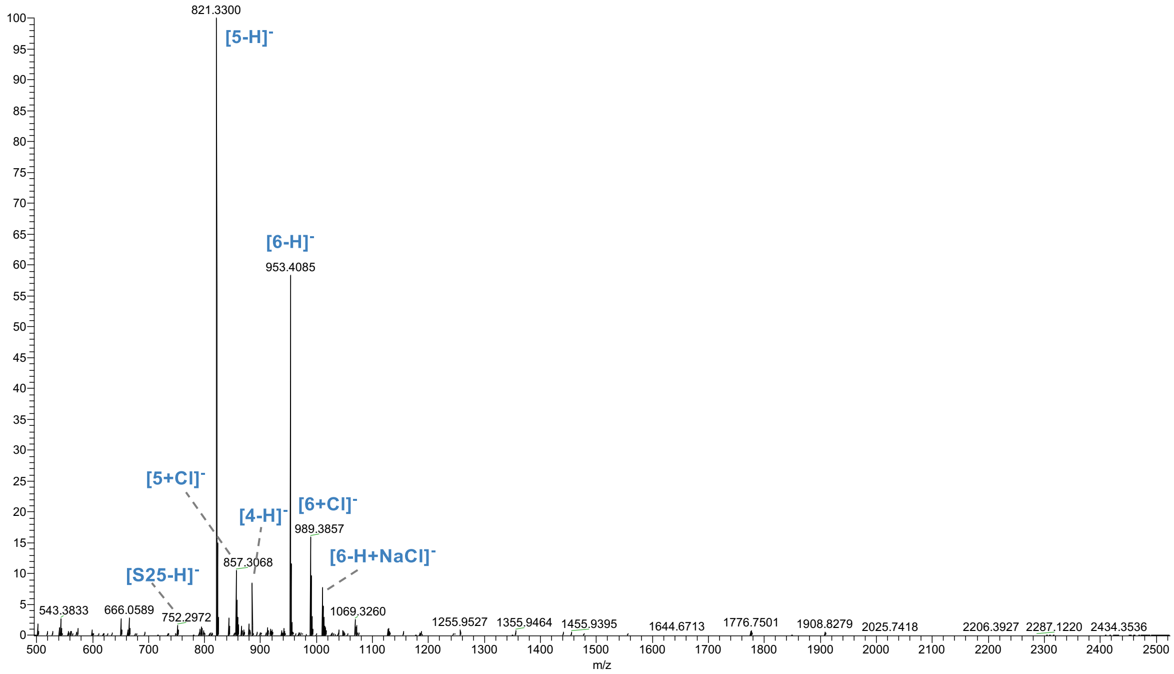

Spectrum 4. High-resolution ESI-MS(-) of the detached fragments obtained via the process described in Section 4.7.

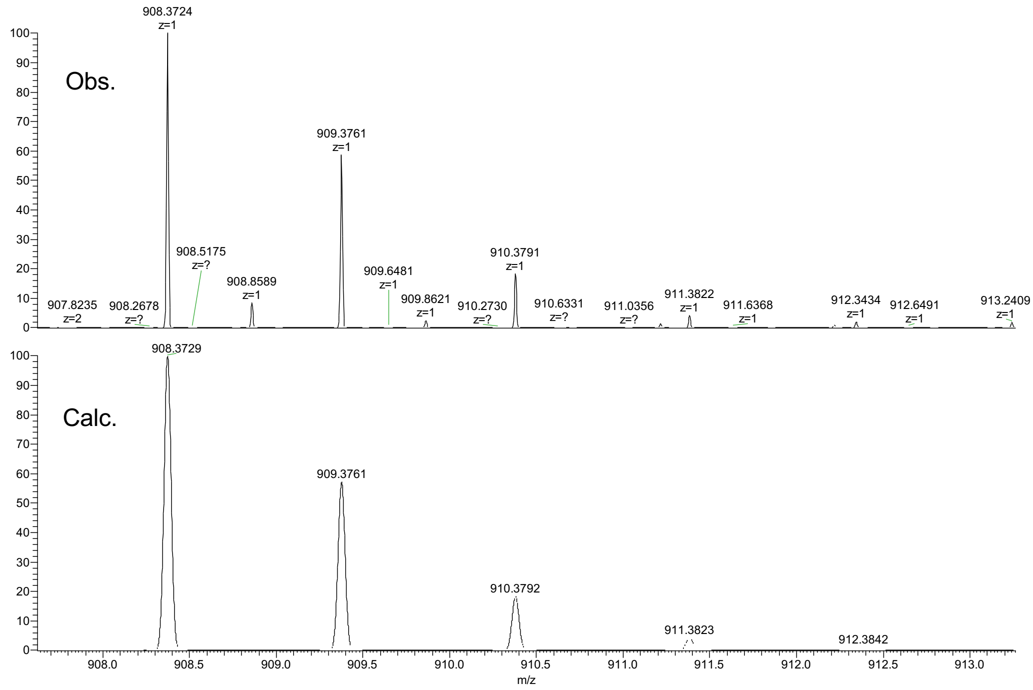

Spectrum 5. High-resolution ESI-MS isotopic distribution of the fragment **4**. Top: Measured isotopic distribution for $C_{51}H_{55}N_3O_{11}Na$ ($[M+Na]^+$, +ESI). Bottom: Simulated isotopic distribution for $C_{51}H_{55}N_3O_{11}Na$.

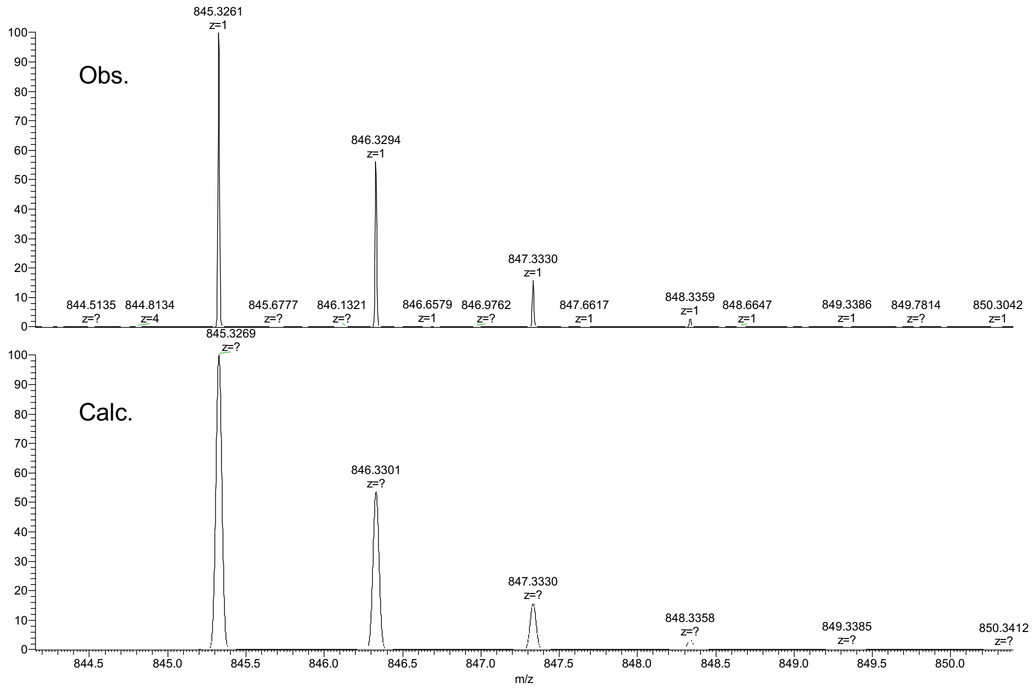

Spectrum 6. High-resolution ESI-MS isotopic distribution of the fragment **5**. Top: Measured isotopic distribution for $C_{47}H_{46}N_6O_8Na$ ($[M+Na]^+$, +ESI). Bottom: Simulated isotopic distribution for $C_{47}H_{46}N_6O_8Na$.

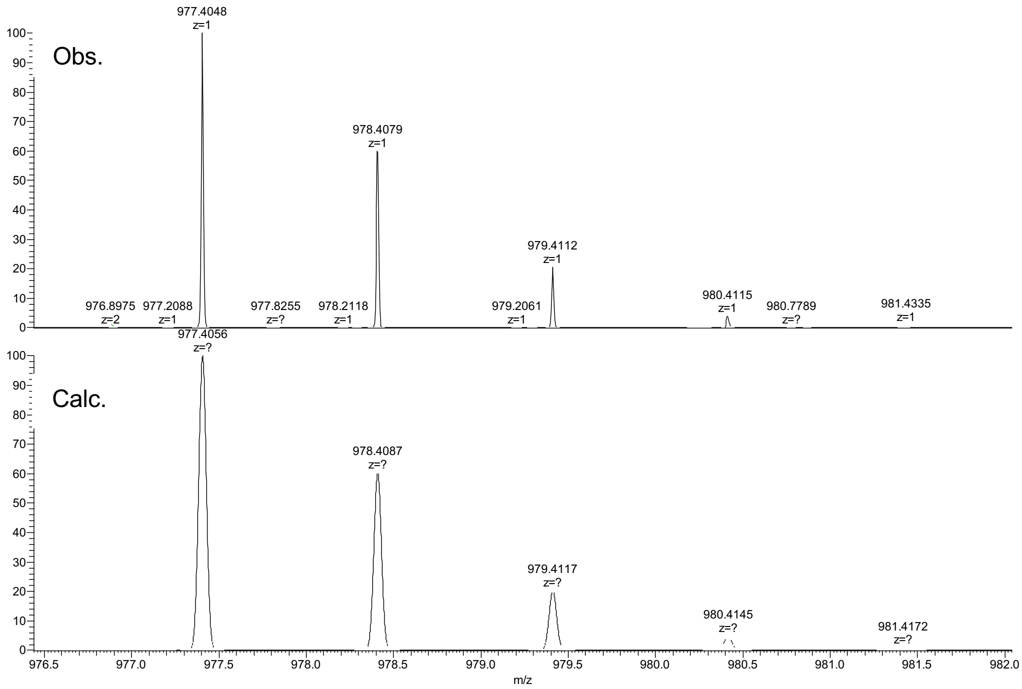

Spectrum 7. High-resolution ESI-MS isotopic distribution of the fragment **6**. Top: Measured isotopic distribution for $C_{53}H_{58}N_6O_{11}Na$ ($[M+Na]^+$, +ESI). Bottom: Simulated isotopic distribution for $C_{53}H_{58}N_6O_{11}Na$.

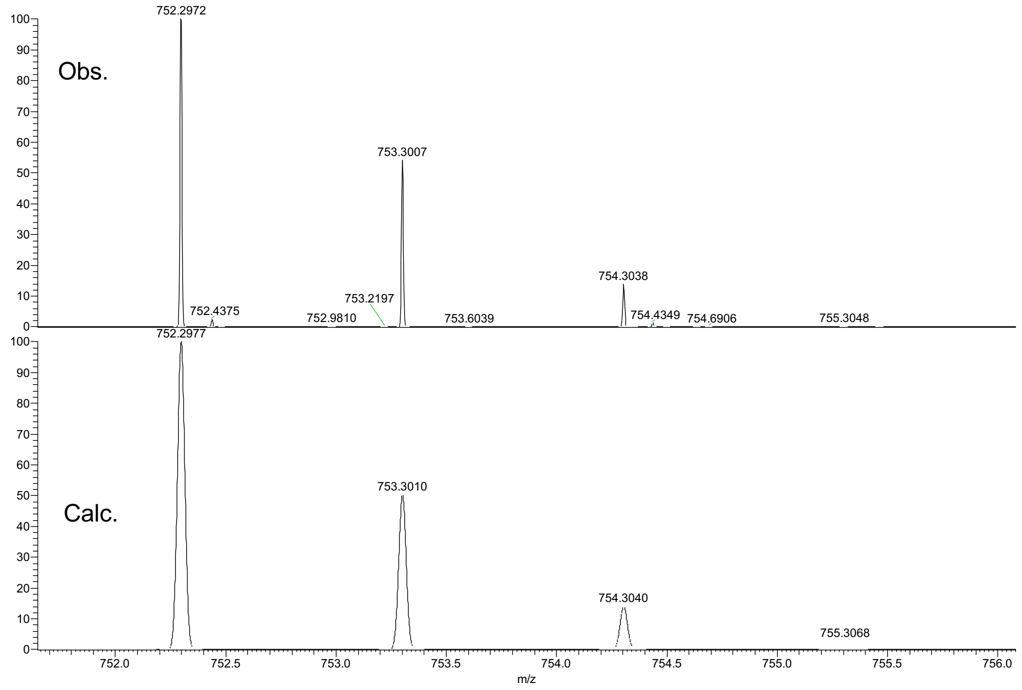

Spectrum 8. High-resolution ESI-MS isotopic distribution of the fragment **S25**. Top: Measured isotopic distribution for $C_{45}H_{42}N_3O_8$ ($[M-H]^-$, -ESI). Bottom: Simulated isotopic distribution for $C_{45}H_{42}N_3O_8$.

4.9 NMR spectra and MS spectra of the isolated fragments 4 and 6

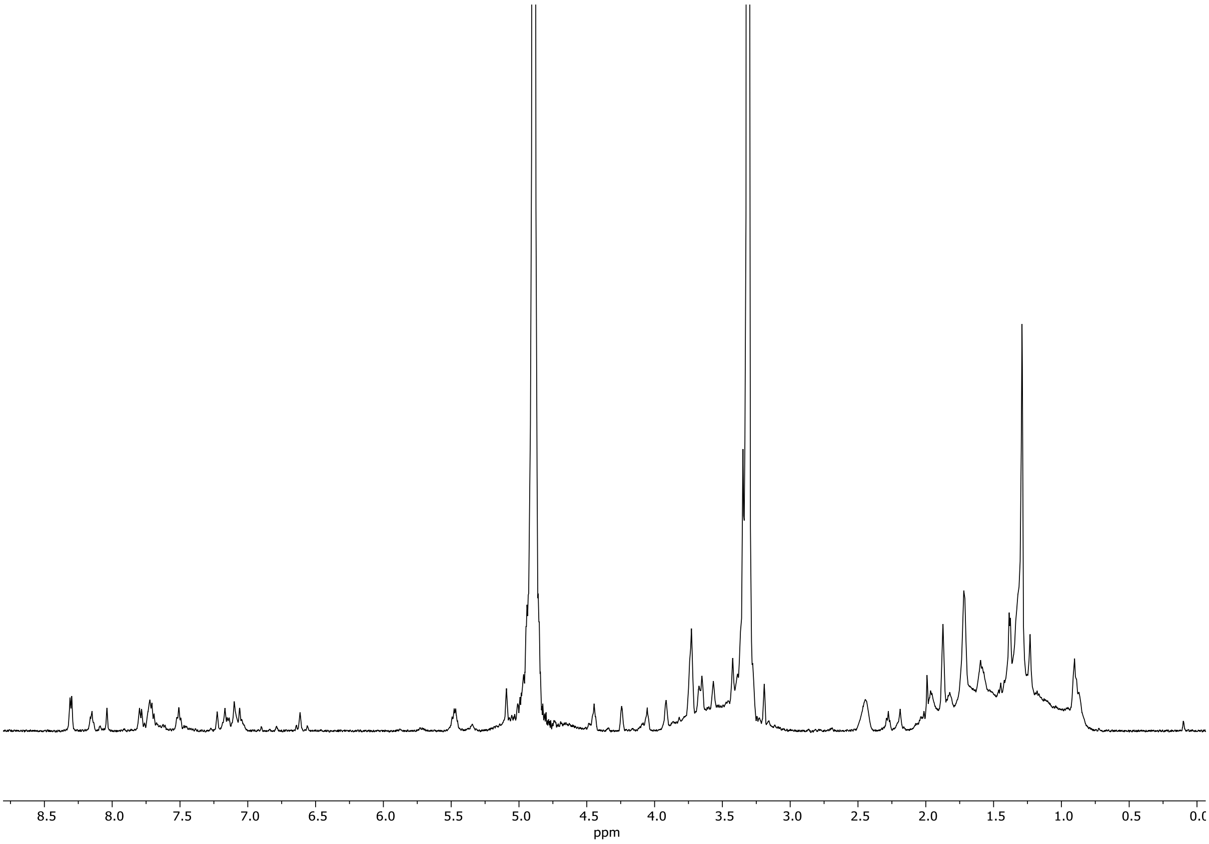

Spectrum 9. ^{1}H NMR spectrum (600 MHz, MeOD, 298 K) of the crude mixture after detachment from the post-sonication sample.

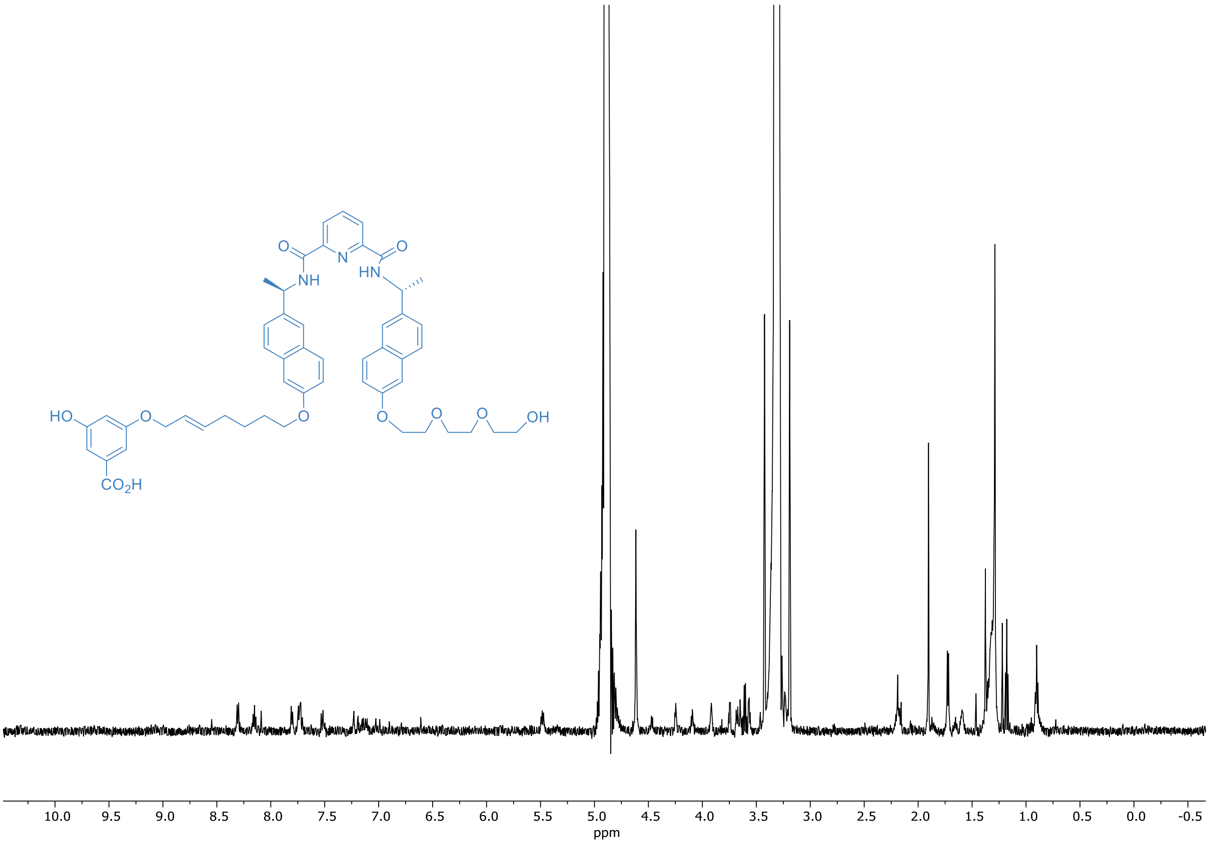

Spectrum 10. ^{1}H NMR spectrum (600 MHz, MeOD, 298 K) of isolated fragment **4**.

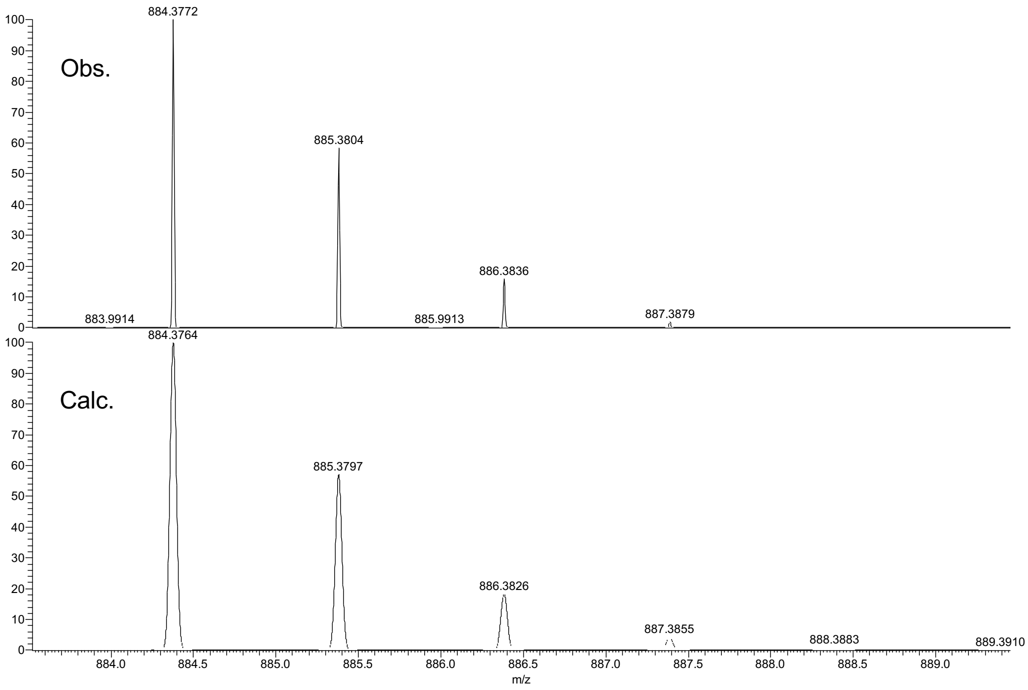

Spectrum 11. High-resolution ESI-MS isotopic distribution of the first fragment. Top: Measured isotopic distribution for $C_{51}H_{54}N_3O_{11}$ ([M-H]$^-$, -ESI). Bottom: Simulated isotopic distribution for $C_{51}H_{54}N_3O_{11}$.

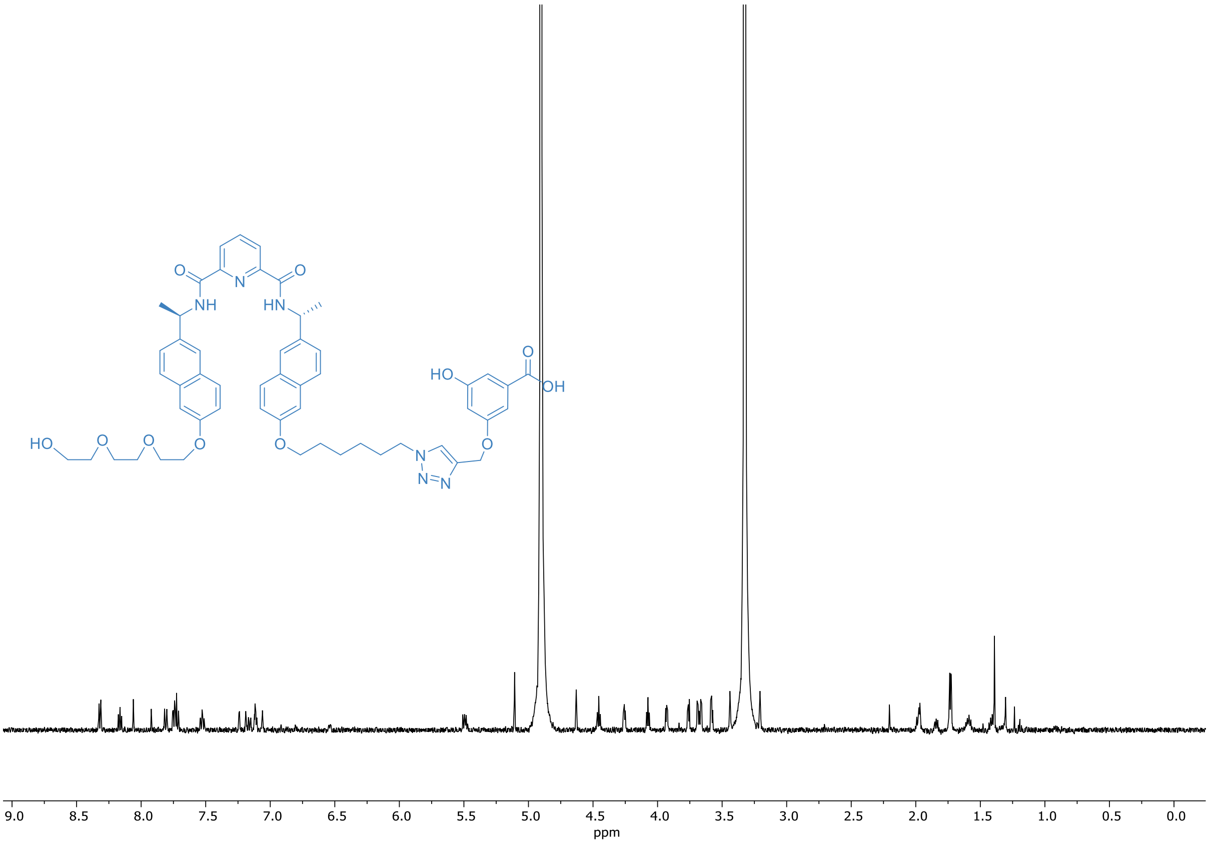

Spectrum 12. ^{1}H NMR (600 MHz, MeOD, 298 K) of isolated fragment **6**.

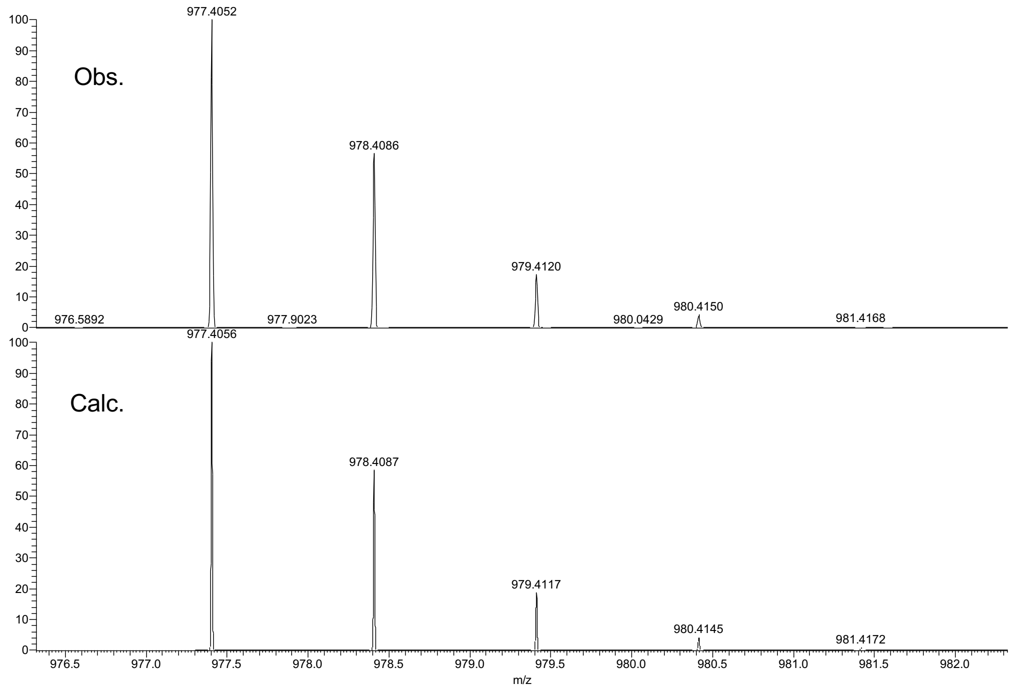

Spectrum 13. High-resolution ESI-MS isotopic distribution of the second fragment. Top: Measured isotopic distribution for $C_{53}H_{58}N_6O_{11}Na$ ($[M+Na]^+$, +ESI). Bottom: Simulated isotopic distribution for $C_{53}H_{58}N_6O_{11}Na$.

4.10 MS of sonicated solution before hydrolysis

To confirm the structures of the detached fragments in sections 4.7-4.9 originated from the hydrolysis of the sonicated sample, the MeOH-washed fraction from the sonicated polymer **1** in section 4.1 was analyzed by mass spectometry. MS analysis showed some low molecular weight species that could not be identified, but no fragments of the original ligand from prior to hydrolysis. This indicates that the detected fragments in sections 4.7-4.9 are not sonication byproducts. The region from 800-1000 m/z are expanded.

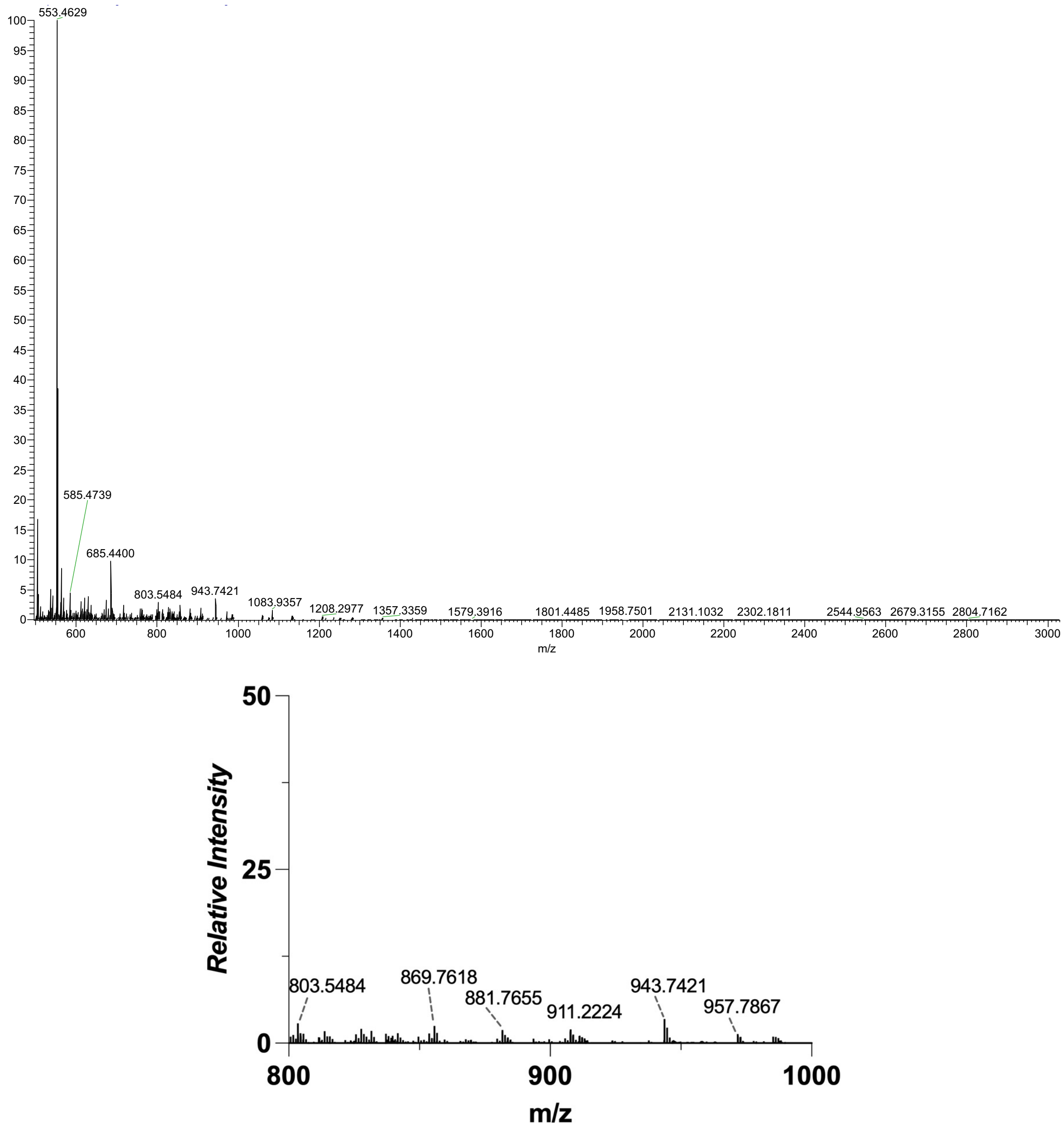

Spectrum 14. High-resolution ESI-MS(+) of the MeOH fraction of sonicated knot **1** from the process described in Section 4.1. Below: expansion from 800-1000 m/z.

4.11 Representative NMR spectra of sonicated polymers

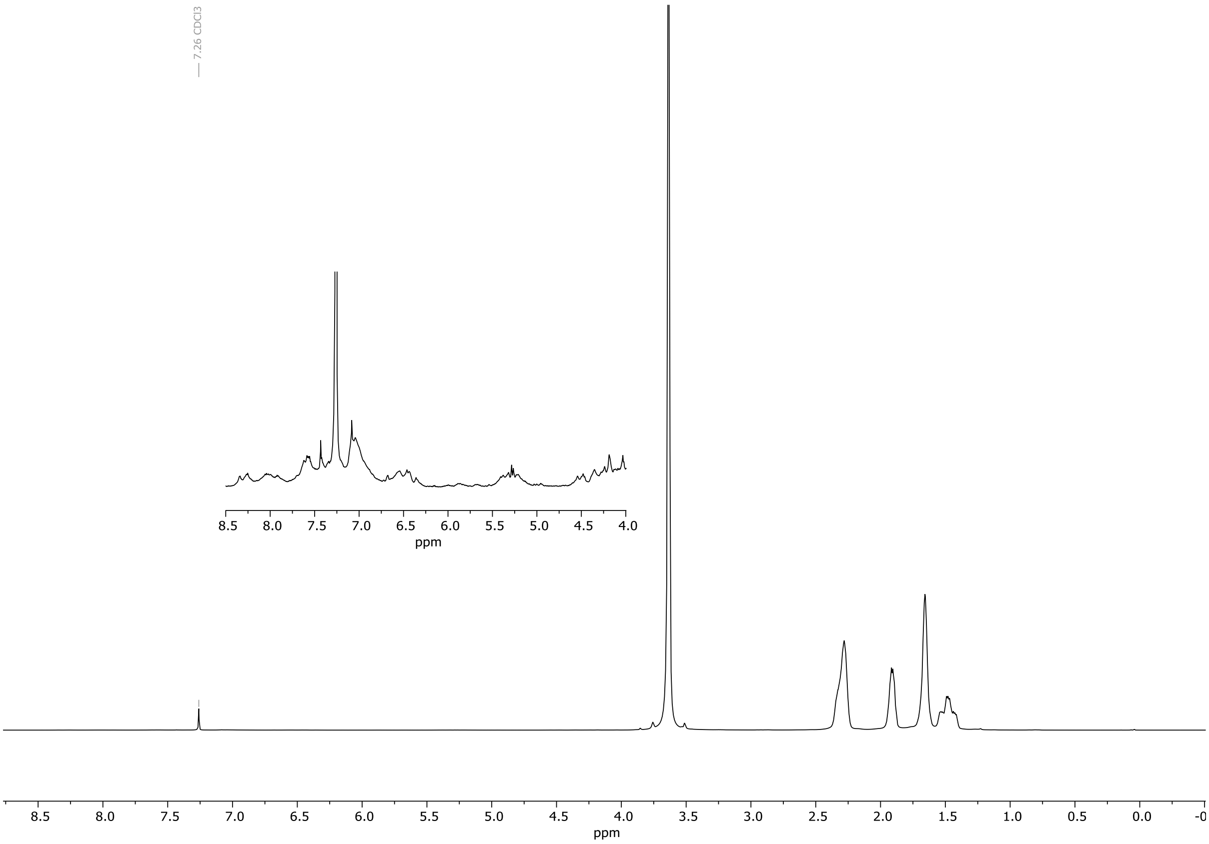

Spectrum 15. ^{1}H NMR spectrum (600 MHz, $CDCl_3$, 298 K) of gated organic knot polymer **1** after 180 min of sonication.

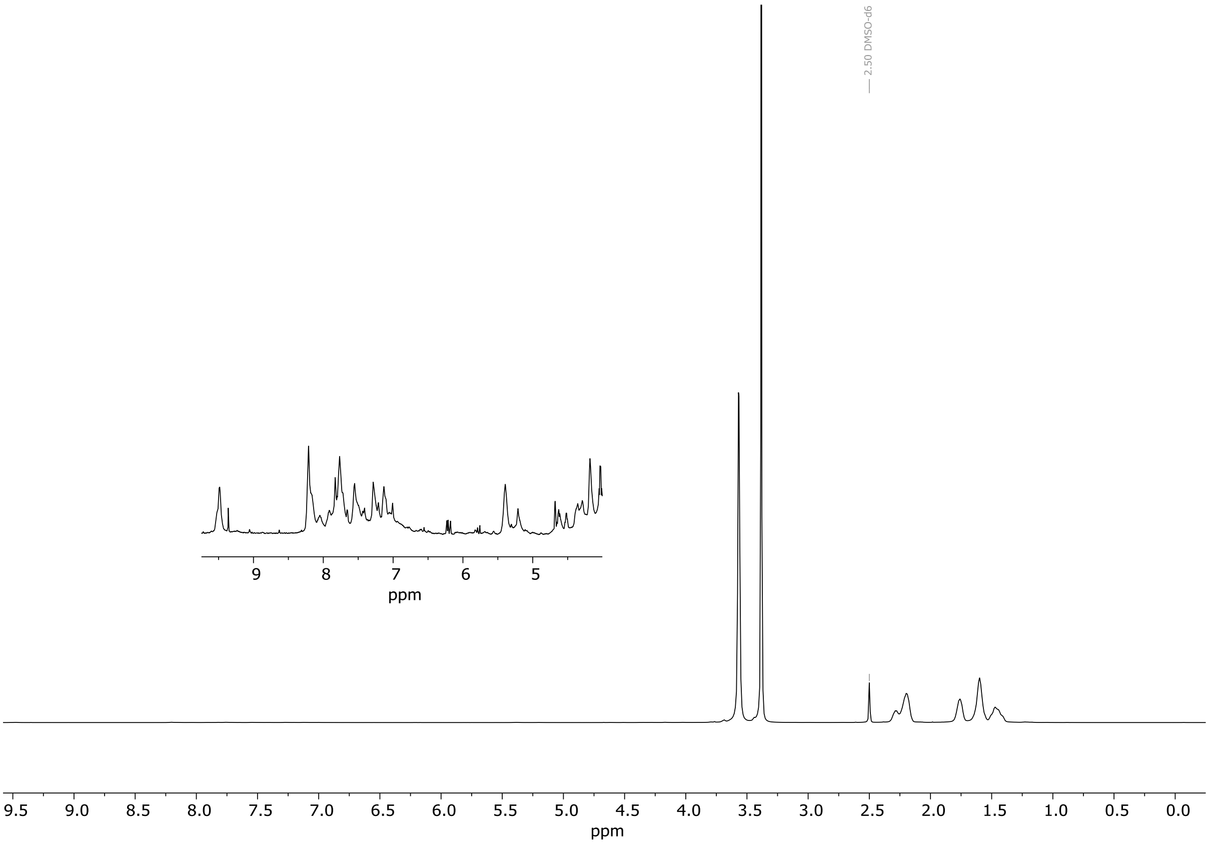

Spectrum 16. ^{1}H NMR spectrum (600 MHz, DMSO-d_6, 298 K) of gated organic knot polymer **1** after 180 min of sonication.

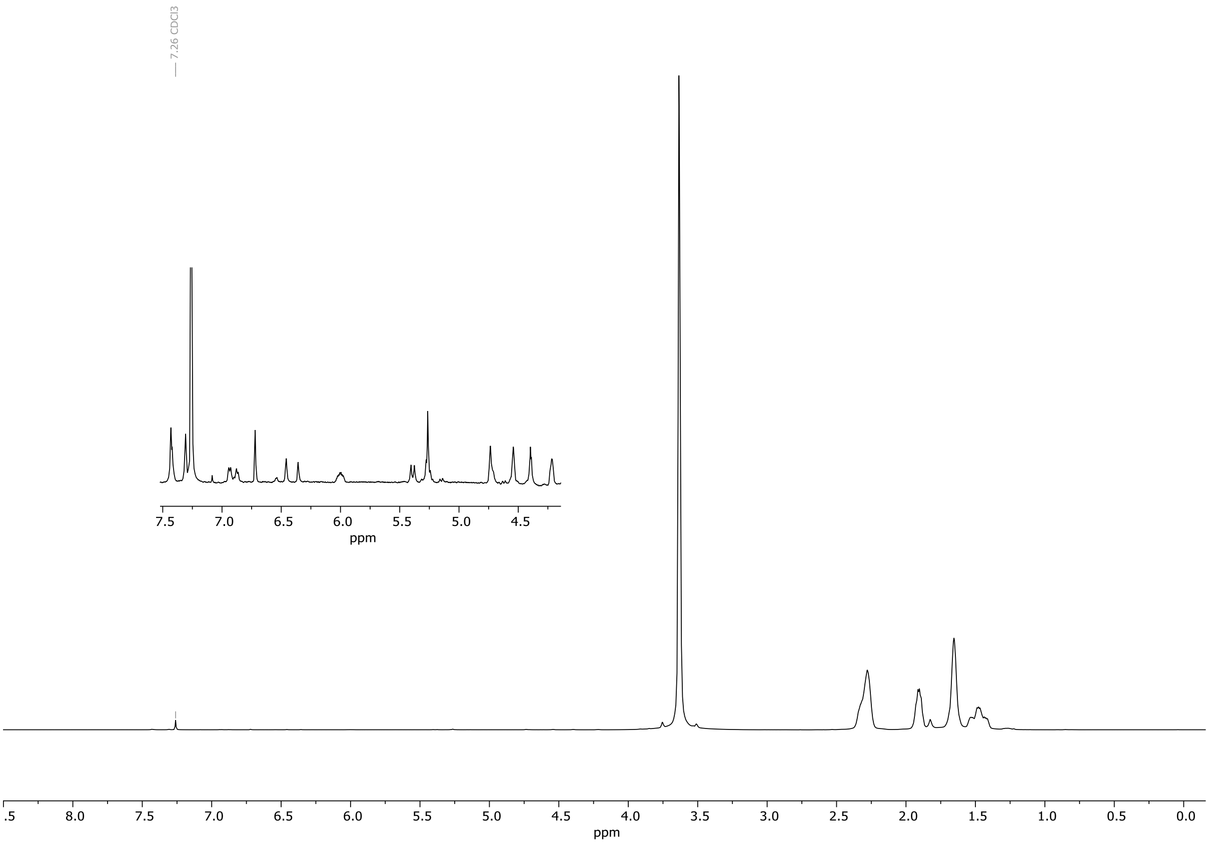

Spectrum 17. ^1H NMR spectrum (600 MHz, $CDCl_3$, 298 K) of gate polymer **2** after 180 min of sonication.

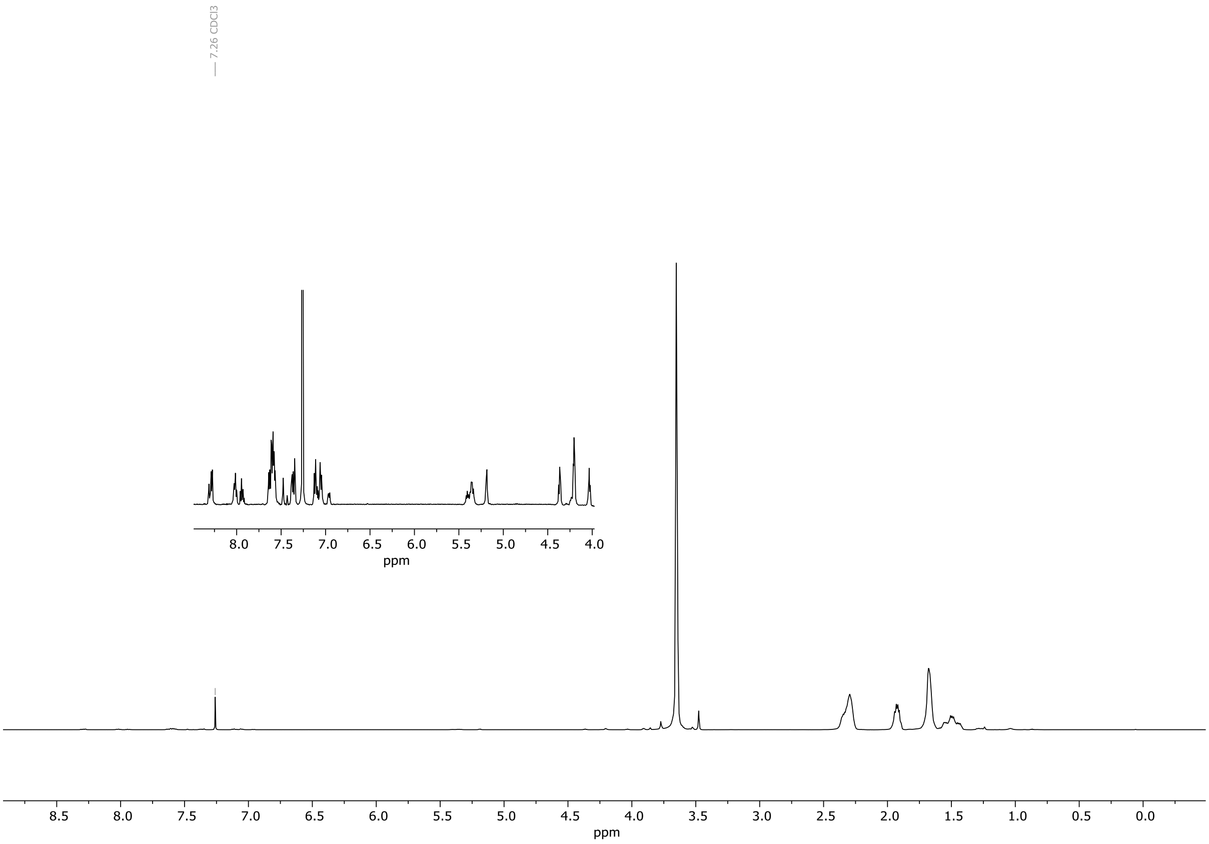

Spectrum 18. ^1H NMR spectrum (600 MHz, $CDCl_3$, 298 K) of linear ligand polymer **3** after 240 min of sonication.

5 Synthetic procedures

5.1 Synthetic procedures and characterisation details

S1

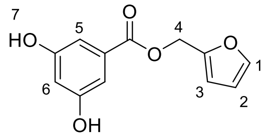

To a mixture of 3,5-dihydroxybenzoic acid (1.54 g, 10 mmol) and furfuryl alcohol (5.88 g, 60 mmol) was added EDCI (1.92 g, 10 mmol) and DMAP (0.122 g, 1 mmol). The reaction was stirred at 60 °C for 4 h. The crude mixture was cooled to RT and washed with hexane to remove the excess furfuryl alcohol. Then the mixture was washed with 1M HCl (50 mL) and extracted with DCM (3 x 50 mL). The organic phases were combined and washed with brine, dried with $MgSO_4$, filtered and concentrated. Further purification by flash column chromatography (PE/EtOAc 1:1) yielded compound **S1** (350 mg, 15%) as a colourless solid. **^{1}H NMR** (400 MHz, $(CD_3)_2CO$) δ 8.55 (br, 2H, H_7), 7.60-7.59 (m, 1H, H_1), 7.00 (d, *J* = 2.3 Hz, 2H, H_5), 6.59-6.56 (m, 2H, $H_{3,6}$), 6.46-6.44 (m, 1H, H_2), 5.27 (s, 2H, H_4); **^{13}C NMR** (101 MHz, $(CD_3)_2CO$) δ 166.30, 159.56, 150.87, 144.47, 144.45, 132.87, 111.56, 108.7, 108.2, 59.00. **HRMS** (ESI^-): Calcd. for $C_{12}H_9O_5^-$: 233.0455, found 233.0450 $[M-H]^-$.

S2

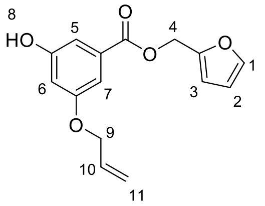

To a stirred solution of **S1** (350 mg, 1.5 mmol) and potassium carbonate (207.2 mg, 1.5 mmol) in degassed DMF (10 mL) was added 3-bromo-1-propene (181.5 mg, 1.5 mmol). The reaction was stirred for 4 hours at 80 °C. The mixture was allowed to cool to room temperature and concentrated under reduced pressure. Further purification by flash column chromatography (PE/EtOAc 2:1) yielded **S2** as a colorless oil (127 mg, 31%). **^{1}H NMR** (400 MHz, $CDCl_3$) δ 7.45-7.43 (m, 1H, H_1), 7.19-7.17 (m, 1H, H_7), 7.14-7.13 (m, 1H, H_5), 6.63-6.61 (m, 1H, H_6), 6.48 (d, *J* = 3.2 Hz, 1H, H_3), 6.39-6.37 (m, 1H, H_2), 6.03 (ddt, *J* = 17.3, 10.6, 5.3 Hz, 1H, H_{10}), 5.43 (dd, *J* = 17.3, 1.6 Hz, 1H, $H_{11trans}$), 5.30-5.28 (m, 3H, $H_{4,11cis}$), 4.53 (d, *J* = 5.3 Hz, 2H, H_9); **^{13}C NMR** (101 MHz, $CDCl_3$) δ 166.04, 159.96, 156.78, 149.48, 143.50, 132.85, 132.00, 118.17, 111.05, 110.75, 109.56, 108.40, 107.53, 69.22, 58.88; **HRMS** (ESI^-): Calcd. for $C_{15}H_{13}O_5^-$: 273.0768, found 273.0758 $[M-H]^-$.

S3

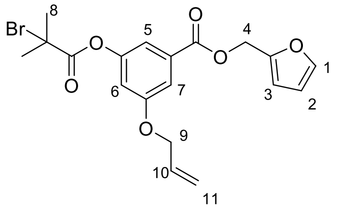

To a stirred solution of **S2** (120 mg, 0.44 mmol) and Et_3N (61 µL, 0.44 mmol) in THF (5 mL) in an ice bath was slowly added α-bromoisobutyryl bromide in dropwise (56 µL, 0.44 mmol). The reaction was stirred at 0 °C for 1 hour, after which the mixture was slowly warmed to RT. The mixture was washed with 1M HCl (5 mL) and extracted with DCM (3 x 10 mL). The organic phases were combined and washed with brine, dried with $MgSO_4$, filtered and concentrated. Further purification by flash column chromatography (PE/EtOAc 5:1) yielded **S3** as a light yellow oil (167 mg, 91%). **^{1}H NMR** (600 MHz, $CDCl_3$) δ 7.51-7.49 (m, 1H, H_7), 7.45-7.44 (m, 1H, H_1), 7.40-7.38 (m, 1H, H_5), 6.91-6.89 (m, 1H, H_6), 6.49 (d, *J* = 3.3 Hz, 1H, H_3), 6.40-6.37 (m, 1H, H_2), 6.03 (ddt, *J* = 17.2, 10.6, 5.3 Hz, 1H, H_{10}), 5.42 (dd, *J* = 17.3, 1.6 Hz, 1H, $H_{11trans}$), 5.33-5.28 (m, 3H, $H_{4,11cis}$), 4.57 (d, *J* = 5.3 Hz, 2H, H_9), 2.06 (s, 6H, H_8); **^{13}C NMR** (151 MHz, $CDCl_3$) δ 170.09, 165.28, 159.49, 151.54, 149.35, 143.55, 132.46,

132.15, 118.42, 115.13, 113.66, 113.29, 111.25, 110.78, 69.45, 58.96, 55.19, 30.72; **HRMS** (ESI$^+$): Calcd. for $C_{19}H_{20}O_6Br^+$: 423.0438, found 423.0451 $[M+H]^+$.

S5

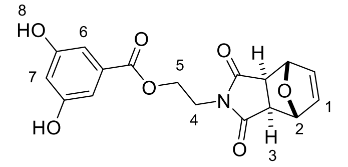

To a mixture of 3,5-dihydroxybenzoic acid (1.00 g, 6.49 mmol) and **S4** (5.40 g, 24.77 mmol) in DMF (7 mL) was added EDCI (1.24 g, 6.49 mmol) and DMAP (0.16 g, 1.31 mmol), and the reaction was stirred at 60 °C for 4 h. The crude mixture was cooled to RT, after which the mixture was washed with 1M HCl (20 mL) and extracted with DCM (3 x 50 mL). The organic phases were combined and washed with brine, dried with $MgSO_4$, filtered and concentrated. Further purification by flash column chromatography (3% MeOH in DCM) yielded compound **S5** (233 mg, 10%) as a colourless solid. ^{1}**H NMR** (400 MHz, $(CD_3)_2CO$) δ 8.60 (br, 2H, H_8), 6.96 (d, J = 2.3 Hz, 2H, H_6), 6.51 (app s, 3H, $H_{1,7}$), 5.13 (app s, 2H, H_2), 4.33 (t, J = 5.4 Hz, 2H, H_5), 3.82 (t, J = 5.4 Hz, 2H, H_4), 2.94 (s, 2H, H_3); ^{13}**C NMR** (101 MHz, $(CD_3)_2CO$) δ 176.98, 166.53, 159.34, 137.37, 132.88, 108.85, 108.00, 81.73, 62.02, 48.31, 38.04. **HRMS** (ESI$^+$): Calcd. for $C_{17}H_{16}O_7N^+$: 346.0921, found 346.0920 $[M+H]^+$.

S6

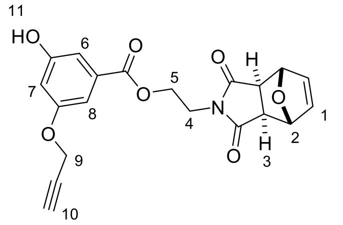

To a stirred solution of **S5** (230 mg, 0.66 mmol) and potassium carbonate (91.0 mg, 0.66 mmol) in degassed DMF (8 mL) was added propargyl bromide (78 mg, 0.66 mmol). The reaction was stirred for 4 hours at 80 °C. The mixture was allowed to cool to RT and concentrated under reduced pressure. Further purification by flash column chromatography (3% MeOH in DCM) yielded **S6** as a white solid (66 mg, 26%). ^{1}**H NMR** (400 MHz, $(CD_3)_2CO$) δ 8.88 (br, s, 1H, H_{11}), 7.11-7.09 (m, 2H, $H_{6,8}$), 6.71 (app t, J = 2.3 Hz, 1H, H_7), 6.56 (app s, 2H, H_1), 5.13 (app s, 2H, H_2), 4.81 (d, J = 2.4 Hz, 2H, H_9), 4.35 (t, J = 5.3 Hz, 2H, H_5), 3.84 (t, J = 5.3 Hz, 2H, H_4), 3.12 (t, J = 2.4 Hz, 1H, H_{10}), 2.95 (s, 2H, H_3); ^{13}**C NMR** (101 MHz, $(CD_3)_2CO$) δ 177.08, 166.21, 159.68, 159.31, 137.36, 132.89, 110.60, 107.86, 107.82, 81.72, 79.51, 77.26, 62.25, 56.43, 48.31, 38.03; **HRMS** (ESI$^+$): Calcd. for $C_{20}H_{18}O_7N^+$: 384.1078, found 384.1064 $[M+H]^+$.

S7

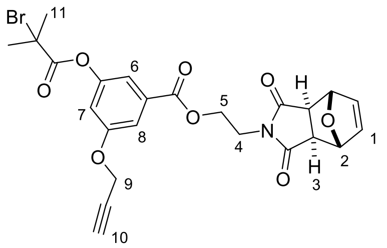

To a stirred solution of **S6** (60 mg, 0.16 mmol) and Et_3N (23 µL, 0.16 mmol) in THF (5 mL) in an ice bath was slowly added α-bromoisobutyryl bromide (22 µL, 0.16 mmol). The reaction was stirred for 1 hour, after which the mixture was allowed to warm to RT. The mixture was washed with 1M HCl (5 mL) and extracted with DCM (3 x 10 mL). The organic phase was combined and washed with brine, dried with $MgSO_4$, filtered and concentrated. Further purification by flash column chromatography (3% MeOH in DCM) yielded **S7** as a white solid (68 mg, 82%). ^{1}**H NMR** (400 MHz, $CDCl_3$) δ 7.51-7.50 (m, 1H, H_8), 7.40-7.39 (m, H_6), 6.96 (app t, J = 2.3 Hz, 1H, H_7), 6.49 (s, 2H, H_1), 5.25 (s, 2H, H_2), 4.75 (d, J = 2.4 Hz, 2H, H_9), 4.45 (t, J = 5.3 Hz, 2H, H_5), 3.89 (t, J = 5.3 Hz, 2H, H_4), 2.87 (s, 2H, H_3), 2.55 (t, J = 2.4 Hz, 1H, H_{10}), 2.06 (s, 6H, H_{11}); ^{13}**C NMR** (151 MHz, $CDCl_3$) δ 176.11, 169.95, 165.02, 158.36, 151.51, 136.65, 132.03, 115.95, 113.73,

113.45, 81.03, 77.83, 76.36, 61.62, 56.43, 55.20, 47.57, 37.79, 30.70; **HRMS** (APCI$^+$): Calcd. for $C_{24}H_{22}O_8NBr$: 531.0523, found 531.0537 $[M+H]^+$.

S8

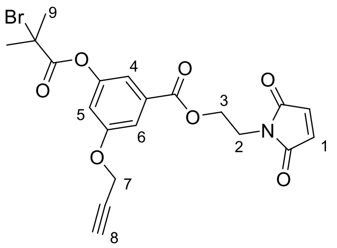

A stirred solution of **S7** (50 mg, 0.09 mmol) in toluene (5 mL) was heated at 105 °C for 16 h. The solvent was removed by high vacuum and the resulting product was used directly without further purification (43 mg). ^{1}**H NMR** (600 MHz, $CDCl_3$) δ 7.50-7.49 (m, 1H, H_6), 7.40-7.39 (m, 1H, H_4), 6.97 (app t, J = 2.3 Hz, 1H, H_5), 6.73 (s, 2H, H_1), 4.76 (d, J = 2.4 Hz, 2H, H_7), 4.43 (t, J = 5.3 Hz, 2H, H_3), 3.95 (t, J = 5.3 Hz, 2H, H_2), 2.55 (t, J = 2.4 Hz, 1H, H_8), 2.07 (s, 6H, H_9); ^{13}**C NMR** (151 MHz, $CDCl_3$) δ 170.53, 170.00, 165.14, 158.38, 151.56, 134.43, 131.89, 115.97, 113.92, 113.30, 77.81, 76.37, 62.78, 56.41, 55.18, 36.86, 30.71; **HRMS** (APCI$^+$): Calcd. for $C_{20}H_{19}O_7NBr$: 464.0339, found 464.0331 $[M+H]^+$.

S9

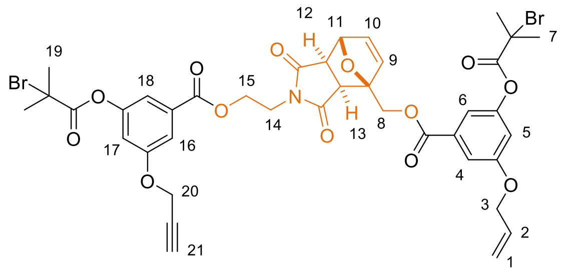

To a stirred solution of **S8** (43 mg, 0.09 mmol) in toluene (5 mL) was added **S3** (42.3 mg, 0.1 mmol), and the reaction mixture was heated at 75 °C for 48 h. The mixture was subsequently washed with 1M HCl (5 mL) and extracted with DCM (3 x 10 mL). The organic phases were combined and washed with brine, dried with $MgSO_4$, filtered and concentrated. Further purification by flash column chromatography (3% MeOH in DCM) yielded **S9** as a white solid (34 mg, 41%). ^{1}**H NMR** (400 MHz, $CDCl_3$) δ 7.51 (s, 1H, H_{16}), 7.46 (s, 1H, H_4), 7.39 (s, 1H, H_{18}), 7.35 (s, 1H, H_6), 6.95-6.93 (m, 1H, H_{17}), 6.91-6.89 (m, 1H, H_5), 6.55 (d, J = 5.5 Hz, 1H, H_{10}), 6.46 (d, J = 5.5 Hz, 1H, H_9), 6.01 (ddt, J = 15.9, 10.4, 5.1 Hz, 1H, H_2), 5.41(br d, J = 17.5 Hz, 1H, H_{1trans}), 5.29 (dd, J = 17.0 Hz, 1H, H_{1cis}), 5.26 (s, 2H, H_{11}), 5.17 (d, J = 12.9 Hz, 1H, H_8), 4.73-4.71 (m, 2H, H_{20}), 4.63 (d, J = 12.9 Hz, 1H, H_8), 4.57-4.54 (m, 2H, H_3), 4.50-4.46 (m, 1H, H_{15}), 4.43-4.39 (m, 1H, H_{15}), 3.95-3.85 (m, 2H, H_{14}), 3.02 (d, J = 6.6 Hz, 1H, H_{12}), 2.99 (d, J = 6.6 Hz, 1H, H_{13}), 2.53 (t, J = 2.3 Hz, 1H, H_{21}), 2.05 (s, 6H, $H_{7/19}$), 2.04 (s, 6H, $H_{7/19}$); ^{13}**C NMR** (101 MHz, $CDCl_3$) δ 175.51, 174.16, 170.02, 169.93, 165.05, 165.01, 159.46, 158.37, 151.56, 151.51, 137.82, 137.04, 132.40, 131.92, 131.76, 118.46, 115.92, 115.04, 113.91, 113.78, 113.39, 113.23, 89.82, 81.19, 81.14, 77.83, 77.81, 76.40, 75.34, 69.43, 62.21, 61.69, 56.38, 55.23, 55.21, 50.11, 48.61, 37.98, 30.67; **HRMS** (ESI$^+$): Calcd. for $C_{39}H_{37}O_{13}NBr_2Na$: 908.0524, found 908.0497 $[M+Na]^+$.

S11

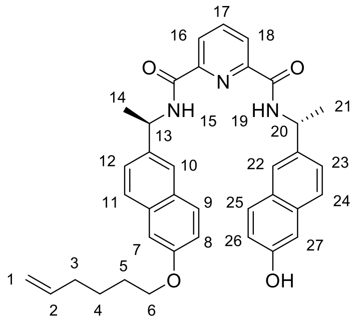

To a solution of **S10** (1.0 g, 1.98 mmol) and potassium carbonate (273 mg, 1.98 mmol) in degassed DMF (200 mL) was added 6-bromo-hex-1-ene (263 µL, 1.98 mmol). The reaction was stirred for 4 h at 80 °C, after which the mixture was concentrated under reduced pressure. Further purification by flash column chromatography (DCM/EtOAc 5:1) yielded compound **S11** (406 mg, 35%) as a colourless solid. **^{1}H NMR** (600 MHz, MeOD-d_4) δ 8.29 (d, J = 7.8 Hz, 2H, $H_{16,18}$), 8.13 (t, J = 7.8 Hz, 1H, H_{17}), 7.78 (s, 1H, H_{10}), 7.76 (s, 1H, H_{22}), 7.71 (d, J = 8.52 Hz, 1H, H_{25}), 7.70 (d, J = 5.9 Hz, 1H, H_9), 7.68 (d, J = 8.8 Hz, 1H, H_{11}), 7.62 (d, J = 8.6 Hz, 1H, H_{24}), 7.50 (dd, J = 8.6, 1.9 Hz, 1H, H_{12}), 7.46 (dd, J = 8.6, 1.9 Hz, 1H, H_{23}), 7.18 (s, 1H, H_7), 7.10 (dd, J = 9.0, 2.5 Hz, 1H, H_{26}), 7.08 (s, 1H, H_{27}), 7.05 (dd, J = 8.8, 2.4 Hz, 1H, H_8), 5.84 (ddt, J = 17.0, 10.2, 6.7 Hz, 1H, H_2), 5.46 (quint, J = 7.0 Hz, 2H, $H_{13,20}$), 5.04 (dd, J = 17.1, 1.9 Hz, 1H, H_{1trans}), 4.97-5.94 (m, 1H, H_{1cis}), 4.08 (t, J = 6.4 Hz, 2H, H_6), 2.16 (q, J = 7.2 Hz, 2H, H_3), 1.87-1.82 (m, 2H, H_5), 1.71 (d, J = 7.1 Hz, 3H, $H_{14/21}$), 1.70 (d, J = 7.1 Hz, 3H, $H_{14/21}$), 1.62 (quint, J = 7.4 Hz, 2H, H_4); **^{13}C NMR** (151 MHz, MeOD-d_4) δ 165.36, 165.34, 158.55, 156.53, 150.72, 150.70, 140.33, 139.78, 139.72, 139.03, 135.63, 135.37, 130.45, 130.30, 130.20, 129.66, 128.25, 127.67, 126.30, 126.24, 126.22, 125.43, 125.36, 120.19, 119.54, 115.16, 109.78, 107.39, 68.82, 50.26, 50.24, 34.61, 29.85, 26.62, 21.69, 21.65; **HRMS** (ESI$^+$): Calcd. for $C_{37}H_{37}O_4N_3Na$: 610.2676, found 610.2658 $[M+Na]^+$.

S12

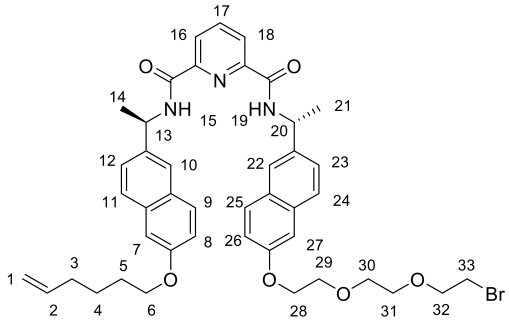

To a solution of **S11** (300 mg, 0.51 mmol) and potassium carbonate (282 mg, 2.04 mmol) in degassed DMF (8 mL) was added 1,2-bis(2-bromoethoxy)ethane (335 µL, 2.04 mmol). The reaction was stirred for 16 h at RT, after which the mixture was concentrated under reduced pressure. Further purification by flash column chromatography (DCM to DCM/EtOAc, 3:1) yielded compound **S12** (294 mg, 75%) as a colourless solid. **^{1}H NMR** (600 MHz, $CDCl_3$) δ 8.36 (d, J = 7.8 Hz, 2H, $H_{16,18}$), 8.03 (t, J = 7.8 Hz, 1H, H_{17}), 7.87 (d, J = 7.9 Hz, 2H, $H_{15,19}$), 7.72 (s, 2H, $H_{10,22}$), 7.67 (d, J = 9.0 Hz, 2H, $H_{9,25}$), 7.64 (d, J = 8.6 Hz, 1H, $H_{11/24}$), 7.63 (d, J = 8.6 Hz, 1H, $H_{11/24}$), 7.43 (d, J = 8.3 Hz, 2H, $H_{12,23}$), 7.19 (dd, J = 8.9, 2.6 Hz, 1H, $H_{8/26}$), 7.16 (dd, J = 8.8, 2.5 Hz, 1H, $H_{8/26}$), 7.09-7.12 (m, 2H, $H_{7,27}$), 5.85 (ddt, J = 16.9, 10.2, 6.7 Hz, 1H, H_2), 5.46-5.42 (m, 2H, $H_{13,20}$), 5.05 (dd, J = 17.1, 1.9 Hz, 1H, H_{1trans}), 4.96 (d, J = 10.1 Hz, 1H, H_{1cis}), 4.26 (t, J = 4.8 Hz, 2H, H_{28}), 4.09 (t, J = 6.5 Hz, 2H, H_6), 3.95 (t, J = 6.5 Hz, 2H, H_{29}), 3.82 (t, J = 6.5 Hz, 2H, H_{32}), 3.78-3.76 (m, 2H, H_{30}), 3.73-3.71 (m, 2H, H_{31}), 3.47 (t, J = 6.3 Hz, 2H, H_{33}), 2.16 (q, J = 7.2 Hz, 2H, H_3), 1.87 (quint, J = 6.7 Hz, 2H, H_5), 1.66-1.64 (m, 6H, $H_{14,21}$), 1.62 (quint, J = 7.6 Hz, 2H, H_4); **^{13}C NMR** (151 MHz, $CDCl_3$) δ 162.69, 157.45, 157.12, 148.95, 148.93, 139.23, 138.66, 138.03, 137.81, 134.14, 134.01, 129.50, 129.44, 128.98, 128.82, 127.70, 127.62, 125.31, 125.03, 125.01, 124.71, 119.69, 114.96, 106.72, 106.53, 71.40, 71.01, 70.75, 69.96, 67.98, 67.60, 49.18, 49.17, 33.61, 30.48, 28.82, 25.52, 21.78, 21.73; Due to the pseudo-symmetry, several ^{13}C signals are not resolved due to overlapping peaks. **HRMS** (ESI$^-$): Calcd. for $C_{44}H_{49}O_8N_3Br$: 826.2709, found 826.2742 $[M+HCO_2]^-$.

S13

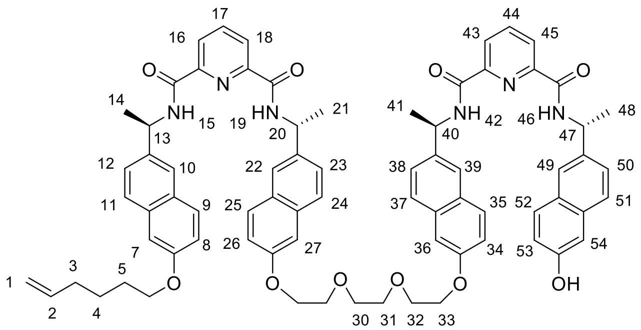

To a solution of **S12** (290 mg, 0.37 mmol) and potassium carbonate (51 mg, 0.37 mmol) in degassed DMF (50 mL) was added **S10** (187 mg, 0.37 mmol). The reaction was stirred for 16 h at 80 °C, after which the mixture was concentrated under reduced pressure. Further purification by flash column chromatography (EtOAc) yielded compound **S13** (100 mg, 22%) as a colourless solid. **^{1}H NMR** (600 MHz, $CDCl_3$) δ 8.35-8.32 (m, 4H, $H_{16,18,43,45}$), 8.01-7.96 (m, 4H, $H_{15,17,19,44}$), 7.86-7.84 (m, 2H, $H_{42,46}$), 7.68-7.61 (m, 4H, $H_{10,22,39,49}$), 7.60-7.56 (m, 4H, $H_{9,11,24,25}$), 7.53 (d, J =9.0 Hz, 1H, H_{37}), 7.50 (d, J =8.8 Hz, 1H, H_{51}), 7.45 (d, J = 8.5 Hz, 1H, H_{35}), 7.39 (dd, J = 9.0, 8.5 Hz, 2H, $H_{12,38}$), 7.31 (d, J = 8.4 Hz, 1H, H_{52}), 7.24 (d, J = 9.1 Hz, 1H, H_{23}), 7.19 (d, J = 9.1 Hz, 1H, H_{50}), 7.14-7.10 (m, 2H, $H_{8,26}$), 7.08-7.05 (m, 2H, $H_{34,53}$), 7.04-6.99 (m, 4H, $H_{7,27,36,54}$), 5.84 (ddt, J = 16.9, 10.1, 6.6 Hz, 1H, H_2), 5.44-5.37 (m, 2H, $H_{13/20/40/47}$), 5.33-5.28 (m, 2H, $H_{13/20/40/47}$), 5.05 (d, J = 17.0 Hz, 1H, H_{1trans}), 4.98 (d, J = 10.1 Hz, 1H, H_{1cis}), 4.26 (t, J = 4.8 Hz, 2H, H_{28}), 4.20 (5.05 (t, J = 4.7 Hz, 2H, H_{33}), 4.06 (t, J = 4.8 Hz, 2H, H_6), 4.01-3.96 (m, 2H , H_{29}), 3.95-3.91 (m, 2H, H_{32}), 3.84 (s, 4H, $H_{30,31}$), 2.17-2.13 (m, 2H, H_3), 1.86 (quint, J = 6.6 Hz, 2H, H_5), 1.64-1.58 (m, 12H, $H_{14,21,41,48}$), 1.54-1.52 (m, 2H, H_4); **^{13}C NMR** (151 MHz, $CDCl_3$) δ 162.95, 162.92, 162.84, 162.80, 157.40, 157.00, 156.95, 154.54, 149.07, 149.03, 148.91, 148.87, 139.21, 139.16, 138.64, 138. 137.79, 137.63, 136.92, 134.13, 134.09, 133.96, 133.94, 129.58, 129.46, 129.40, 128.94, 128.78, 128.41, 127.72, 127.66, 127.59, 127.23, 125.34, 125.32, 125.22, 125.18, 125.08, 125.07, 125.03, 124.91, 124.75, 124.69, 124.66, 124.53, 119.66, 119.53, 119.49, 118.85, 114.96, 109.51, 107.01, 106.85, 106.51, 71.09, 70.99, 70.02, 69.96, 67.97, 67.50, 67.43, 49.61, 49.53, 49.13, 33.60, 28.80, 25.51, 21.65, 21.58, 21.31; Due to the pseudo-symmetry, several ^{13}C signals are not resolved due to overlapping peaks. **HRMS** (ESI$^+$): Calcd. for $C_{74}H_{74}O_{10}N_6Na$: 1229.5359, found 1229.5327 $[M+M]^+$.

S14

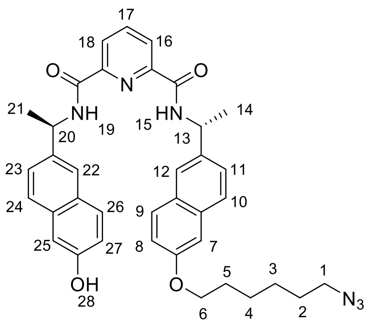

To a solution of **S10** (500 mg, 0.99 mmol) and potassium carbonate (136 mg, 0.99 mmol) in degassed DMF (100 mL) was added 1-azido-6-bromohexane (204 mg, 0.99 mmol). The reaction was stirred for 4 h at 80 °C. The mixture was concentrated under reduced pressure. Further purification by flash column chromatography (DCM/EtOAc 5:1) yielded compound **S14** (130 mg, 21%) as a colourless solid. **^{1}H NMR** (600 MHz, $CDCl_3$) δ 8.34 (d, J = 7.8 Hz, 2H, $H_{16,18}$), 7.98 (t, J = 7.8 Hz, 1H, H_{17}), 7.68 (s, 1H, H_{12}), 7.64 (s, 1H, H_{22}), 7.63-7.61 (m, 2H, $H_{9,26}$), 7.55 (d, J = 9.5 Hz, 1H, H_{10}), 7.48 (d, J = 8.5 Hz, 1H, H_{24}), 7.41 (dd, J = 8.5, 1.8 Hz, 1H, H_{11}), 7.35 (dd, J = 8.5, 1.9 Hz, 1H, H_{23}), 7.14 (d, J = 2.5 Hz, 1H, H_{27}), 7.08 (s, 1H, H_7), 7.05-7.03 (m, 2H, $H_{8,25}$), 6.20 (br, 1H, H_{28}), 5.44-5.39 (m, 2H, $H_{13,20}$), 4.07 (t, J = 6.5 Hz, 2H, H_6), 3.28 (t, J = 6.5 Hz, 2H, H_1), 1.86 (quint, J = 6.7 Hz, 2H, H_5), 1.67 (quint, J = 7.2 Hz, 2H, H_2), 1.61 (d, J = 6.9 Hz, 3H, $H_{14/21}$), 1.60 (d, J = 6.9 Hz, 3H, $H_{14/21}$), 1.54 (quint, J = 7.0 Hz, 2H, H_4), 1.48 (quint, J = 7.0 Hz, 2H, H_3); **^{13}C NMR** (151 MHz, $CDCl_3$) δ 162.92, 162.86, 162.80, 157.38, 154.13, 148.93, 148.85, 139.24, 137.75, 137.56, 134.11, 129.72, 129.44, 128.83, 128.64, 127.61, 127.23, 125.37, 125.01, 124.95, 124.76, 124.71, 119.64, 118.54, 109.48,

106.61, 67.95, 51.53, 49.24, 49.21, 49.13, 49.10, 29.22, 29.94, 26.66, 25.88, 21.71; **HRMS** (ESI$^+$): Calcd. for $C_{37}H_{38}O_4N_6Na$: 653.2847, found 653.2829 $[M+Na]^+$.

S15

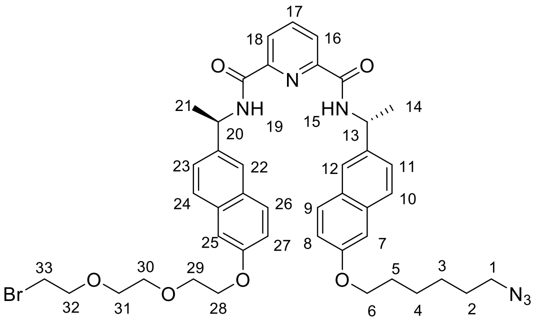

To a solution of **S14** (120 mg, 0.19 mmol) and potassium carbonate (105 mg, 0.76 mmol) in degassed DMF (5 mL) was added 1,2-bis(2-bromoethoxy)ethane (125 µL, 0.76 mmol). The reaction was stirred for 16 h at RT, after which the mixture was concentrated under reduced pressure. Further purification by flash column chromatography (DCM to DCM/EtOAc, 3:1) yielded compound **S15** (120 mg, 76%) as a colourless solid. **^{1}H NMR** (600 MHz, $CDCl_3$) δ 8.36 (d, *J* = 7.8 Hz, 2H, $H_{16,18}$), 8.03 (t, *J* = 7.8 Hz, 1H, H_{17}), 7.89 (d, *J* = 7.6 Hz, 1H, $H_{15,19}$), 7.72 (s, 2H, $H_{12,22}$), 7.67 (d, *J* = 9.0 Hz, 2H, $H_{9,26}$), 7.66-7.63 (m, 2H, $H_{10,24}$), 7.43 (dd, *J* = 8.5, 1.8 Hz, 1H, $H_{11,23}$), 7.19 (dd, *J* = 8.9, 2.5 Hz, 1H, $H_{8/27}$), 7.17 (dd, *J* = 8.9, 2.5 Hz, 1H, $H_{8/27}$), 7.11 (d, *J* = 7.6 Hz, 2H, $H_{7,25}$), 5.46-5.41 (m, 2H, $H_{13,20}$), 4.26 (t, *J* = 4.8 Hz, 2H, H_{28}), 4.08 (t, *J* = 6.4 Hz, 2H, H_6), 3.95 (t, *J* = 4.7 Hz, 2H, H_{29}), 3.82 (t, *J* = 6.3 Hz, 2H, H_{32}), 3.78-3.76 (m, 2H, H_{30}), 3.73-3.71 (m, 2H, H_{31}), 3.47 (t, *J* = 6.3 Hz, 2H, H_{33}), 3.29 (t, *J* = 6.9 Hz, 2H, H_1), 1.87 (quint, *J* = 6.7 Hz, 2H, H_5), 1.66 (d, *J* = 6.9 Hz, 3H, $H_{14/21}$), 1.65 (d, *J* = 6.9 Hz, 3H, $H_{14/21}$), 1.64-1.60 (m, 2H, H_2), 1.55 (quint, *J* = 7.0 Hz, 2H, H_4), 1.48 (quint, *J* = 6.8 Hz, 2H, H_3); **^{13}C NMR** (151 MHz, $CDCl_3$) δ 162.70, 157.40, 157.11, 148.93, 148.92, 139.23, 128.05, 137.86, 134.12, 134.00, 129.50, 129.47, 128.98, 129.84, 127.70, 127.62, 125.31, 125.04, 124.71, 119.69, 119.65, 105.73, 105.55, 71.39, 71.01, 70.74, 69.96, 67.92, 67.59, 51.52, 49.17, 30.48, 29.85, 29.25, 28.95, 26.69, 25.90, 21.79, 21.73; Due to the pseudo-symmetry, several ^{13}C signals are not resolved due to overlapping peaks. **HRMS** (ESI$^-$): Calcd. for $C_{43}H_{49}O_6N_6BrCl$: 859.2613, found 859.2580 $[M+Cl]^-$.

S16

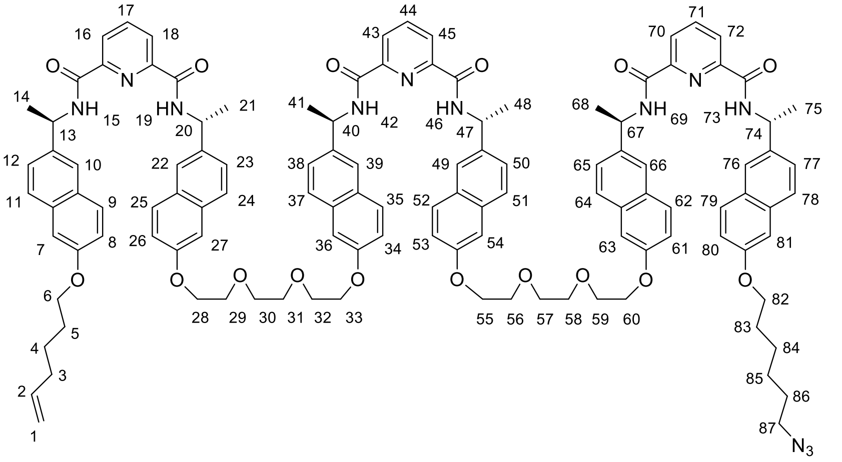

To a solution of **S13** (80 mg, 0.066 mmol) and potassium carbonate (27.5 mg, 0.198 mmol) in degassed DMF (4 mL) was added **S15** (55 mg, 0.066 mmol). The reaction was stirred for 16 h at 80 °C, after which the mixture was concentrated under reduced pressure. Further purification by flash column chromatography (EtOAc to DCM/MeOH, 10:1) yielded compound **S16** (103 mg, 80%) as a colourless solid. **^{1}H NMR** (600 MHz, $CDCl_3$) δ 8.32-8.28 (m, 6H, $H_{16,18,43,45,70,72}$), 8.01-7.98 (m, 6H, $H_{15,19,42,46,69,73}$), 7.97-7.91 (m, 3H, $H_{17,44,71}$), 7.65 (s, 6H, $H_{10,22,39,49,66,76}$), 7.63-7.58 (m, 12H, $H_{9,11,24,25,35,37,51,52,62,64,78,79}$), 7.40-7.36 (m, 6H, $H_{12,23,38,50,65,77}$), 7.15-7.12 (m, 6H, $H_{8,26,34,53,61,80}$), 7.09-7.06 (m, 6H, $H_{7,27,36,54,63,81}$), 5.83 (ddt, *J* = 17.0, 10.2, 6.7 Hz, 1H, H_2), 5.42-5.35 (m, 6H, $H_{13,20,40,47,67,74}$), 5.05-5.03 (m, 1H, H_{1trans}), 4.99-4.97 (m, 1H, H_{1cis}), 4.23-4.21 (m, 8H, $H_{28,33,55,60}$), 4.08-4.05 (m,

4H, $H_{6,82}$), 3.93-3.91 (m, 8H, $H_{29,32,56,59}$), 3.79 (s, 8H, $H_{30,31,57,58}$), 3.28 (t, J = 6.9 Hz, 2H, H_{87}), 2.15 (q, J = 6.4 Hz, 2H, H_3), 1.86 (quint, J = 8.0 Hz, 4H, $H_{5,83}$), 1.64 (quint, J = 7.9 Hz, 4H, $H_{4,84}$), 1.60-1.57 (m, 20H, $H_{14,21,41,48,68,75,86}$), 1.48 (quint, J = 6.8 Hz, 2H, H_{85}); **^{13}C NMR** (151 MHz, $CDCl_3$) δ 162.85, 162.83, 162.69, 157.35, 157.30, 157.04, 148.83, 138.99, 138.62, 138.07 138.05, 137.90, 137.85, 134.00, 133.98, 133.89, 133.87, 129.38, 129.37, 129.35, 129.32, 128.86, 128.84, 128.72, 128.70, 127.55, 127.53, 127.47, 125.21, 125.17, 125.14, 125.11, 124.55, 119.60, 119.58, 119.56, 114.93, 106.71, 106.47, 106.45, 71.05, 69.91, 67.92, 67.87, 67.56, 51.48, 48.91, 36.59, 33.58, 31.53, 29.82, 29.22, 28.92, 28.79, 26.65, 25.86, 25.49, 21.54, 21.48, 21.44, 21.38; *Due to the high degree of apparent symmetry within **S16** many ^{13}C signals overlap. **HRMS** (ESI$^+$): Calcd. for $C_{117}H_{122}O_{16}N_{12}Na$: 1973.8991, found 1973.8994 [M+Na]$^+$.

S18

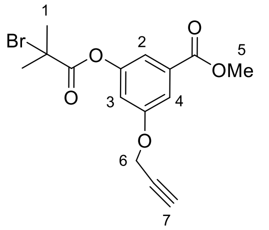

To a stirred solution of **S17** (206 mg, 1.0 mmol) and Et_3N (139 µL, 1.0 mmol) in THF (10 mL) at 0 °C was dropwise added α-bromoisobutyryl bromide (127 µL, 1.0 mmol). The reaction was stirred for 1 hour, after which the reaction mixture was allowed to warm to RT. The mixture was then washed with 1M HCl (10 mL) and extracted with DCM (3 x 10 mL). The organic phases were combined and washed with brine, dried with $MgSO_4$, filtered and concentrated. Further purification by flash column chromatography (PE/EtOAc 4:1) yielded **S18** as a light yellow oil (308 mg, 87%). **^{1}H NMR** (600 MHz, $CDCl_3$) δ 7.56 (dd, J = 2.5, 1.3 Hz, 1H, H_4), 7.45 (dd, J = 2.2, 1.3 Hz, 1H, H_2), 6.97 (t, J = 2.2 Hz, 1H, H_3), 4.75 (d, J = 2.4 Hz, 3H, H_6), 3.92 (s, 3H, H_5), 2.56 (t, J = 2.4 Hz, 1H, H_7), 2.07 (s, 6H, H_1); **^{13}C NMR** (151 MHz, $CDCl_3$) δ 170.05, 165.96, 158.40, 151.54, 132.47, 115.79, 113.51, 113.46, 77.71, 76.46, 56.46, 55.16, 52.65, 30.71. **HRMS** (ESI$^+$): Calcd. for $C_{15}H_{15}O_5BrNa$: 376.9995, found 376.9994 [M+Na]$^+$.

S19

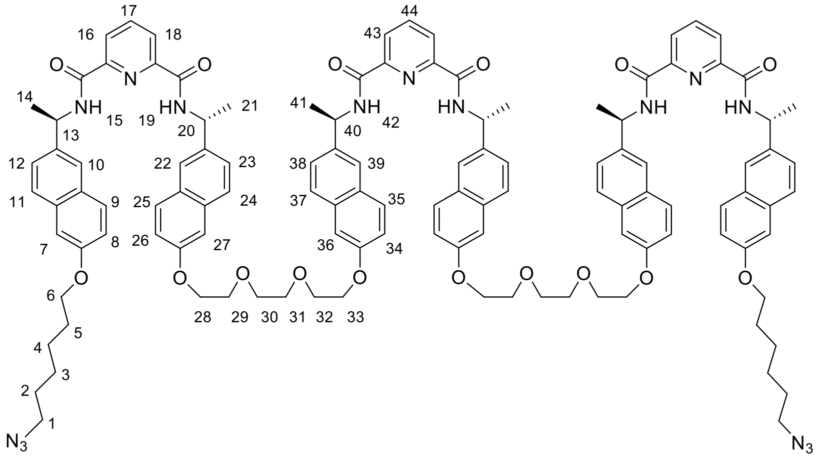

To a solution of **S15** (50 mg, 0.061 mmol) and potassium carbonate (33 mg, 0.24 mmol) in degassed DMF (4 mL) was added **S10** (15 mg, 0.03 mmol). The reaction was stirred for 16 h at 80 °C, after which the mixture was concentrated under reduced pressure. Further purification by size exclusion chromatography (DCM as eluent) yielded compound **S19** (48 mg, 80%) as a colourless solid. **^{1}H NMR** (600 MHz, $CDCl_3$) δ 8.32-8.28 (m, 6H, $H_{16,18,43}$), 8.01-7.98 (m, 6H, $H_{15,19,42}$), 7.96-7.92 (m, 3H, $H_{17,44}$), 7.65-7.58 (m, 18H, $H_{9,10,11,22,24,25,35,37,39}$), 7.40-7.36 (m, 6H, $H_{12,23,38}$), 7.14-7.12 (m, 6H, $H_{8,26,34}$), 7.09-7.06 (m, 6H, $H_{7,27,36}$), 5.43-5.35 (m, 6H, $H_{13,20,40}$), 4.22 (t, J = 4.8 Hz, 8H, $H_{28,33}$), 4.06 (t, J = 6.4 Hz, 4H, H_6), 3.92 (t, J = 4.8 Hz, 8H, $H_{29,32}$), 3.79 (s, 8H, $H_{30,31}$), 3.28 (t, J = 6.9 Hz, 4H, H_1), 1.86 (quint, J = 6.8 Hz, 4H, H_5), 1.65 (quint, J = 7.9 Hz, 4H, H_2), 1.60-1.53 (m, 22H, $H_{4,14,21,41}$), 1.48 (quint, J = 6.8 Hz, 4H, H_3); **^{13}C NMR** (151 MHz, $CDCl_3$) δ 162.78, 157.35, 157.09, 148.89, 148.88, 139.11, 138.06, 138.04, 137.90, 134.05, 133.96, 133.94, 129.44,

129.42, 129.41, 128.93, 128.91, 128.78, 127.63, 127.61, 127.55, 125.26, 125.14, 125.11, 124.63, 119.63, 119.61, 106.77, 106.51, 71.09, 69.95, 67.90, 67.61, 51.52, 49.04, 29.85, 29.25, 28.95, 26.68, 25.90, 21.65, 21.58, 21.54; ; *Due to the high degree of apparent symmetry within **S19** many ^{13}C signals overlap. **HRMS** (ESI$^+$): Calcd. for $C_{117}H_{124}O_{16}N_{15}$: 1994.9312, found 1994.9345 $[M+H]^+$.

S20

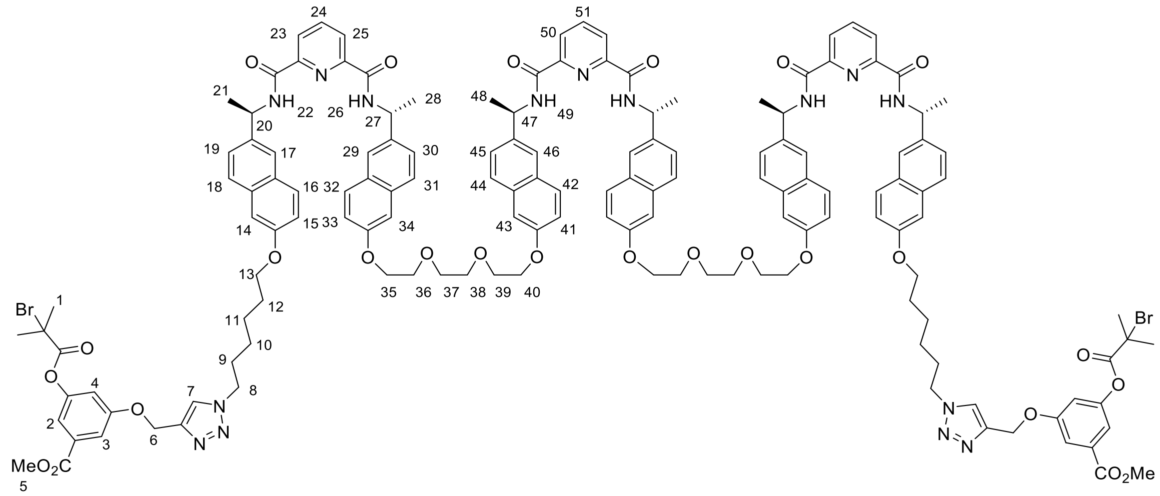

A solution of **S19** (30 mg, 0.015 mmol), **S18** (11 mg, 0.03 mmol) and DIPEA (3 µl, 0.03 mmol) in DCM (4 mL) and MeCN (0.4 mL) was degassed with four freeze-pump-thaw cycles. The mixture was then cannulated to a degassed vial containing $Cu(MeCN)_4PF_6$ (11 mg, 0.03 mmol). The resulting solution was stirred overnight at room temperature, after which the solution was washed with 2% aqueous EDTA (5 mL) and extracted with DCM (3 x 5 mL). The organic phases were combined and dried with Na_2SO_4. The crude product was purified by preparative TLC (4% MeOH in DCM, 2 elutions) yielding **S20** as a colourless solid (24 mg, 60% yield). **^{1}H NMR** (600 MHz, $CDCl_3$) δ 8.29-8.25 (m, 6H, $H_{25,25,50}$), 8.17 (t, *J* = 8.4 Hz, 6H, $H_{22,26,49}$), 7.92-7.89 (m, 3H, $H_{24,51}$), 7.61-7.54 (m, 22H, $H_{7,16,17,18,19,29,31,32,42,44,46}$), 7.47 (t, *J* = 2.3 Hz, 2H, H_2), 7.30-7.34 (m, 8H, $H_{3,19,30,45}$), 7.13-7.08 (m, 6H, $H_{15,33,41}$), 7.05-7.02 (m, 6H, $H_{14,34,43}$), 6.95 (t, *J* = 2.3 Hz, 2H, H_4), 5.42-5.34 (m, 6H, $H_{20,27,47}$), 5.14 (s, 4H, H_6), 4.35 (t, *J* = 7.1 Hz, 4H, H_8), 4.20 (t, *J* = 6.4 Hz, 8H, $H_{35,40}$), 4.02 (t, *J* = 6.3 Hz, 4H, H_{13}), 3.91 (t, *J* = 4.7 Hz, 8H, $H_{36,39}$), 3.84 (s, 6H, H_5), 3.78 (s, 8H, $H_{37,38}$), 2.04 (s, 12H, H_1), 1.86 (quint, *J* = 7.4 Hz, 4H, H_9), 1.83 (quint, *J* = 6.7 Hz, 4H, H_{12}), 1.56-1.46 (m, 22H, $H_{10,21,28,48}$), 1.40 (quint, *J* = 8.3 Hz, 4H, H_{11}); **^{13}C NMR** (151 MHz, $CDCl_3$) δ 170.06, 166.03, 162.83, 158.98, 157.15, 156.97, 151.47, 148.81, 148.78, 143.23, 138.98, 138.00, 137.83, 133.89, 133.83, 133.81, 132.31, 129.36, 129.35, 128.78, 128.77, 128.64, 127.50, 127.42, 125.24, 124.51, 124.50, 122.91, 119.55, 119.43, 115.40, 113.24, 113.20, 106.58, 106.35, 71.01, 69.88, 67.58, 67.48, 62.47, 55.18, 52.62, 50.39, 48.80, 48.72, 30.62, 30.23, 29.01, 26.19, 25.58, 21.41, 21.34, 21.29; *Due to the high degree of apparent symmetry within **S20** many ^{13}C signals overlap. **HRMS** (ESI$^+$): Calcd. for $C_{147}H_{153}O_{26}N_{15}Br_2Na$: 2724.9370, found 2724.9364 $[M+Na]^+$.

S21

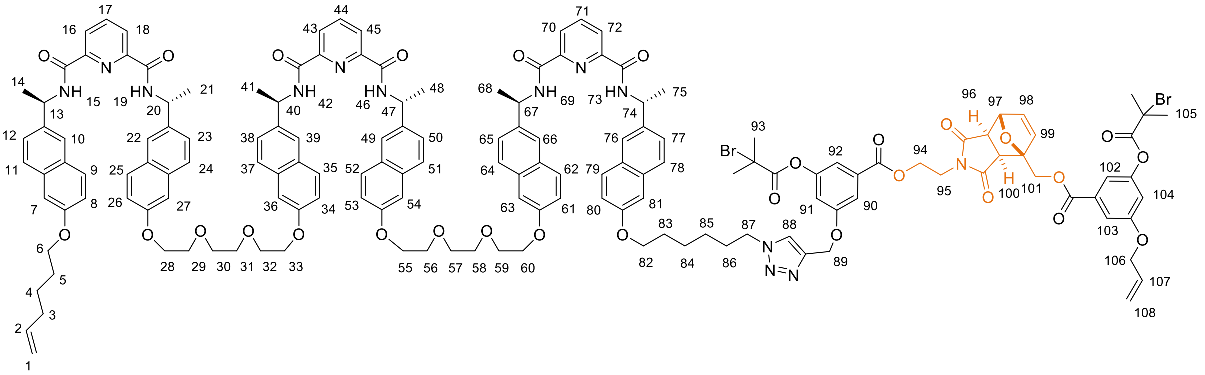

A solution of **S16** (66 mg, 0.034 mmol), **S9** (30 mg, 0.034 mmol) and DIPEA (4 µl, 0.034 mmol) in DCM (5 mL) and MeCN (0.5 mL) was degassed with four freeze-pump-thaw cycles. The mixture was then cannulated to a degassed vial containing $Cu(MeCN)_4PF_6$ (12 mg, 0.034 mmol). The resulting solution was stirred overnight at room temperature, after which the solution was washed with 2% aqueous EDTA (5 mL) and extracted with DCM (3 x 5 mL). The organic phases were combined and dried with Na_2SO_4. The crude product was purified by preparative TLC (3% MeOH in DCM, 2 elutions) yielding **S21** as a colourless solid (66 mg, 70% yield). **^{1}H NMR** (600 MHz, $CDCl_3$) δ 8.36-8.25 (m, 6H, $H_{16,18,43,45,70,72}$), 8.09-7.97 (m, 6H, $H_{15,19,42,46,69,73}$), 7.98-7.87 (m, 3H, $H_{17,44,71}$), 7.66-7.56 (m, 19H, $H_{9,10,11,22,24,25,35,37,39,49,51,52,62,64,66,76,78,79,88}$), 7.51 (s, 1H, H_{90}), 7.46-7.43 (m, 1H, H_{103}), 7.41-7.33 (m, 8H, $H_{12,23,38,50,65,77,92,102}$), 7.14-7.10 (m, 6H, $H_{8,26,34,53,61,80}$), 7.08-7.03 (m, 6H, $H_{7,27,36,54,63,81}$), 6.95 (t, J = 2.3 Hz, 1H, H_{91}), 6.89 (d, J = 2.0 Hz, 1H, H_{104}), 6.53 (dd, J = 5.3, 2.4 Hz, 1H, H_{98}), 6.44 (d, J = 5.8 Hz, 1H, H_{99}), 6.00 (ddt, J = 16.1, 10.4, 5.4 Hz, 1H, H_{107}), 5.84 (ddt, J = 16.9, 10.2, 6.6 Hz, 1H, H_2), 5.43-5.33 (m, 7H, $H_{13,20,40,47,67,74,108trans}$), 5.30 (dd, J = 10.4, 3.8 Hz, 1H, H_{108cis}), 5.25 (s, 1H, H_{97}), 5.20 (s, 2H, H_{89}), 5.14 (dd, J = 13.0, 9.9 Hz, 1H, H_{101}), 5.04 (d, J = 17.1 Hz, 1H, H_{1trans}), 4.98 (d, J = 10.2 Hz, 1H, H_{1cis}), 4.64 (d, J = 12.9 Hz, 1H, H_{101}), 4.55-4.53 (m, 2H, H_{106}), 4.48-4.43 (m, 1H, H_{94}), 4.41-4.37 (m, 1H, H_{94}), 4.35 (t, J = 7.2 Hz, 2H, H_{87}), 4.23-4.21 (m, 8H, $H_{28,33,55,60}$), 4.08-4.03 (m, 4H, $H_{6,82}$), 3.99-3.90 (m, 10H, $H_{29,32,56,59,95}$), 3.78 (s, 8H, $H_{30,31,57,58}$), 3.01 (dd, J = 6.4, 4.0 Hz, 1H, H_{96}), 3.01 (dd, J = 6.5, 4.1 Hz, 1H, H_{100}), 2.15 (q, J = 6.4 Hz, 2H, H_3), 2.04 (s, 6H, $H_{93/105}$), 2.03 (s, 6H, $H_{93/105}$), 1.95 (quint, J = 7.3 Hz, 2H, H_{86}), 1.85 (quint, J = 8.0 Hz, 4H, $H_{5,83}$), 1.65-1.50 (m, 22H, $H_{4,14,21,41,48,68,75,84}$), 1.42 (quint, J = 6.8 Hz, 2H, H_{85}); **^{13}C NMR** (151 MHz, $CDCl_3$) δ 175.55, 174.20, 170.06, 170.03, 165.16, 165.05, 162.82, 162.80, 162.78, 159.49, 159.10, 157.41, 157.29, 157.09, 151.60, 148.94, 148.91, 143.39, 139.08, 138.64, 138.08, 137.95, 137.87, 137.85, 137.04, 134.06, 134.02, 133.96, 133.94, 132.42, 131.98, 131.78, 129.44, 129.42, 129.37, 128.93, 128.91, 128.79, 128.77, 127.61, 127.59, 127.53, 125.25, 125.16, 125.13, 125.08, 124.63, 124.61, 122.89, 119.65, 119.61, 119.53, 115.64, 115.05, 114.95, 113.91, 113.66, 113.35, 113.24, 106.80, 106.53, 71.09, 69.95, 69.46, 67.97, 67.77, 67.62, 62.61, 62.26, 61.78, 55.28, 55.24, 50.44, 50.15, 49.03, 49.00, 48.65, 38.03, 33.59, 30.69, 30.30, 29.84, 29.11, 28.82, 26.37, 25.71, 25.52, 21.63, 21.57, 21.55, 21.52; *Due to the high degree of apparent symmetry within **S21** many ^{13}C signals overlap. **HRMS** (ESI$^+$): Calcd. for $C_{156}H_{160}O_{29}N_{13}Br_2Na$: 1429.9858, found 1429.9849 $[M+H+Na]^{2+}$.

Overhand knot S22

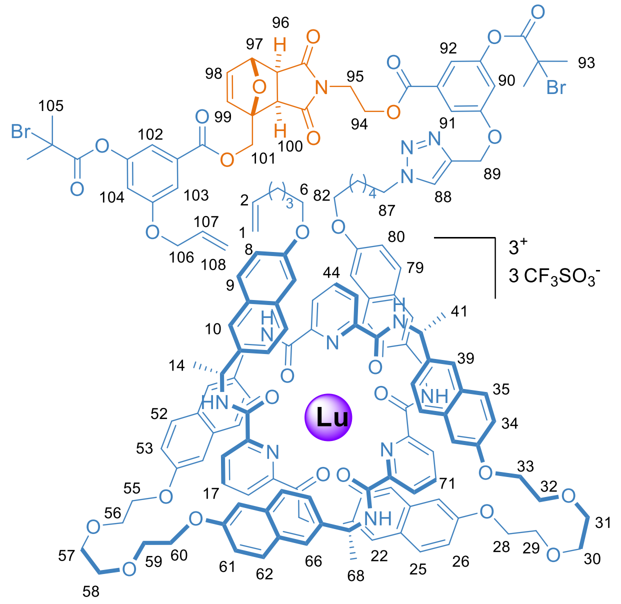

To a vigorously stirred solution of **S21** (60 mg, 0.021 mmol) in MeCN (21 mL) was added a solution of lutetium trifluoromethanesulfonate (13 mg, 0.021 mmol). The reaction was stirred overnight at 70 °C. The mixture was allowed to cool to room temperature. The solution was filtered and concentrated to give overhand knot **S22** (69 mg, 95%) as an off-white powder. **^{1}H NMR** (600 MHz, CD_3CN) δ 8.66 (s, br, 1H, $H_{15/19/42/46/69/73}$), 8.43 (s, br, 1H, $H_{15/19/42/46/69/73}$), 8.32-8.30 (m, br, 2H, $H_{15/19/42/46/69/73}$), 8.27-8.24 (m, br, 2H, $H_{15/19/42/46/69/73}$), 7.90 (s, 1H, H_{88}), 7.62-7.59 (m, 2H, $H_{9,79}$), 7.59-7.52 (m, 5H $H_{25,35,52,62,90}$), 7.50-7.46 (m, 3H, $H_{11,78,103}$), 7.44 (s, 1H, $H_{43/45}$), 7.39 (s, 1H, $H_{43/45}$), 7.30-7.16 (m, 17H, $H_{8,24,26,27,34,36,37,44,51,53,54,61,63,64,80,92,102}$), 7.14-7.10 (m, 2H, $H_{38,49}$), 7.05-7.03 (m, 2H, $H_{7,81}$), 7.01-6.98 (m, 2H, $H_{12/66,22/76}$), 6.97-6.90 (m, 6H, $H_{12/66,22/76,16/18,70/72,91,104}$), 6.86-6.73 (m, 6H, $H_{16/18,70/72,10,23,65,77}$), 6.70-6.65 (m, 2H, $H_{39,50}$), 6.57-6.53 (m, 1H, H_{98}), 6.52-6.50 (m, 1H, H_{99}), 6.08 (app td, J = 7.9, 1.9 Hz, 2H, $H_{17,71}$), 6.02 (ddt, J = 16.5, 10.4, 5.4 Hz, 1H, H_{107}), 5.92 (ddt, J = 16.1, 10.4, 5.0 Hz, 1H, H_2), 5.42-5.35 (m, 1H, $H_{108trans}$), 5.31-5.25 (m, 1H, H_{108cis}), 5.20-5.08 (m, 4H, $H_{89,97,101}$), 5.01 (d, J = 10.4 Hz, 1H, H_1), 5.01 (d, J = 12.6 Hz, 1H, H_1), 4.77-4.75 (m, 1H, $H_{13/20/40/47/67/74}$), 4.70-4.60 (m, 4H, $H_{13/20/40/47/67/74,101}$), 4.59-4.52 (m, 4H, $H_{13/20/40/47/67/74,106}$), 4.47-4.37 (m, 4H, $H_{87,94}$), 4.35-4.22 (m, 8H, $H_{28,33,55,60}$), 4.19 (t, J = 5.8 Hz, 2H, $H_{6/82}$), 4.12 (t, J = 4.8 Hz, 2H, $H_{6/82}$), 4.09-4.03 (m, 4H, $H_{29/32/56/59}$), 4.00-3.92 (m, 4H, $H_{29/32/56/59}$), 3.90-3.74 (m, 8H, $H_{30,31,57,58}$), 3.69-3.65 (m, 2H, H_{95}), 3.03-2.97 (m, 2H, $H_{96,100}$), 2.26-2.19 (m, 2H, H_3), 2.07-1.98 (m, 12H, $H_{93,105}$), 1.95-1.90 (m, 2H, H_{86}, overlaps with solvent peak), 1.83-1.79 (m, 2H, $H_{5/83}$), 1.68 (quint, J = 8.0 Hz, 2H, $H_{5/83}$), 1.62-1.40 (m, 24H, $H_{4,14,21,41,48,68,75,84,85}$); **^{13}C NMR** (151 MHz, CD_3CN) 177.09,175.81, 170.99, 167.66, 167.37, 167,12, 137.09, 165.66, 165.58, 156.51, 160.62, 160,54, 160.09, 158.31, 158.23, 158.08, 157.89, 157.88, 152.68, 152.67, 152.54, 150.48, 150.40, 145.35, 145.33, 144.75, 144.68, 144.64, 144.62, 143.51, 139.96, 139.82, 139.41, 139.34, 138.93, 138.83, 138.74, 138.47, 137.80, 135.35, 134.70, 134.65, 134.37, 134.35, 134.21, 134.05, 133.87, 133.83, 133.21, 133.19, 132.97, 130.87, 130.10, 130.07, 129.98, 129.81, 129.79, 129.46, 129.34, 129.27, 129.25, 129.07, 128.18, 128.11, 128.09, 128.03, 127.95, 126.82, 126.38, 125.26, 125.21, 124.38, 124.30, 124.24, 124.11, 124.00, 123.42, 123.40, 123.36, 123.33, 123.26, 123.14, 121.01 120.49, 120.45, 120.36, 115.88, 115.41, 115.35, 115.33, 115.30, 114.58, 114.33, 114.27, 114.02, 113.95, 113.83, 112.04, 111.75, 108.04, 107.76, 90.21, 82.04, 71.66, 71.61, 71.57, 70.14, 69.99, 69.33, 69.25, 69.17, 68.81, 68.74, 63.38, 62.96, 62.43, 59.74, 57.03, 57.00, 59.95, 56.62, 55.97, 54.17, 54.08, 53.70, 53.66, 52.99, 52.86, 51.03, 51.01, 49.47, 44.00, 38.36, 35.23, 34.20, 31.99, 30.77, 30.74, 30.71, 30.60, 30.14, 29.53, 29.47, 26.65, 26.22, 26.17, 23.54, 23.38, 23.30, 22.18, 22.10, 20.86; *Due to the high degree of apparent symmetry within overhand knot **2** many ^{13}C signals overlap; **HRMS** (ESI$^+$): Calcd. for $C_{156}H_{159}O_{29}N_{13}Br_2Lu$: 1004.9727, found 1004.9720 $[M-3(CF_3SO_3)]^{3+}$.

Gated trefoil knot Lu-3_1 S23

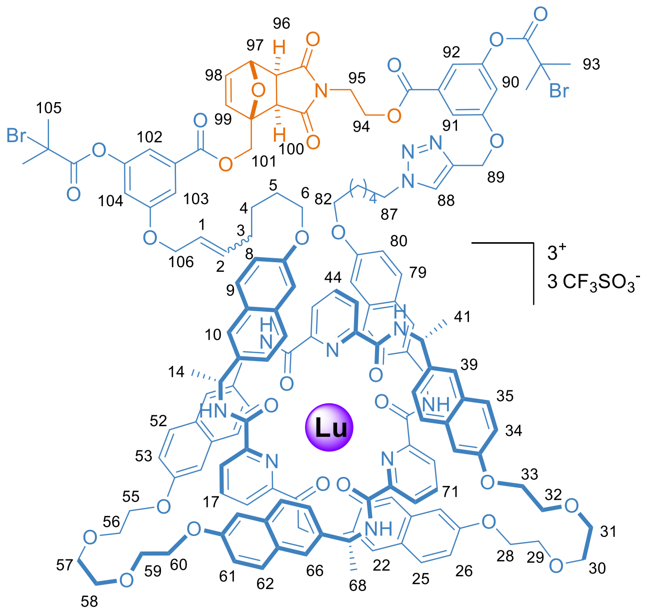

To a stirred solution of overhand knot **S22** (25 mg, 7.2 µmol) in CH_3NO_2 (freshly degassed by purging with a N_2 stream for 30 min, 3.6 mL) was added a solution of Hoveyda-Grubbs 2nd generation catalyst (4.5 mg, 7.2 µmol) in DCM (freshly degassed by purging with a N_2 stream for 30 min, 3.6 mL) under N_2. The reaction was stirred for 20 h at 50 °C, after which ethyl vinyl ether (1 mL) was added. The quenched reaction was stirred for 1 h at RT, after which the mixture was concentrated under reduced pressure. Further purification by size exclusion chromatography (DCM as eluent) yielded gated trefoil knot Lu-3_1 **S23** (5.1 mg, 25 %) as a brown solid. Note that the isolated yield is only representative of the size exclusion fractions of high purity. The actual amount of knot formed is considerably higher. The ^{1}H NMR spectrum of the compound is broad at RT due to fluxionality in the molecule. **^{1}H NMR** (600 MHz, CD_3CN/MeOD 10:1) δ 7.62-7.59 (m, 11H, $H_{9,11,25,35,52,62,78,79,88,90,103}$), 7.42-7.05 (m, 21H $H_{8,24,26,27,34,36,37,38,43,44,45,49,51,53,54,61,63,64,80,92,102}$), 7.04-6.89 (m, 10H, $H_{7,16/18,70/72,12,22,66,76,81,91,104}$), 6.88-6.70 (m, 8H, $H_{16/18,70/72,10,23,39,50,65,77}$), 6.57-6.40 (m, 2H, $H_{98,99}$), 6.03-5.92 (m, 4H, $H_{1,2,17,71}$), 5.30-5.07 (m, 4H, $H_{89,97,101}$), 4.91-4.87 (m, 1H, H_{101}), 4.75-4.53 (m, 8H, $H_{13,20,40,47,67,74,106}$), 4.48-4.21 (m, 12H, $H_{28,33,55,60,87,94}$), 4.19-4.03 (m, 8H, $H_{6,29/32/56/59,82}$), 3.98-3.76 (m, 14H, $H_{29/32/65/59,30,31,57,58,95}$), 3.05-2.94 (m, 2H, $H_{96,100}$), 2.37-2.26 (m, 2H, H_3), 2.04-1.99 (m, 12H, $H_{93,105}$), 1.73-1.40 (m, 30H, $H_{4,5,14,21,41,48,68,75,83,84,85,86}$); **^{13}C NMR** (151 MHz, CD_3CN/MeOD 10:1) δ 177.56, 176.27, 171.31, 171.28, 171.26, 167.86, 167.61, 167.43, 165.96, 160.35, 158.50, 158.35, 158.26, 158.20, 158.15, 152.92, 152.88, 152.84, 152.81, 145.27, 144.93, 143.89, 140.16, 139.85, 139.79, 139.74, 138.72, 138.65, 138.54, 138.04, 136.90, 134.95, 134.80, 134.63, 134.40, 133.42, 133.16, 130.54, 130.32, 130.29, 130.19, 130.12, 129.65, 129.54, 129.49, 129.44, 129.39, 128.37, 128.32, 128.30, 128.18, 125.51, 125.26, 124.97, 124.50, 124.33, 123.78, 123.49, 123.32, 123.14, 121.02, 120.64, 120.61, 116.18, 115.52, 114.74, 114.58, 114.46, 108.29, 108.24, 107.94, 102.75, 102.65, 102.63, 90.52, 90.51, 90.48, 82.31, 71.94, 71.87, 70.30, 69.55, 69.43, 69.17, 69.13, 69.07, 63.64, 63.50, 63.14, 57.24, 57.17, 55.45, 54.18, 53.77, 53.23, 51.29, 51.23, 38.70, 38.62, 37.03, 32.99, 32.89, 31.68, 30.95, 30.91, 30.60, 30.01, 29.87, 29.82, 27.12, 27.03, 26.57, 26.50, 23.64, 23.50, 22.86, 22.85, 22.28, 21.24, 21.18, 21.18, 18.63, 18.59, 18.28, 17.98, 15.84; *Due to the pseudo-symmetry and fluxionality, many ^{13}C signals overlap and are not well-resolved; **HRMS** (ESI$^+$): Calcd. for $C_{154}H_{155}O_{29}N_{13}Br_2Lu$: 995.6282, found 995.6272 $[M-3(CF_3SO_3)]^{3+}$.

5.2 Demetallation of trefoil knot Lu-3_1 S23

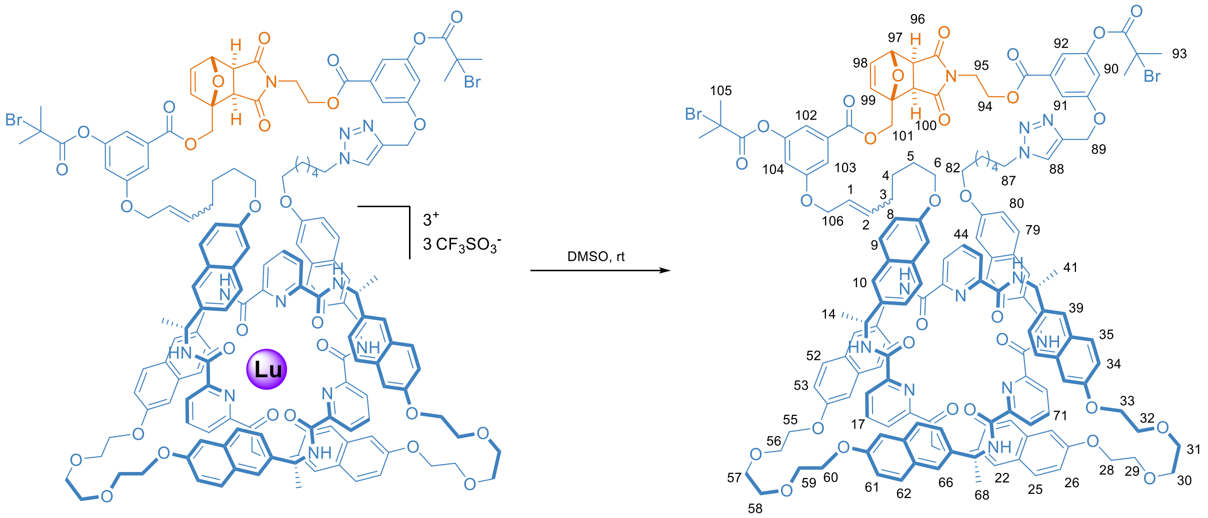

Knot **S23** (3.4 mg, 1.0 μmol) was dissolved in DMSO-d_6 (0.5 mL) by stirring under air. The organic knot was checked directly by ^{1}H NMR spectroscopy at different times, and no changes after the first ^{1}H NMR spectrum recorded immediately upon mixing were observed. This indicates that the demetallation process is very fast (Spectrum 18). The organic product could be identified with MALDI-MS (Supplementary Figure 4). The gated organic knot **S24** exhibited broadened ^{1}H NMR spectral signals due to reptation of the knotted strand. **^{1}H NMR** (600 MHz, DMSO-d_6) δ 10.08 (br, s, 2H, $H_{15/19/42/46/69/73}$), 9.98 (br, s, 2H, $H_{15/19/42/46/69/73}$), 9.46 (br, s, 2H, $H_{15/19/42/46/69/73}$), 8.29-8.00 (m, 10H, $H_{16,17,18,43,44,45,70,71,72,88}$), 7.83-7.68 (m, 10H, $H_{9,11,25,35,52,62,78,79,90,103}$), 7.64-7.03 (m, 26H, $H_{7,8,12,22,24,26,27,34,36,37,38,49,51,53,54,61,63,64,66,76,80,81,91,92,102,104}$), 6.95-6.70 (m, 6H, $H_{10,23,39,50,65,77}$), 6.60-6.54 (m, 2H, $H_{98,99}$), 5.87-5.75 (m, 2H, $H_{1,2}$), 5.44-4.98 (m, 11H, $H_{13,20,40,47,67,74,89,97,101}$) 4.73-4.53 (m, 6H, $H_{87,94,106}$), 4.46-4.21 (m, 8H, $H_{28,33,55,60}$), 4.20-4.11 (m, 4H, $H_{6,82}$), 4.06-3.90 (m, 8H, $H_{29,32,56,59}$), 3.88-3.75 (m, 10H, $H_{30,31,57,58,95}$), 3.12-3.08 (m, 2H, $H_{96,100}$, overlaps with solvent peak), 2.28-2.17 (m, 2H, H_3), 2.06-1.95 (m, 12H, $H_{93,105}$), 1.90-1.82 (m, 2H, H_{86}), 1.69-1.33 (m, 28H, $H_{4,5,14,21,41,48,68,75,83,84,85}$); **^{13}C NMR** (151 MHz, DMSO-d_6) δ 143.46, 139.44, 138.99, 138.89, 138.50, 137.50, 133.31, 133.15, 133.14, 132.88, 131.44, 129.25, 129.11, 128.93, 128.23, 127.92, 126.93, 126.71, 125.56, 124.79, 124.08, 122.15, 121.77, 119.63, 118.90, 117.50, 107.18, 106.78, 106.55, 80.58, 72.39, 70.46, 70.40, 70.01, 68.97, 68.84, 68.78, 68.05, 67.93, 67.17, 61.88, 60.23, 56.75, 54.96, 49.40, 48.17, 29.98, 29.95, 29.66, 25.65, 25.04, 23.03, 21.71; *Due to the high degree of symmetry, many ^{13}C signals were not resolved.

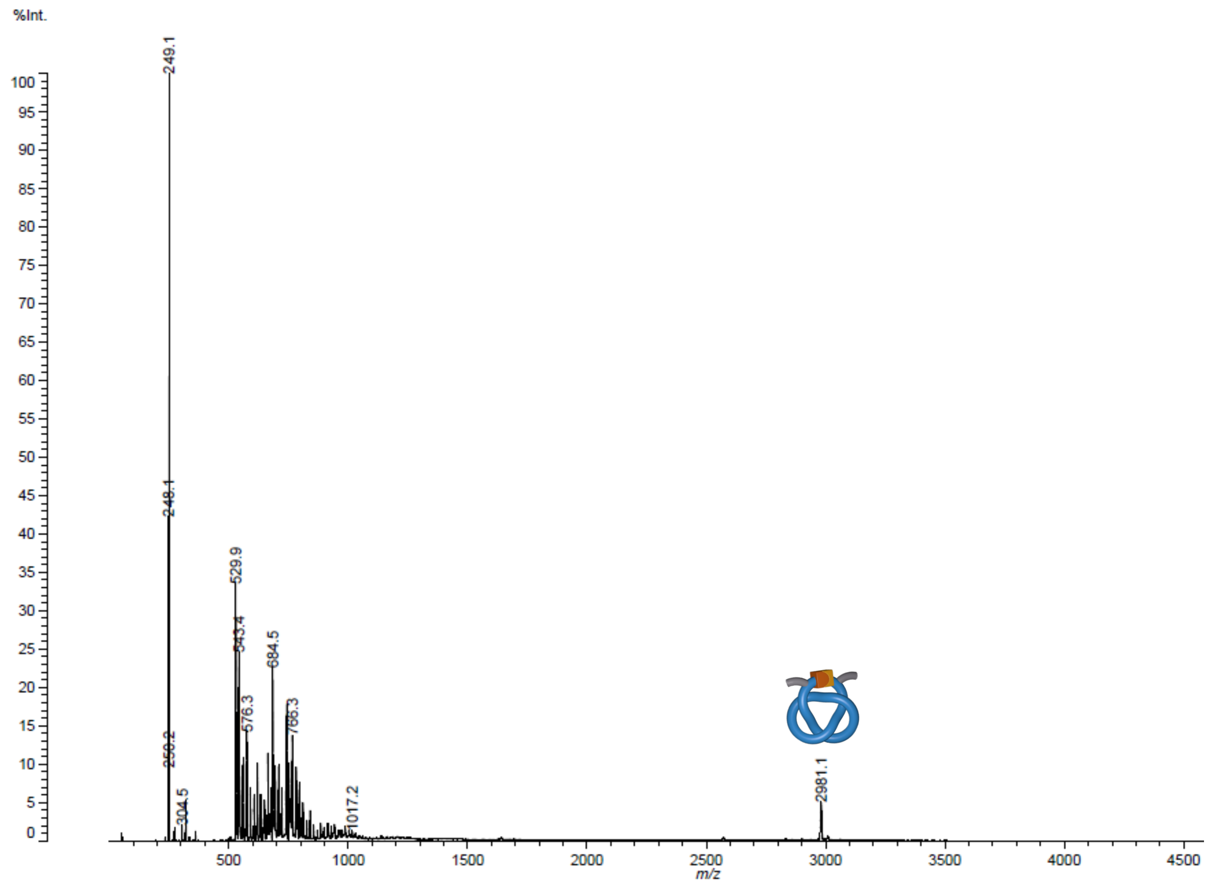

Spectrum 19. MALDI-TOF MS (positive mode, α-Cyano-4-hydroxycinammic acid matrix) of gated organic trefoil knot **S24**. Peak observed as the $[M+2DMSO\text{-}d_6+H]^+$ adduct. Calculated peak (m/z): 2981.1.

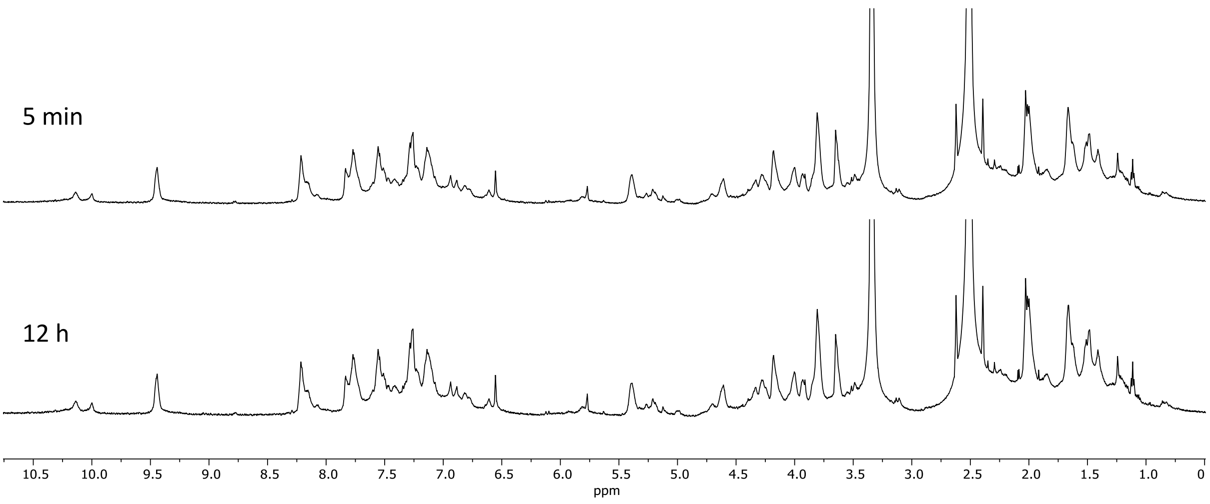

Spectrum 20. ^{1}H NMR spectra (600 MHz, DMSO-d_6) of the demetallation process after 5 min (top) and 12 h (bottom), indicating essentially full demetallation in the first minutes of reaction time.

6 Polymers

6.1 Representative procedure for the synthesis of mechanophore-linked PMA

Methyl acrylate was filtered through basic alumina to remove the inhibitor (≤ 100 ppm monomethyl ether hydroquinone) prior to use. A stock solution of Me_6TREN (16 µL, 0.060 mmol) and $CuBr_2$ (5.6 mg, 0.025 mmol) in dry DMSO (1 mL) was prepared. To a 5 mL vial was added gated organic knot **S24** (4 mg, 1.2 µmol, 1.0 eq.), 9 µL of catalytic solution (Me_6TREN: 3.0 µmol, 0.48 eq.; $CuBr_2$: 1.2 µmol, 0.2 eq.), methyl acrylate (0.11 mL, 1.2 mmol, 1000.0 eq.) and dry DMSO (0.3 mL). This solution was degassed by bubbling with N_2 for 10 min. A Cu(0) wire (~2 cm, ~20 mg, 0.315 mmol, ~100.0 eq., cleaned in HCl_{conc} for 10 min) wrapped around a stirrer bar was added and the solution degassed for a further 2 min before being allowed to stir for 1 h. The solution was then precipitated out into stirring methanol, recovered and dried under high vacuum for several days yielding a colourless polymer (65 mg, M_n = 62 kDa, M_p = 77 kDa). Molecular weight and polydispersity indices were recorded using an analytical GPC that had been calibrated with polystyrene standards.

6.2 List of polymers synthesised

The following polymers were synthesised using the above method.

Table 2. M_n and $Đ$ values for polymers **1**, **2**, **3** and reference polymers **S30**, **S31** and **S32**.

Polymer	M_n / kDa	M_p / kDa	$Đ$
**1**	62.1	77.5	1.27
**2**	71.3	82.2	1.23
**3**	67.4	80.3	1.13
**S30**	45.5	47.2	1.15
**S31**	116.0	135.7	1.13
**S32**	199.0	235.8	1.30

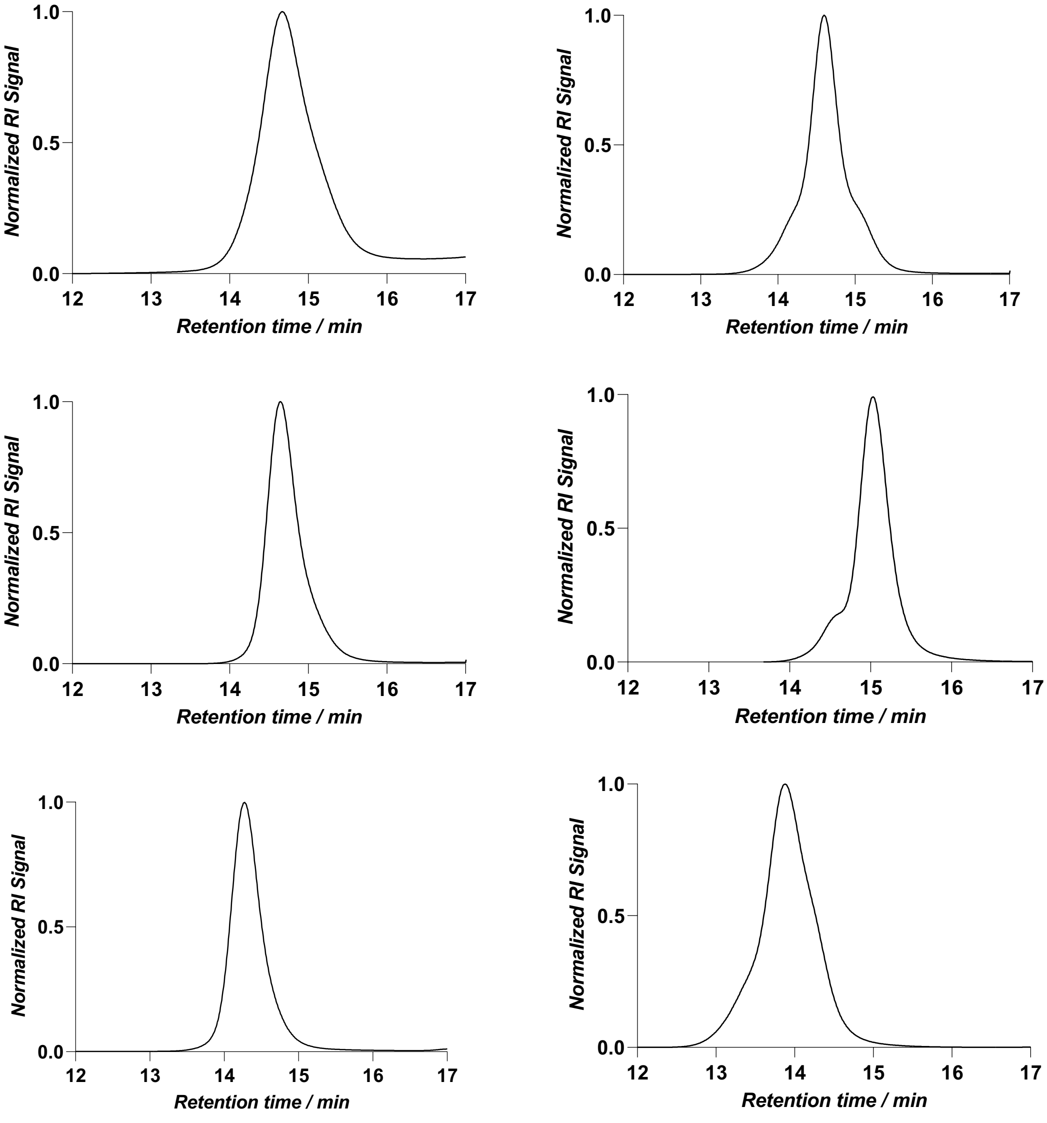

Spectrum 21. GPC traces of polymers **1**, **2**, **3** and reference polymers **S30**, **S31** and **S32**.

7 MS spectra

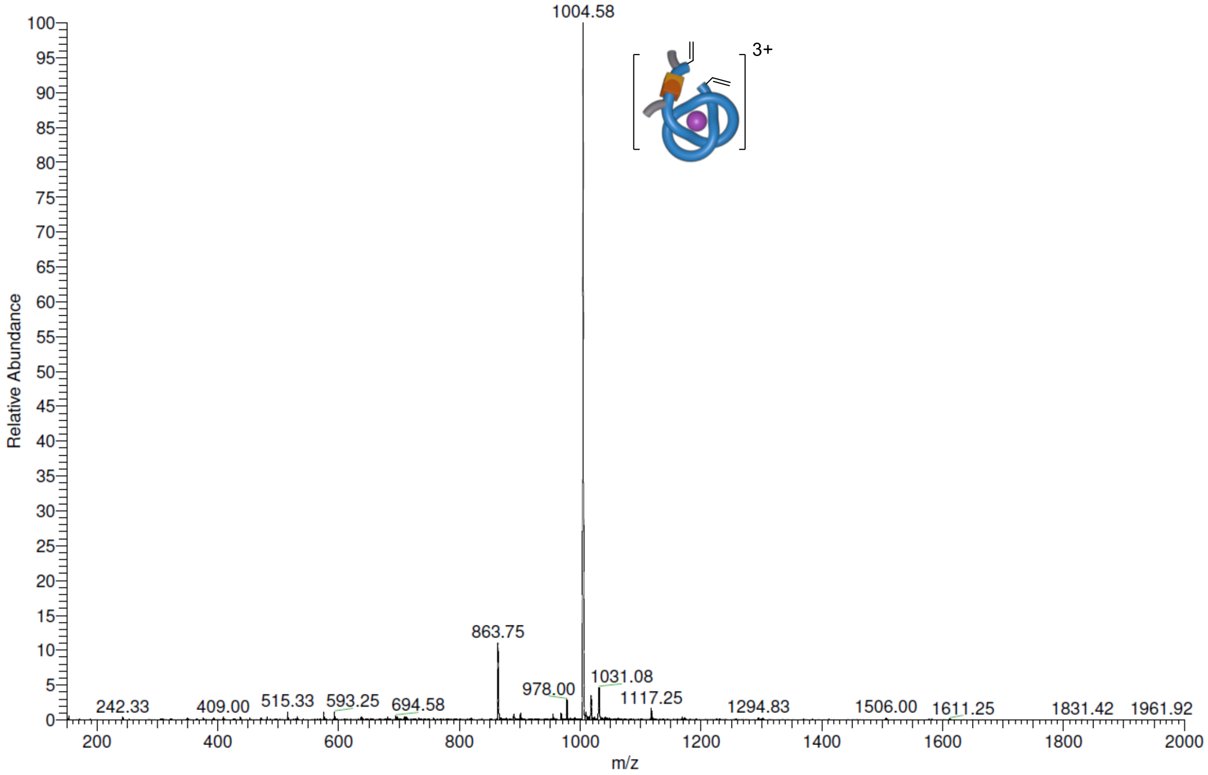

Spectrum 22. Low-resolution ESI-MS(+) of overhand knot **S22**.

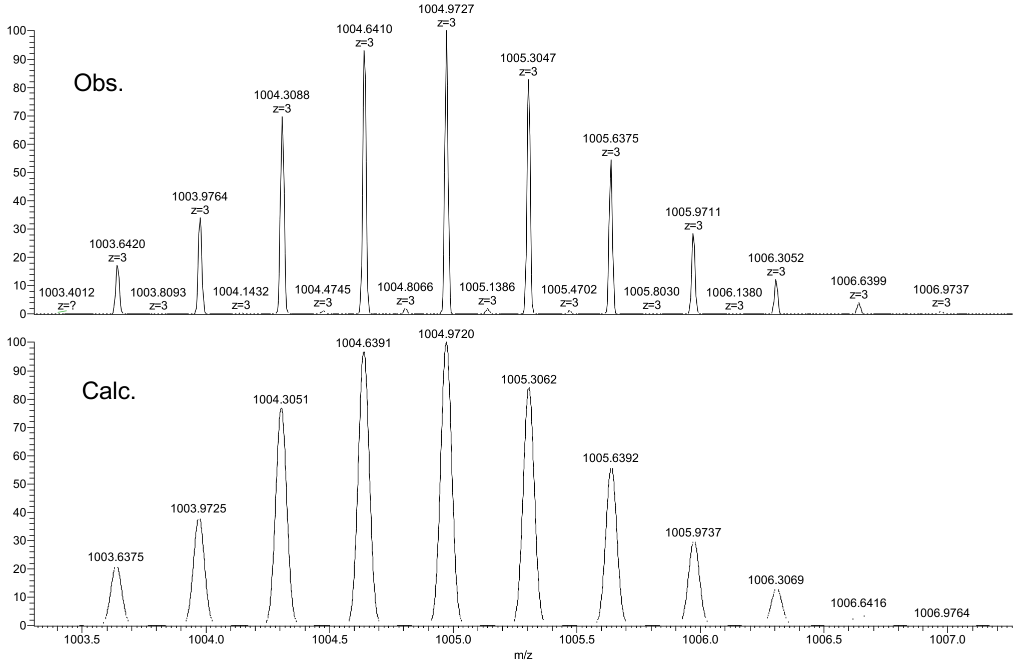

Spectrum 23. High-resolution ESI-MS isotopic distribution of overhand knot **S22**. Top: Measured isotopic distribution for $C_{156}H_{159}Br_2N_{13}O_{29}Lu$ ($[M]^{3+}$, +ESI). Bottom: Simulated isotopic distribution for $C_{156}H_{159}Br_2N_{13}O_{29}Lu$.

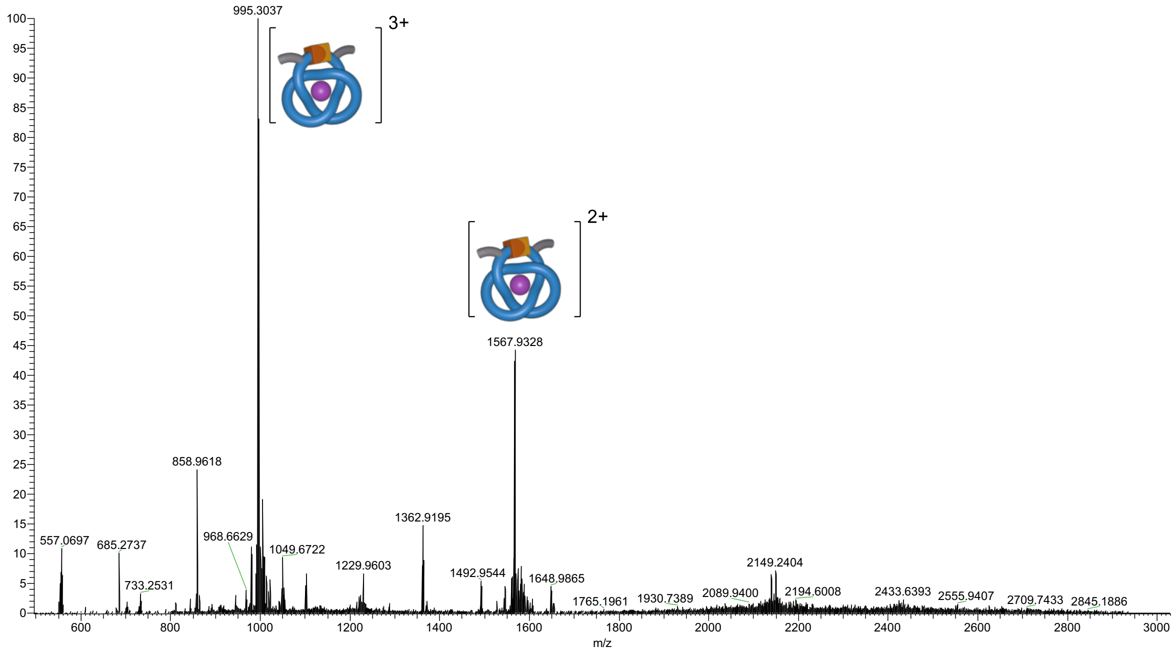

Spectrum 24. High-resolution ESI-MS(+) of gated trefoil knot Lu-3_1 **S23**. Calibrated by internal standard.

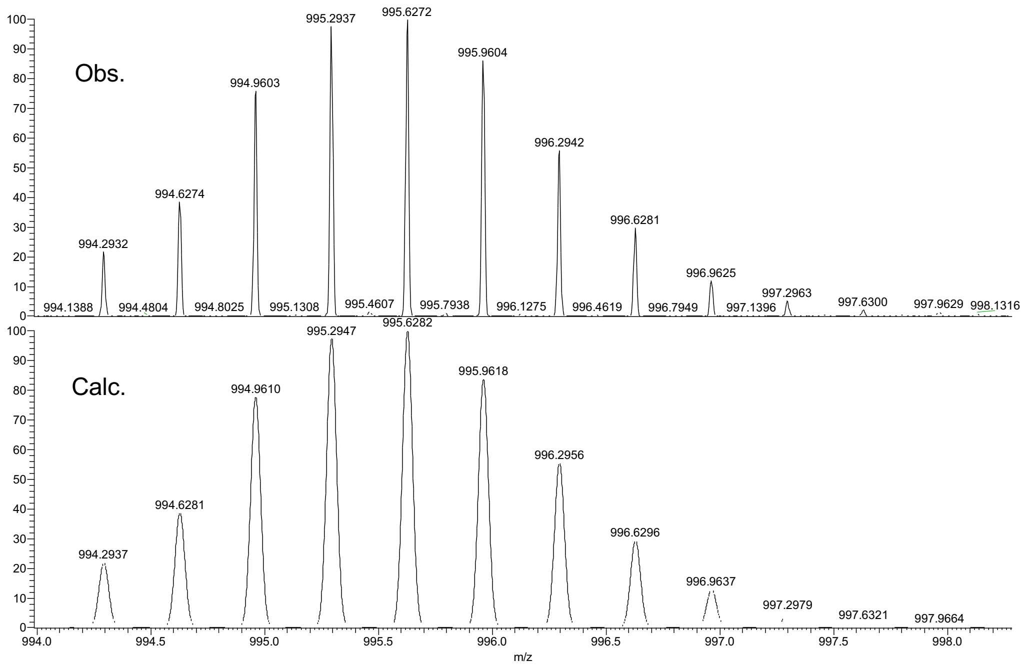

Spectrum 25. High-resolution ESI-MS isotopic distribution of gated trefoil knot Lu-3_1 **S23**. Top: Measured isotopic distribution for $C_{154}H_{155}Br_2N_{13}O_{29}Lu$ ($[M]^{3+}$, +ESI). Bottom: Simulated isotopic distribution for $C_{154}H_{155}Br_2N_{13}O_{29}LuCF_3SO_3$.

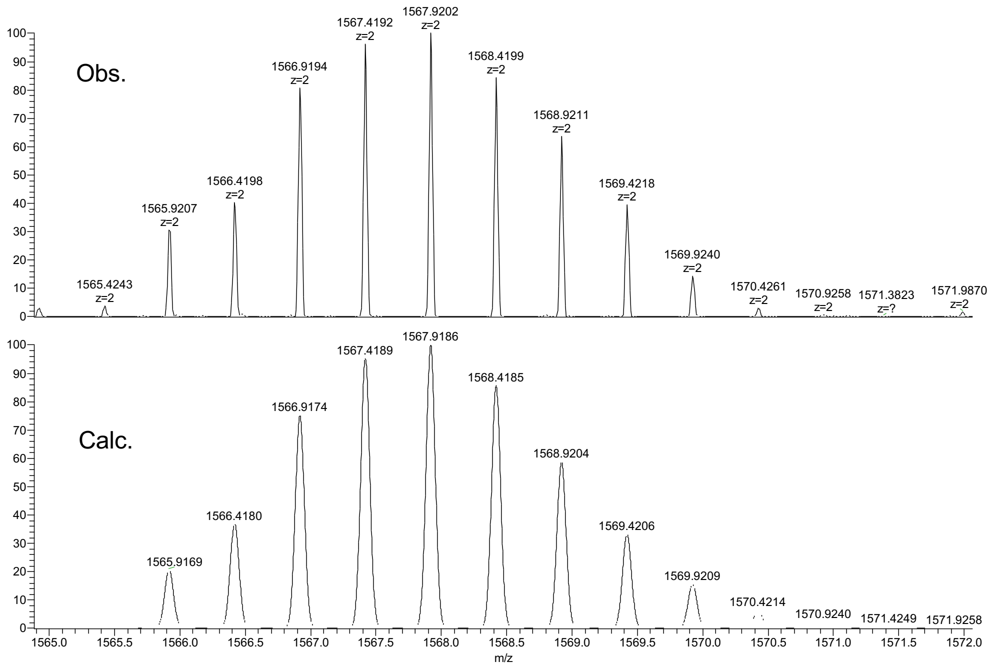

Spectrum 26. High-resolution ESI-MS isotopic distribution of gated trefoil knot Lu-3_1 **S23**. Top: Measured isotopic distribution for $C_{154}H_{155}Br_2N_{13}O_{29}LuCF_3SO_3$ ($[M+CF_3SO_3]^{2+}$, +ESI). Bottom: Simulated isotopic distribution for $C_{154}H_{155}Br_2N_{13}O_{29}LuCF_3SO_3$.

8 NMR Spectra

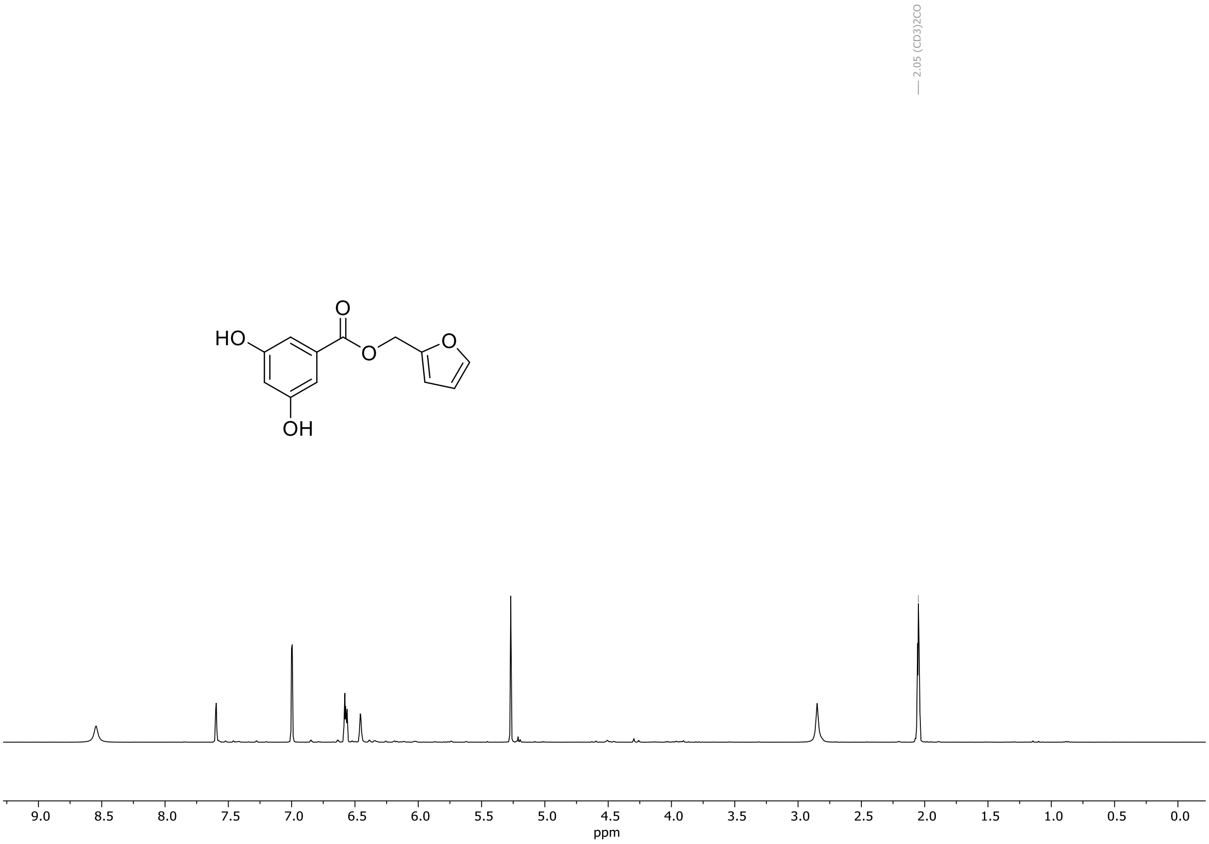

Spectrum 27. ^{1}H NMR spectrum (400 MHz, $(CD_3)_2CO$, 298 K) of compound **S1**.

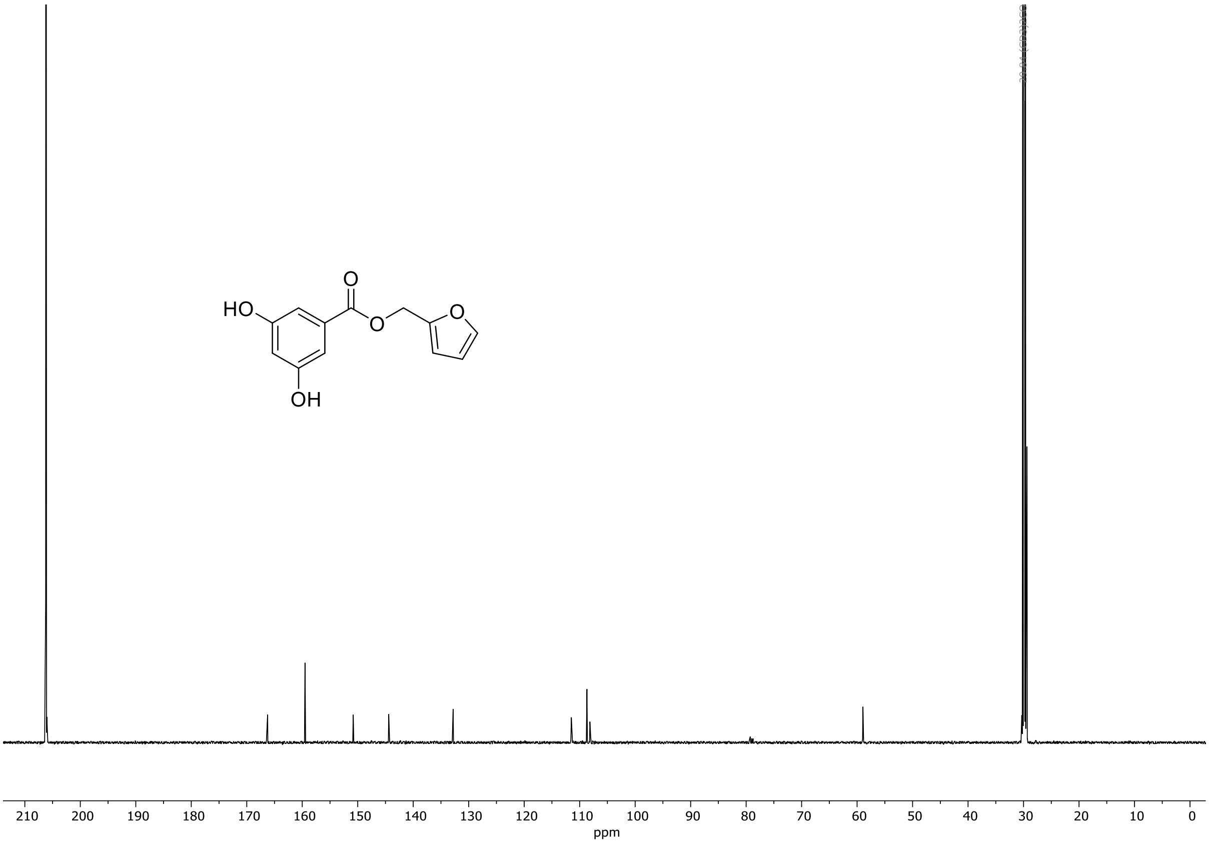

Spectrum 28. ^{13}C NMR spectrum (101 MHz, $(CD_3)_2CO$, 298 K) of compound **S1**.

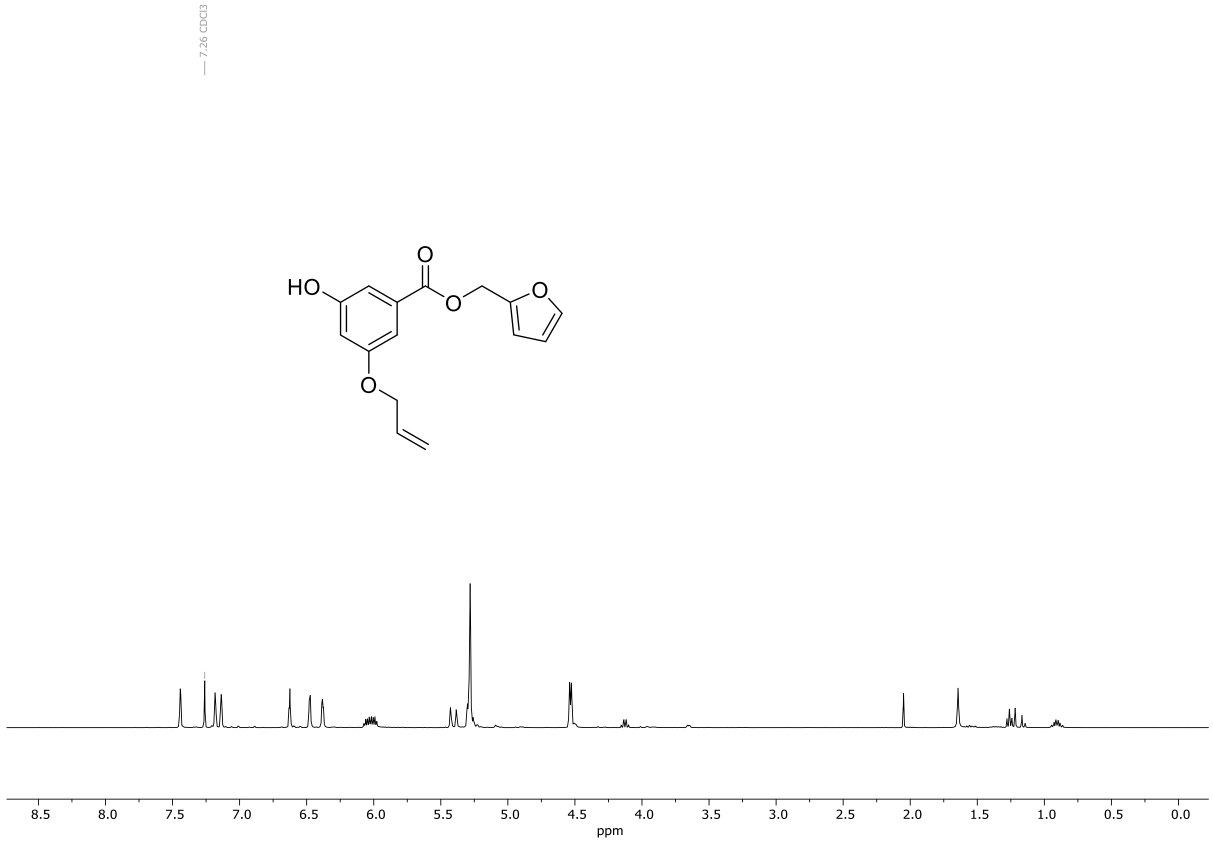

Spectrum 29. ^{1}H NMR spectrum (400 MHz, $CDCl_3$, 298 K) of compound **S2**.

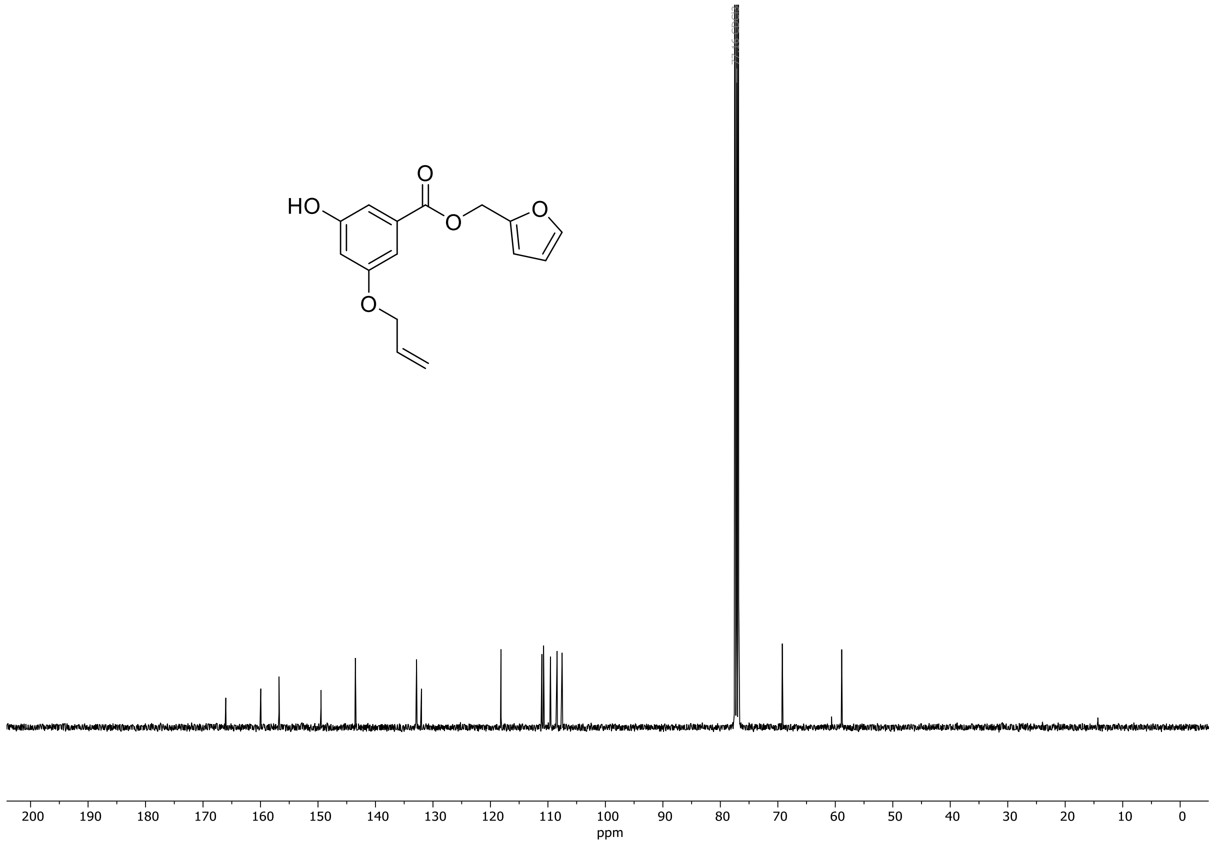

Spectrum 30. ^{13}C NMR spectrum (101 MHz, $CDCl_3$, 298 K) of compound **S2**.

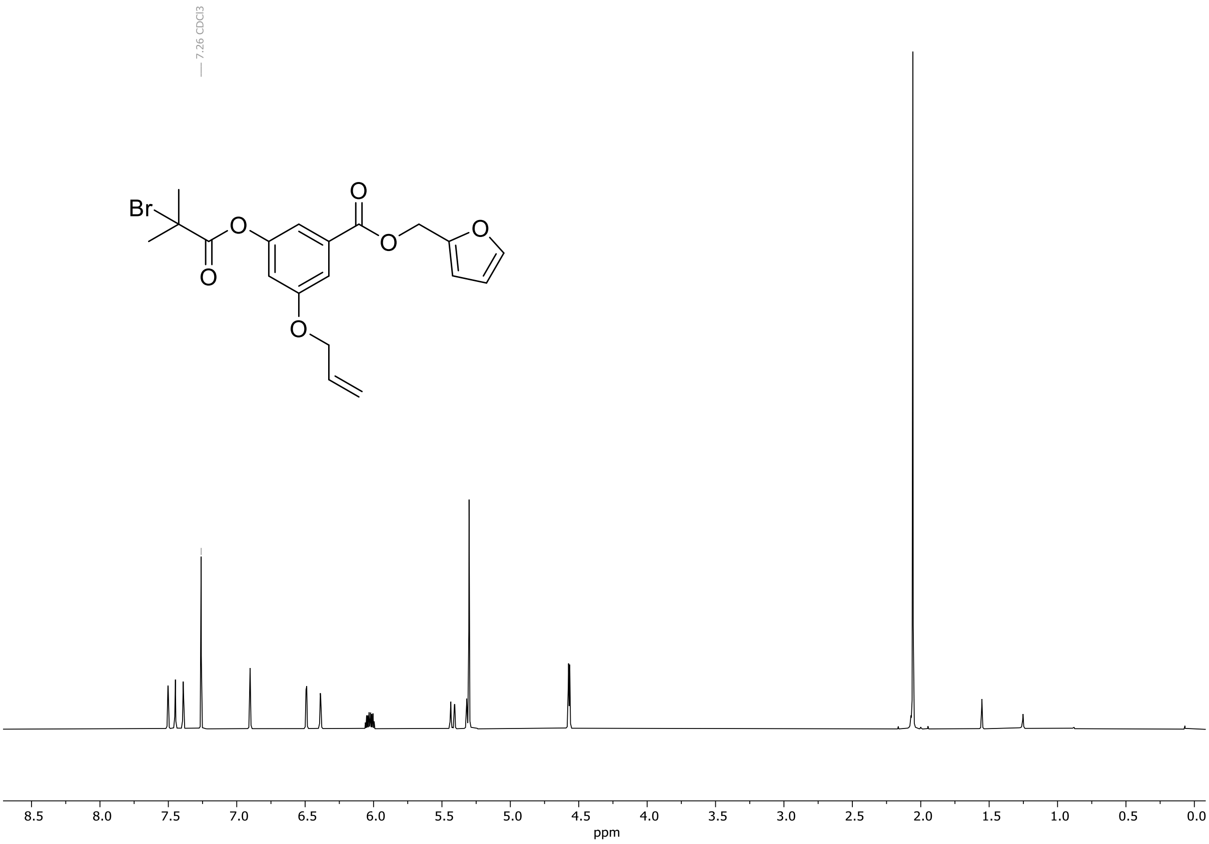

Spectrum 31. ^{1}H NMR spectrum (600 MHz, $CDCl_3$, 298 K) of compound **S3**.

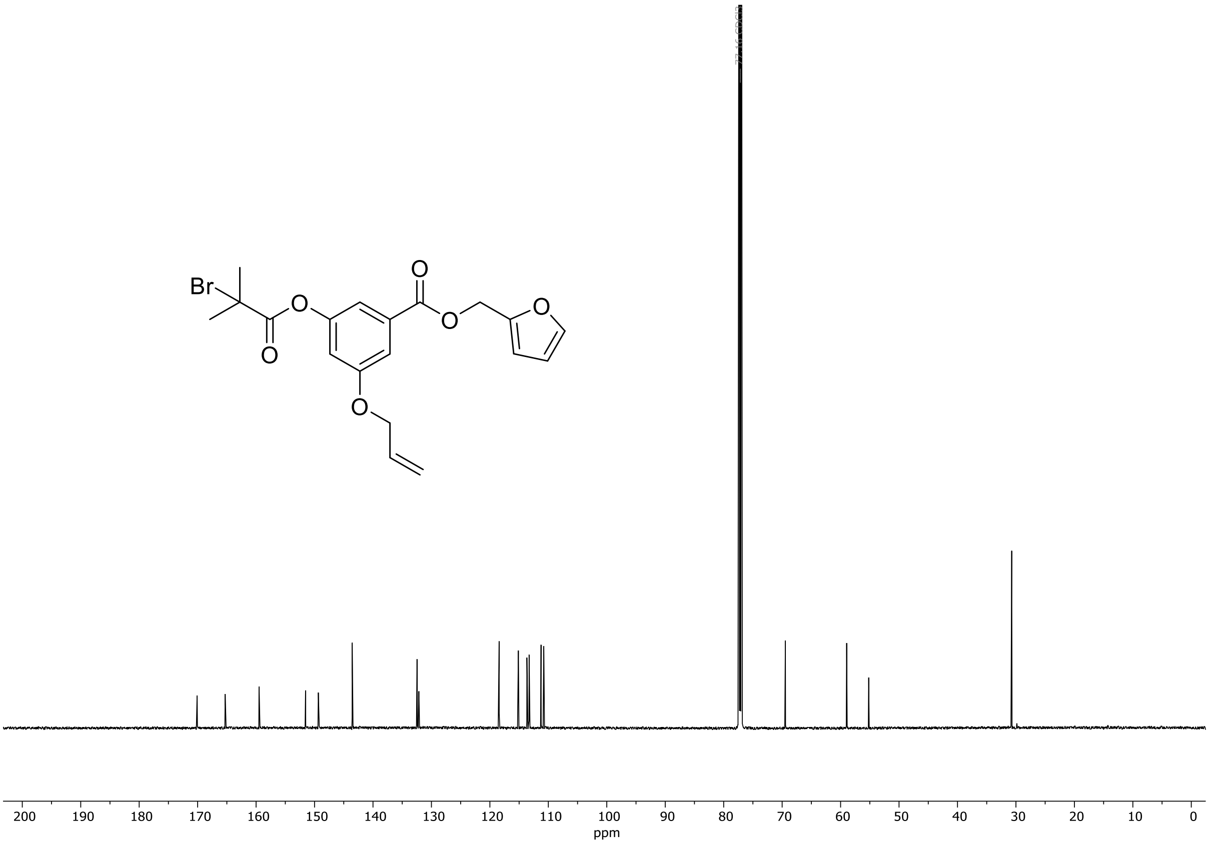

Spectrum 32. ^{13}C NMR spectrum (151 MHz, $CDCl_3$, 298 K) of compound **S3**.

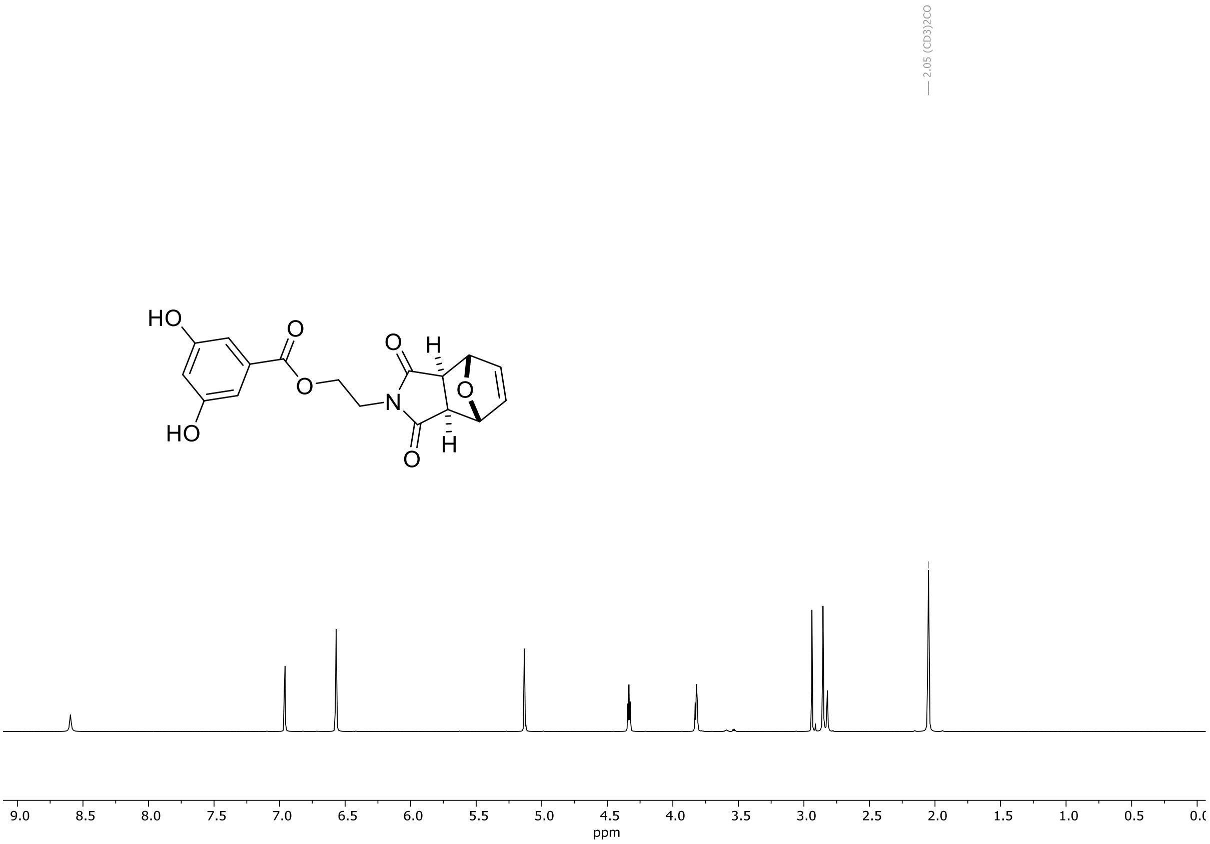

Spectrum 33. ^{1}H NMR spectrum (400 MHz, $(CD_3)_2CO$, 298 K) of compound **S5**.

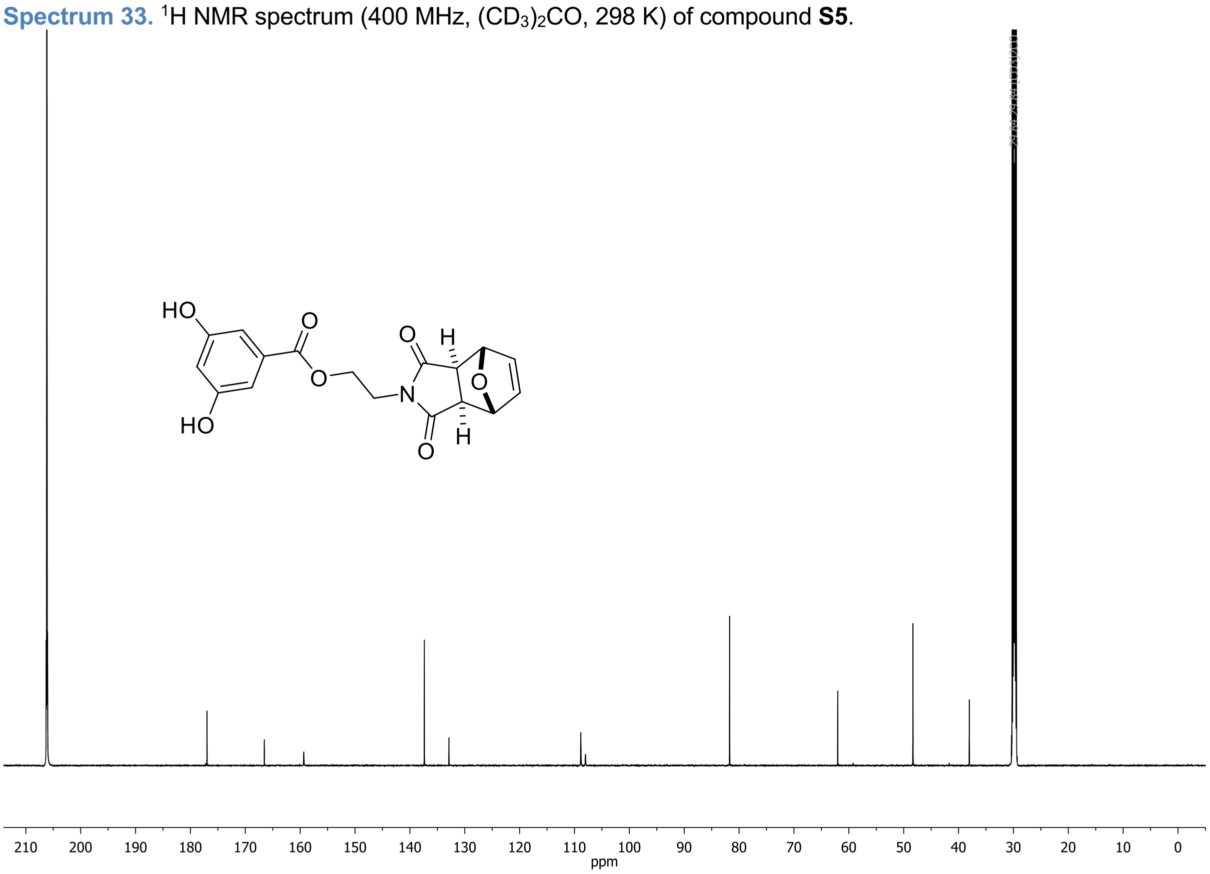

Spectrum 34. ^{13}C NMR spectrum (101 MHz, $(CD_3)_2CO$, 298 K) of compound **S5**.

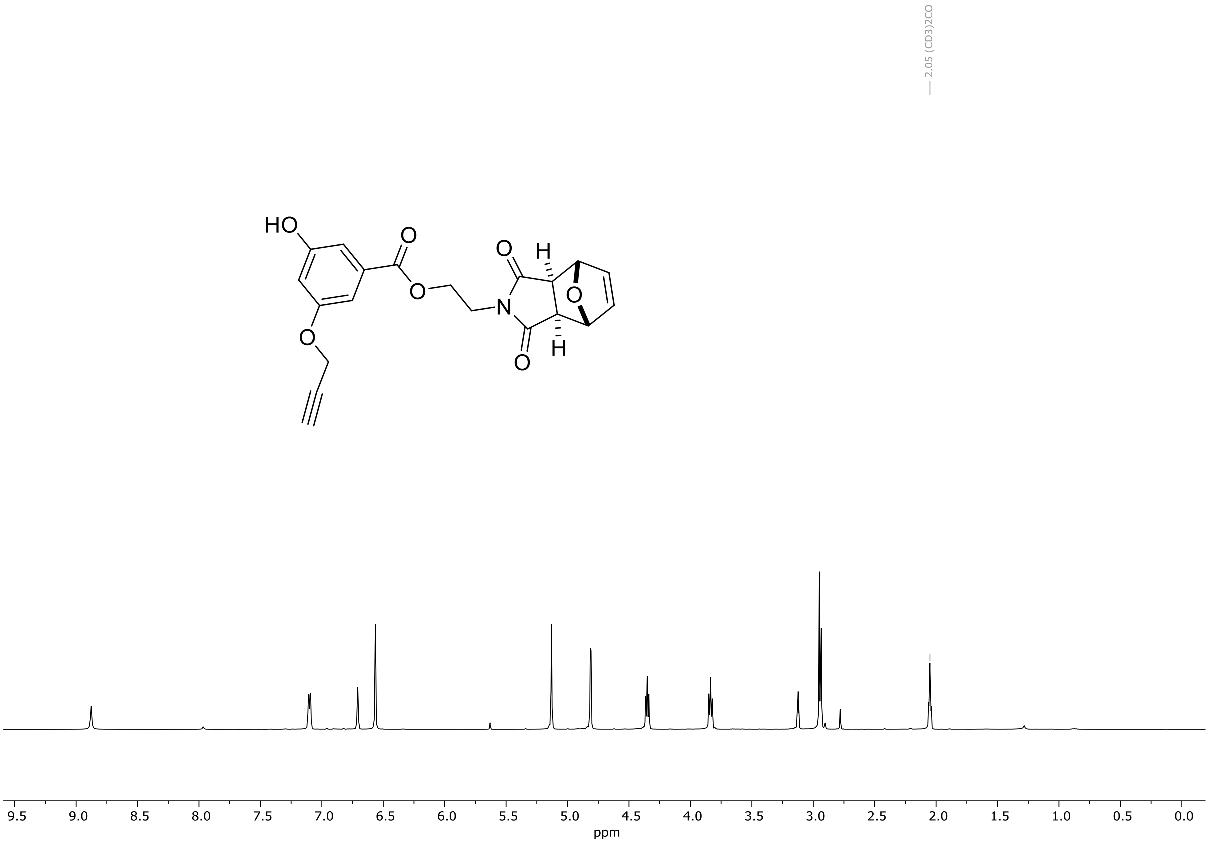

Spectrum 35. ^{1}H NMR spectrum (500 MHz, $(CD_3)_2CO$, 298 K) of compound **S6**.

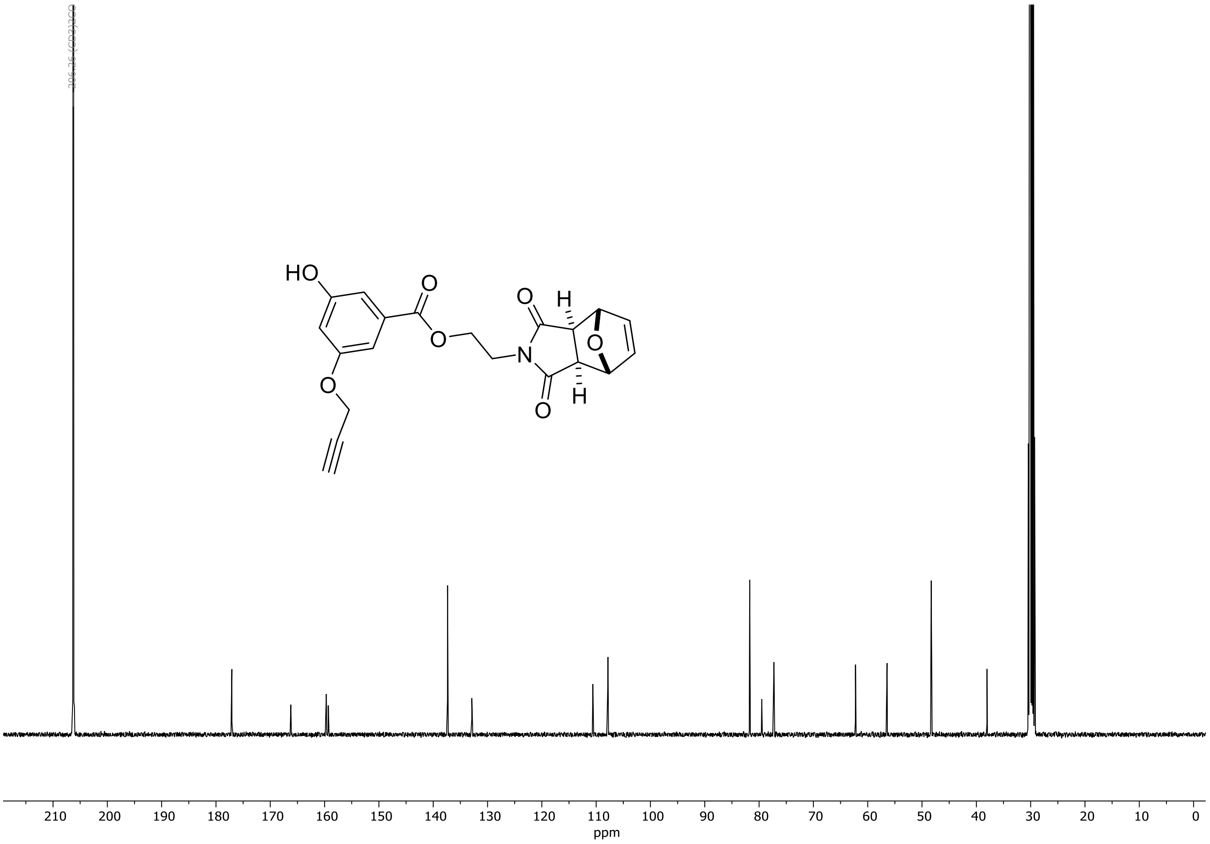

Spectrum 36. ^{13}C NMR spectrum (101 MHz, $(CD_3)_2CO$, 298 K) of compound **S6**.

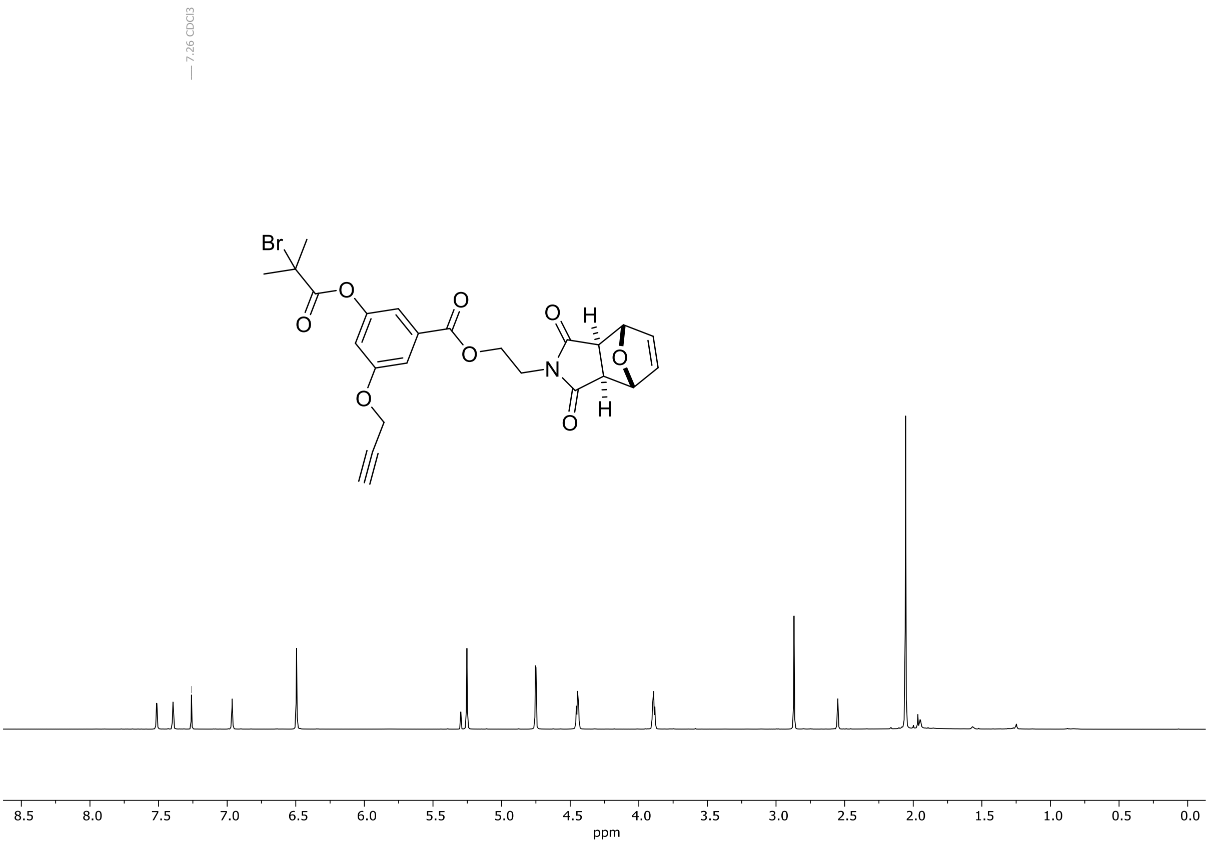

Spectrum 37. ^{1}H NMR spectrum (400 MHz, $CDCl_3$, 298 K) of compound **S7**.

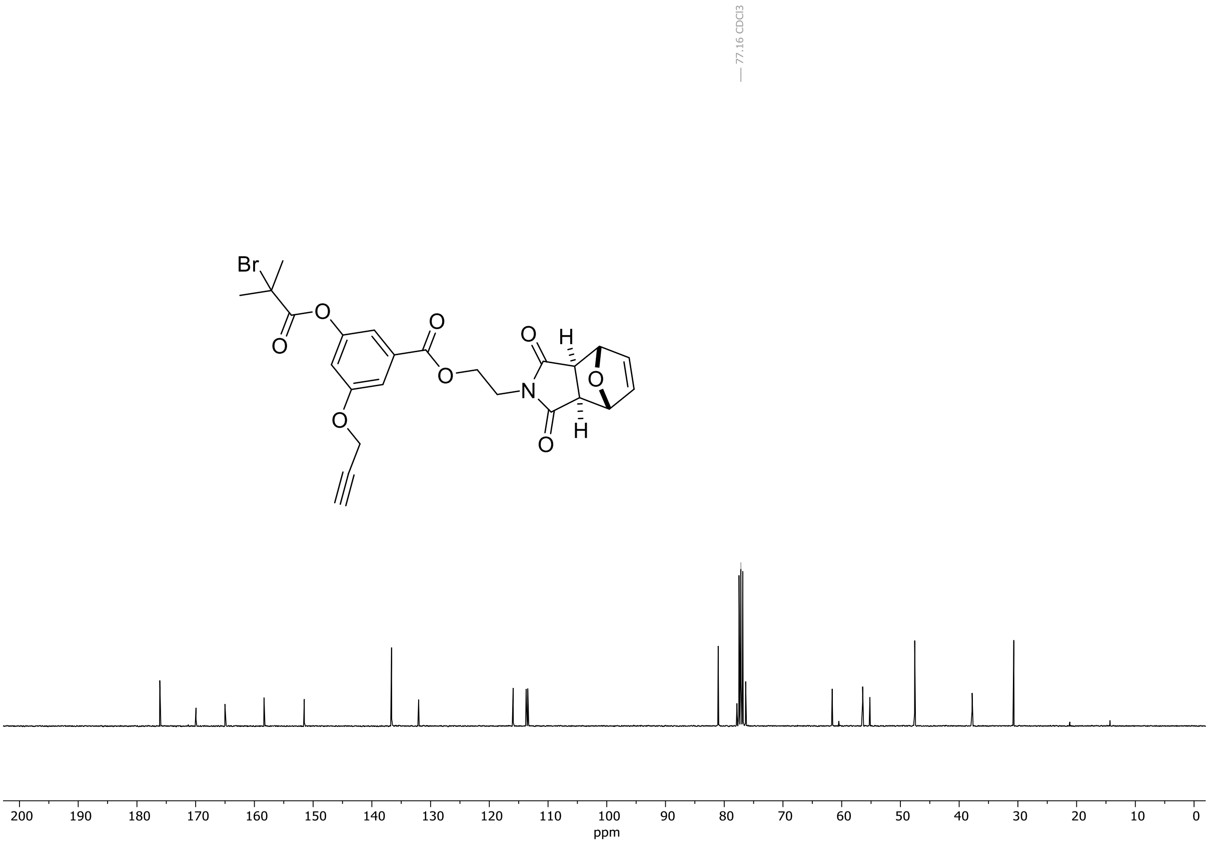

Spectrum 38. ^{13}C NMR spectrum (101 MHz, $CDCl_3$, 298 K) of compound **S7**.

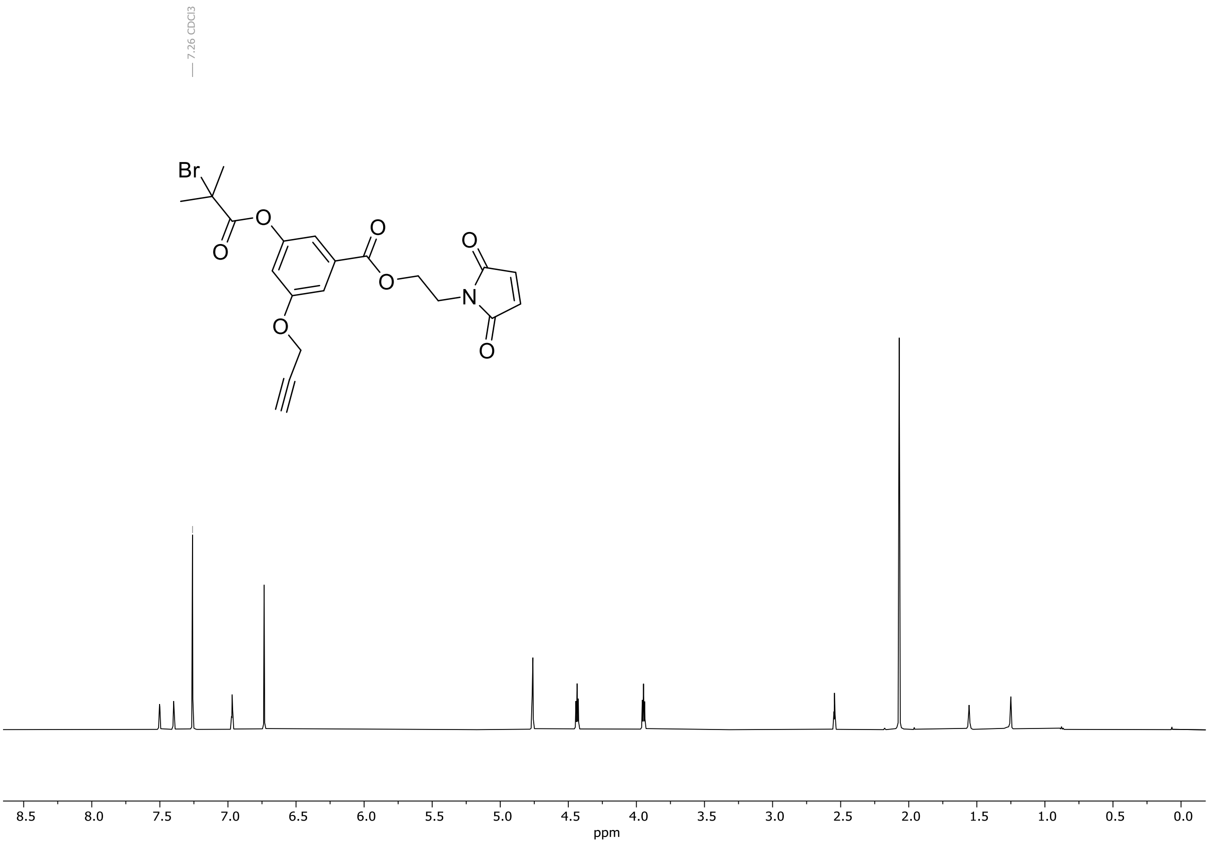

Spectrum 39. ^{1}H NMR spectrum (600 MHz, $CDCl_3$, 298 K) of compound **S8**.

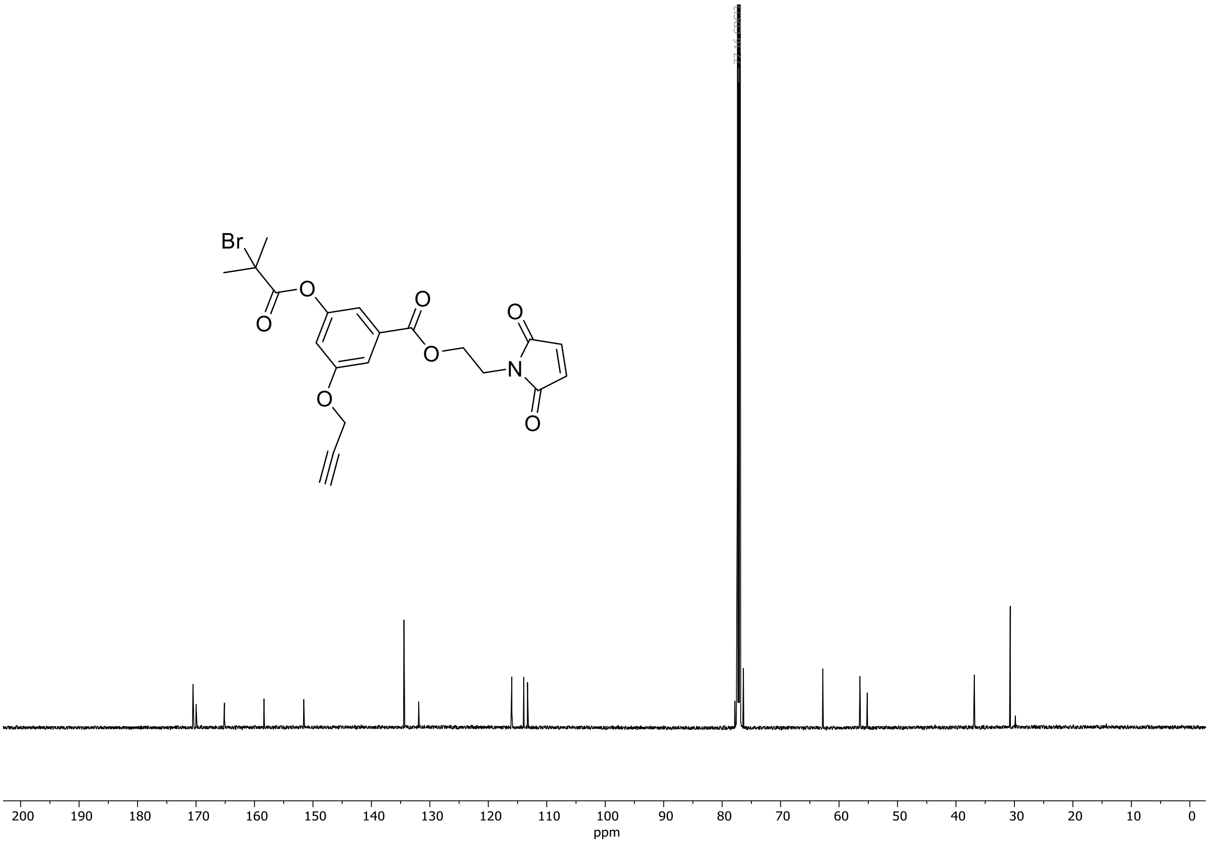

Spectrum 40. ^{13}C NMR spectrum (151 MHz, $CDCl_3$, 298 K) of compound **S8**.

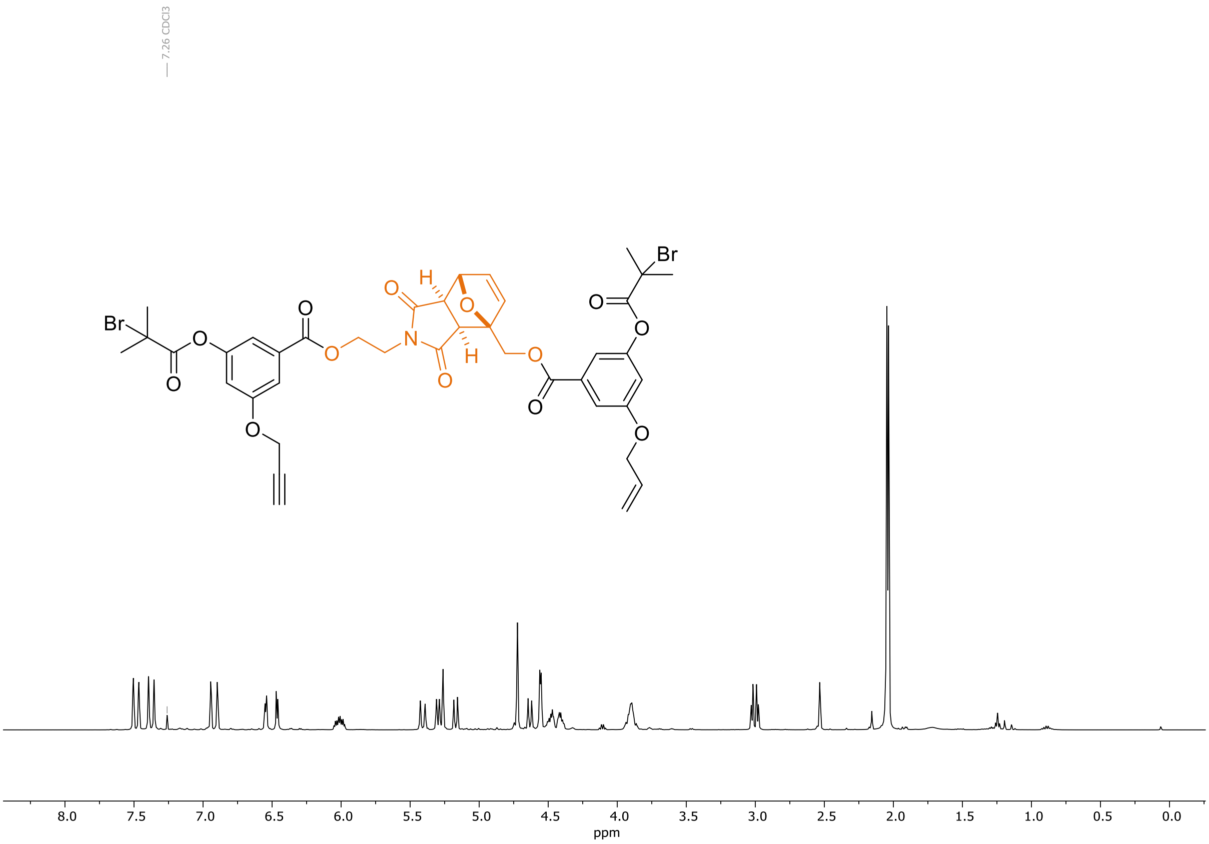

Spectrum 41. ^{1}H NMR spectrum (400 MHz, $CDCl_3$, 298 K) of compound **S9**.

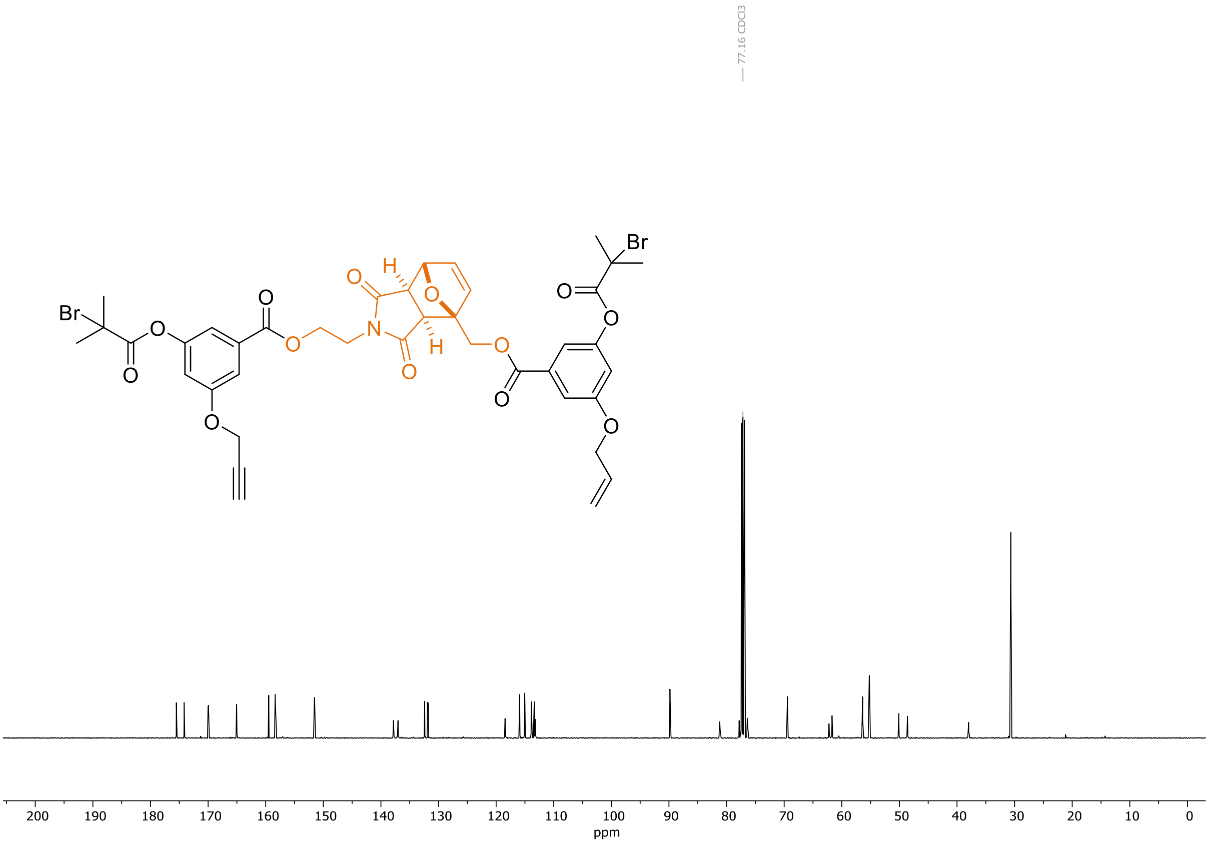

Spectrum 42. ^{13}C NMR spectrum (101 MHz, $CDCl_3$, 298 K) of compound **S9**.

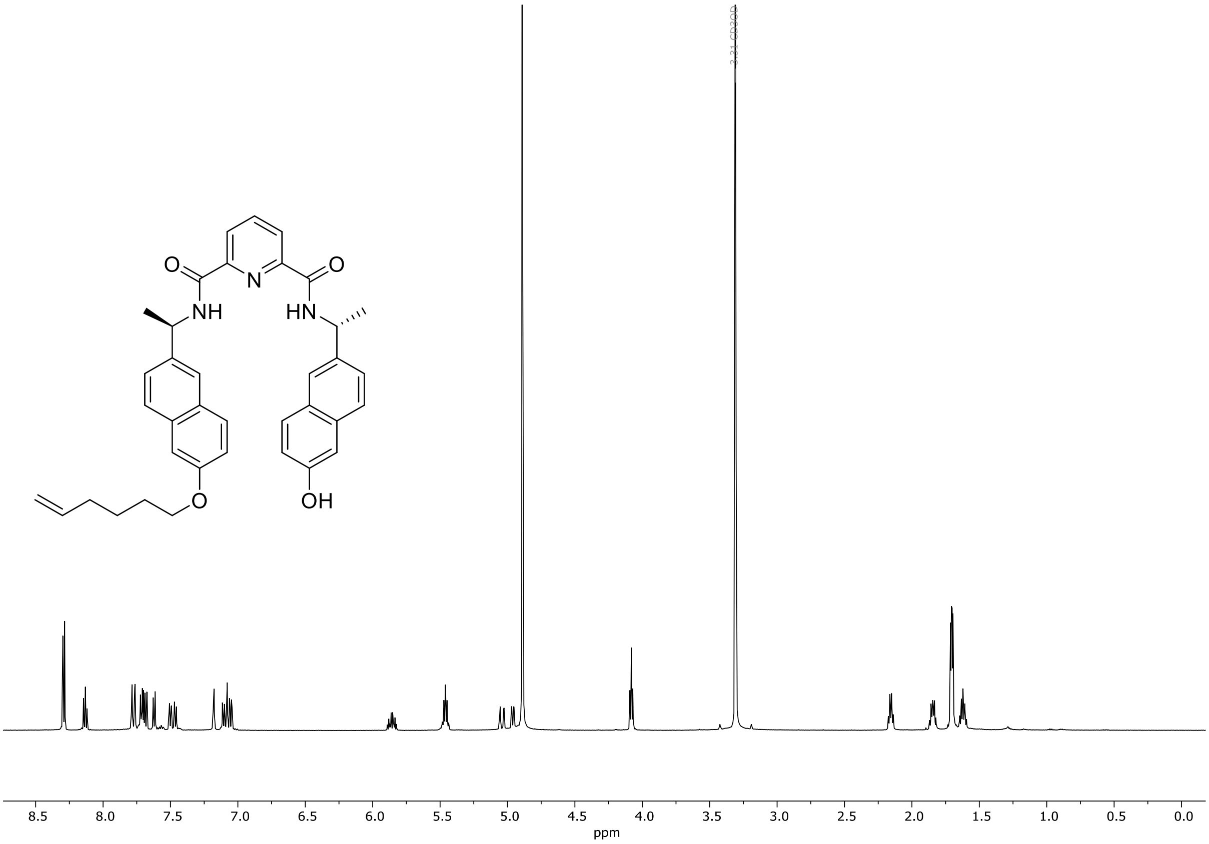

Spectrum 43. ^{1}H NMR spectrum (600 MHz, MeOD-d_4, 298 K) of compound **S11**.

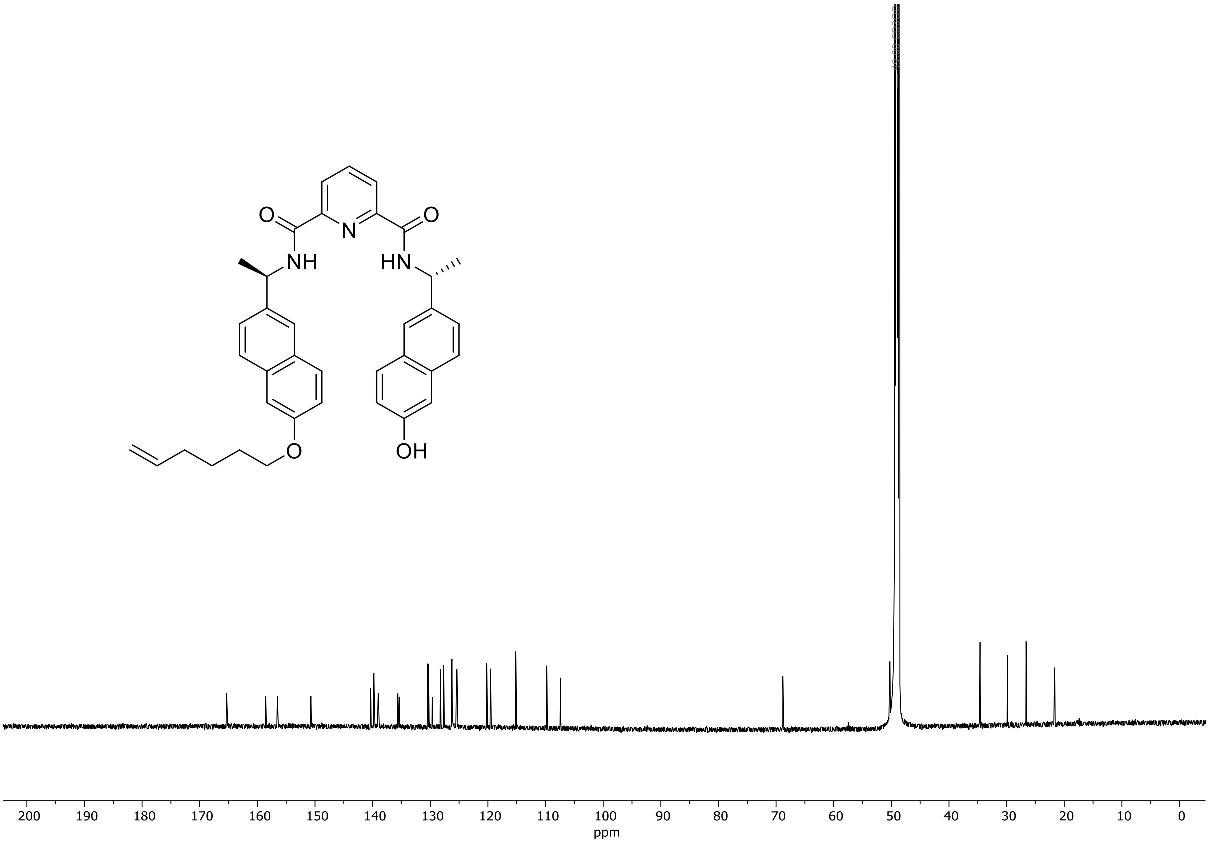

Spectrum 44. ^{13}C NMR spectrum (151 MHz, MeOD-d_4, 298 K) of compound **S11**.

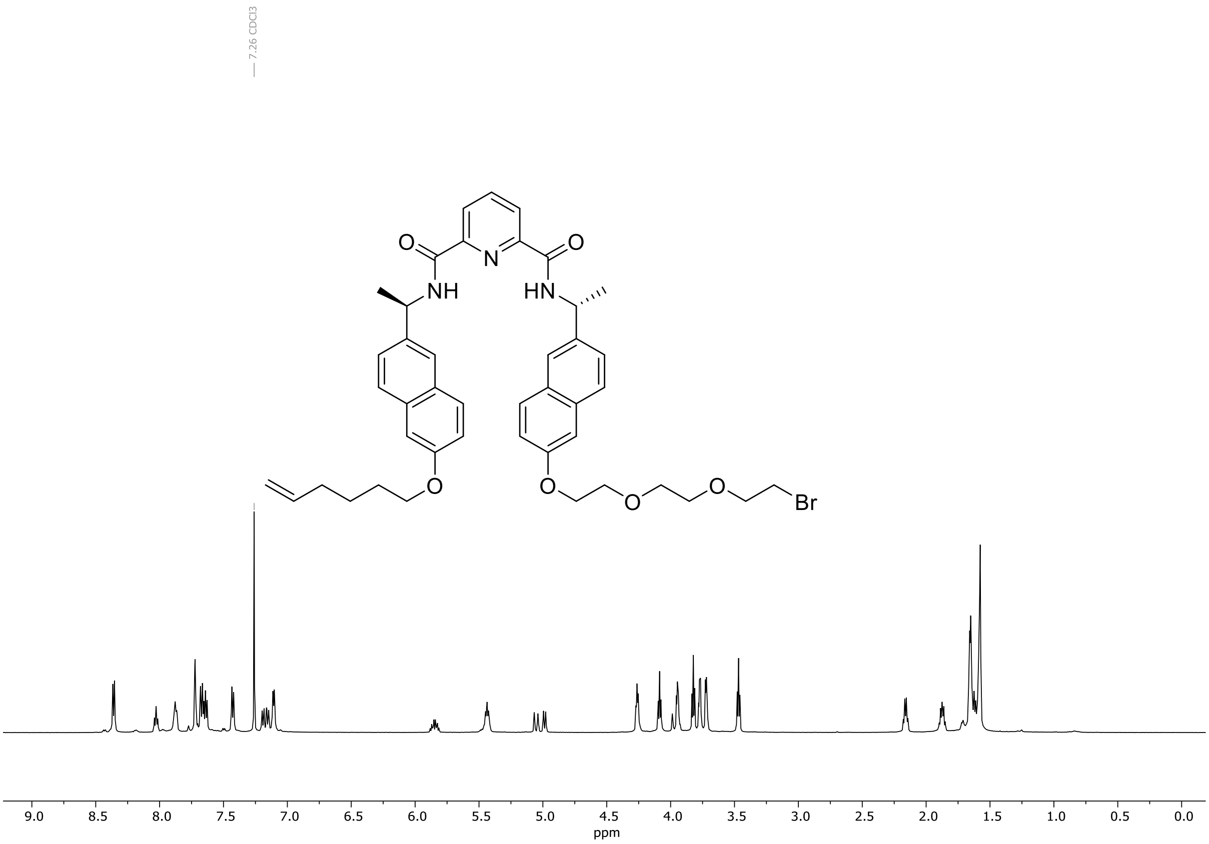

Spectrum 45. ^{1}H NMR spectrum (600 MHz, $CDCl_3$, 298 K) of compound **S12**.

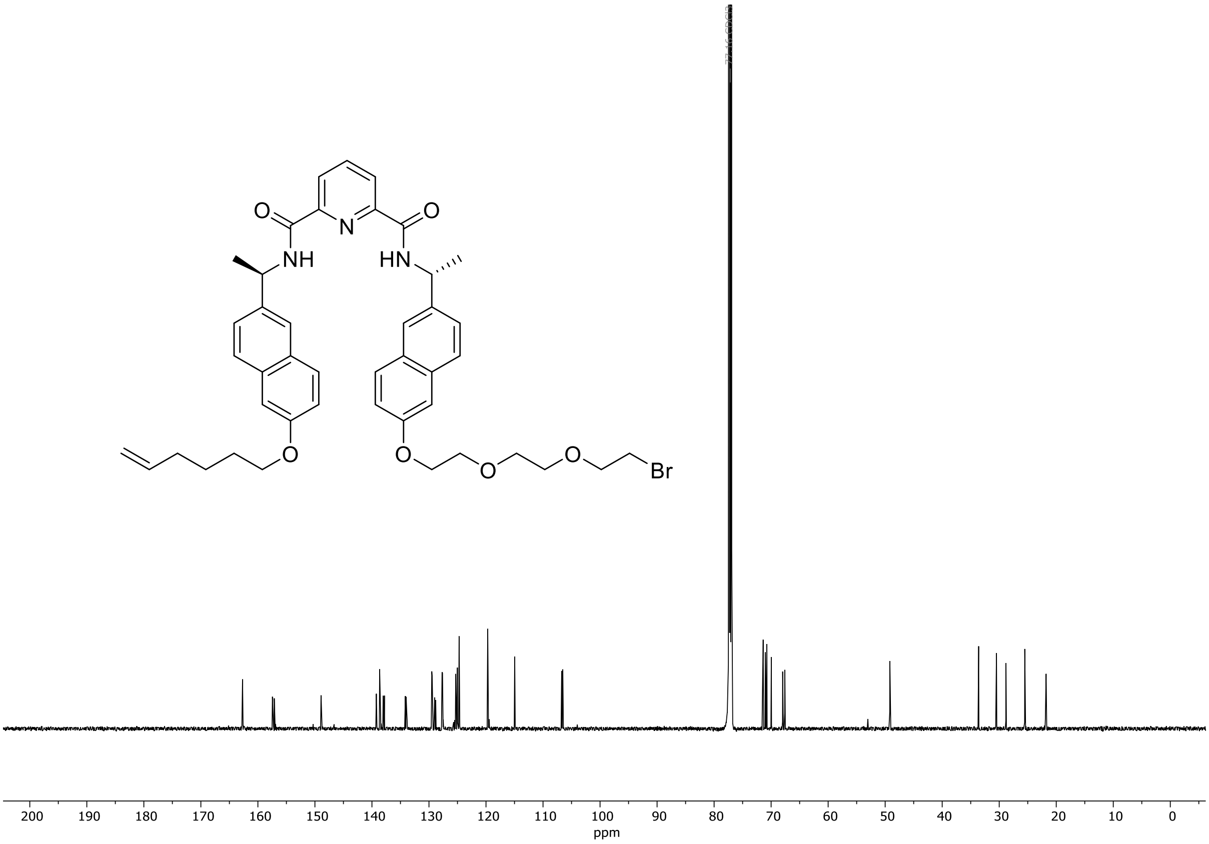

Spectrum 46. ^{13}C NMR spectrum (151 MHz, $CDCl_3$, 298 K) of compound **S12**.

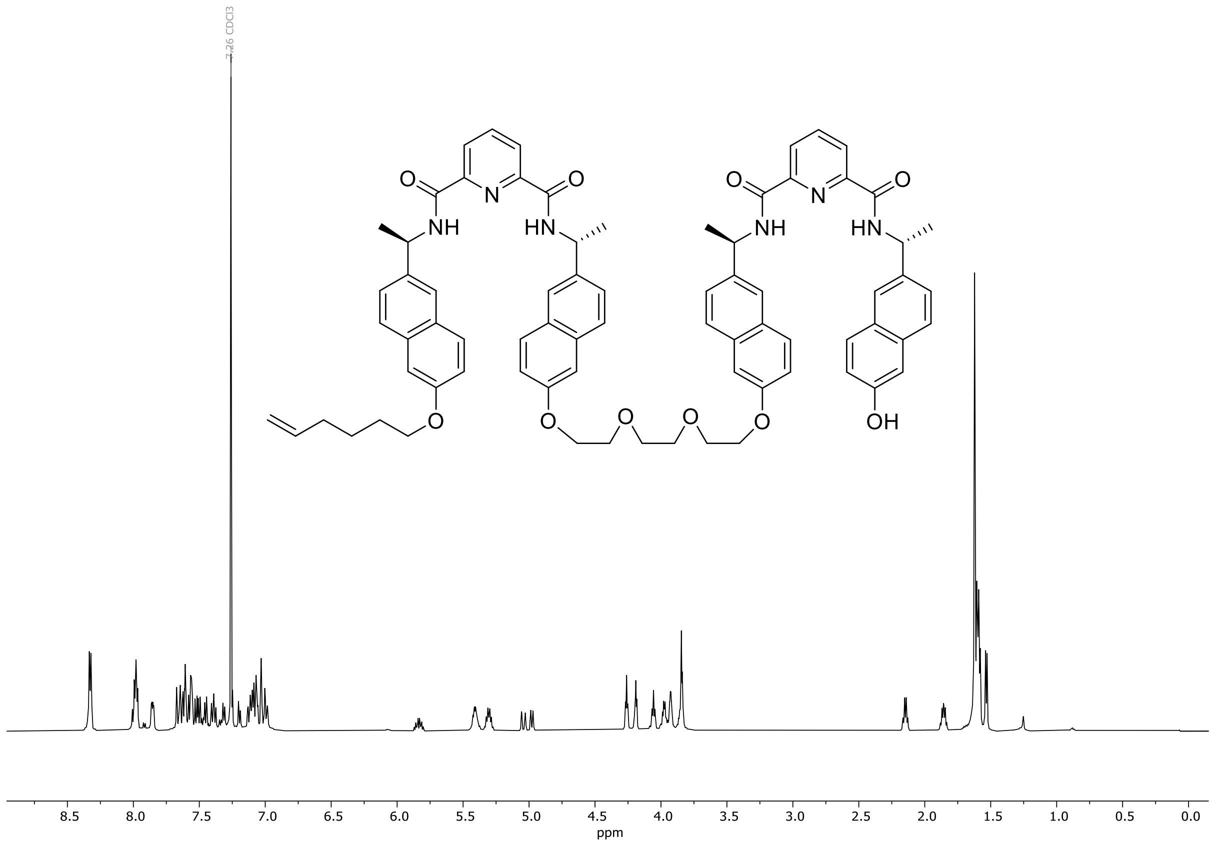

Spectrum 47. ^{1}H NMR spectrum (600 MHz, $CDCl_3$, 298 K) of compound **S13.**

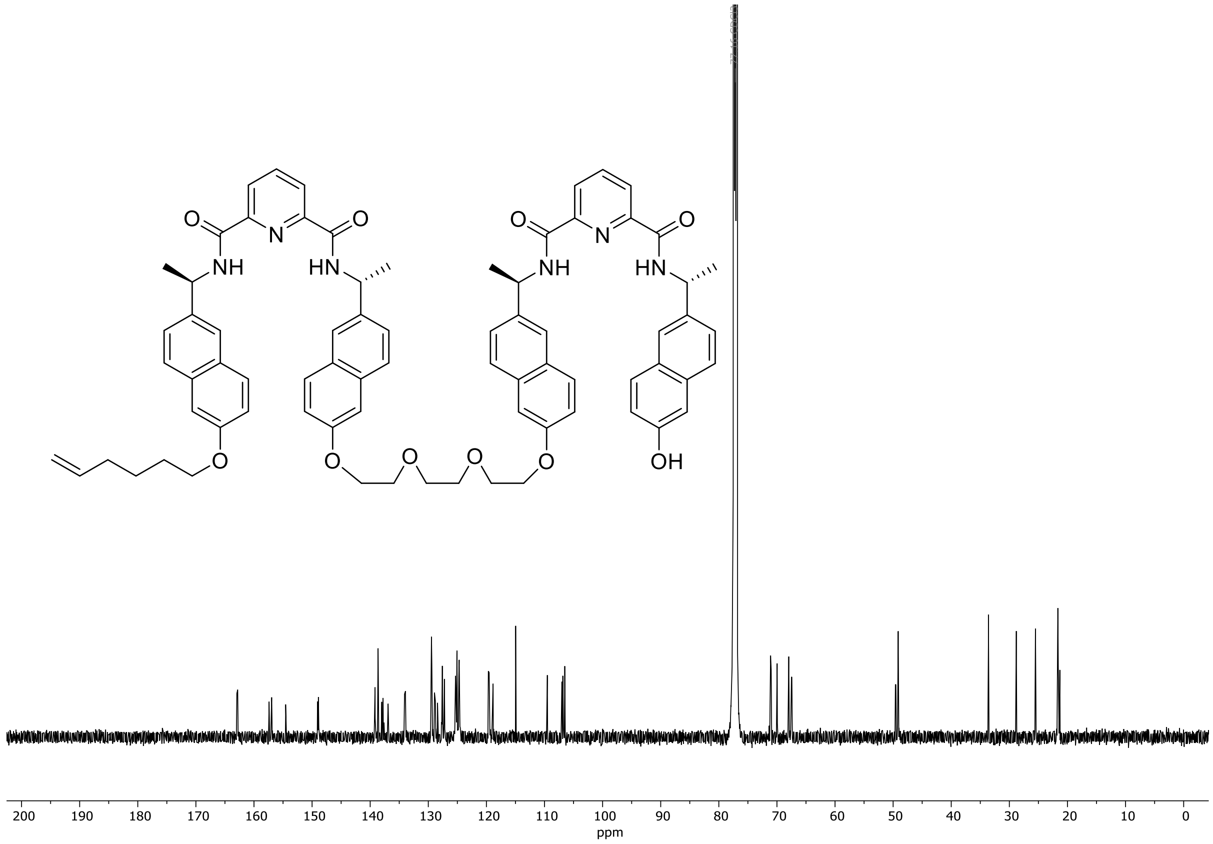

Spectrum 48. ^{13}C NMR spectrum (151 MHz, $CDCl_3$, 298 K) of compound **S13**.

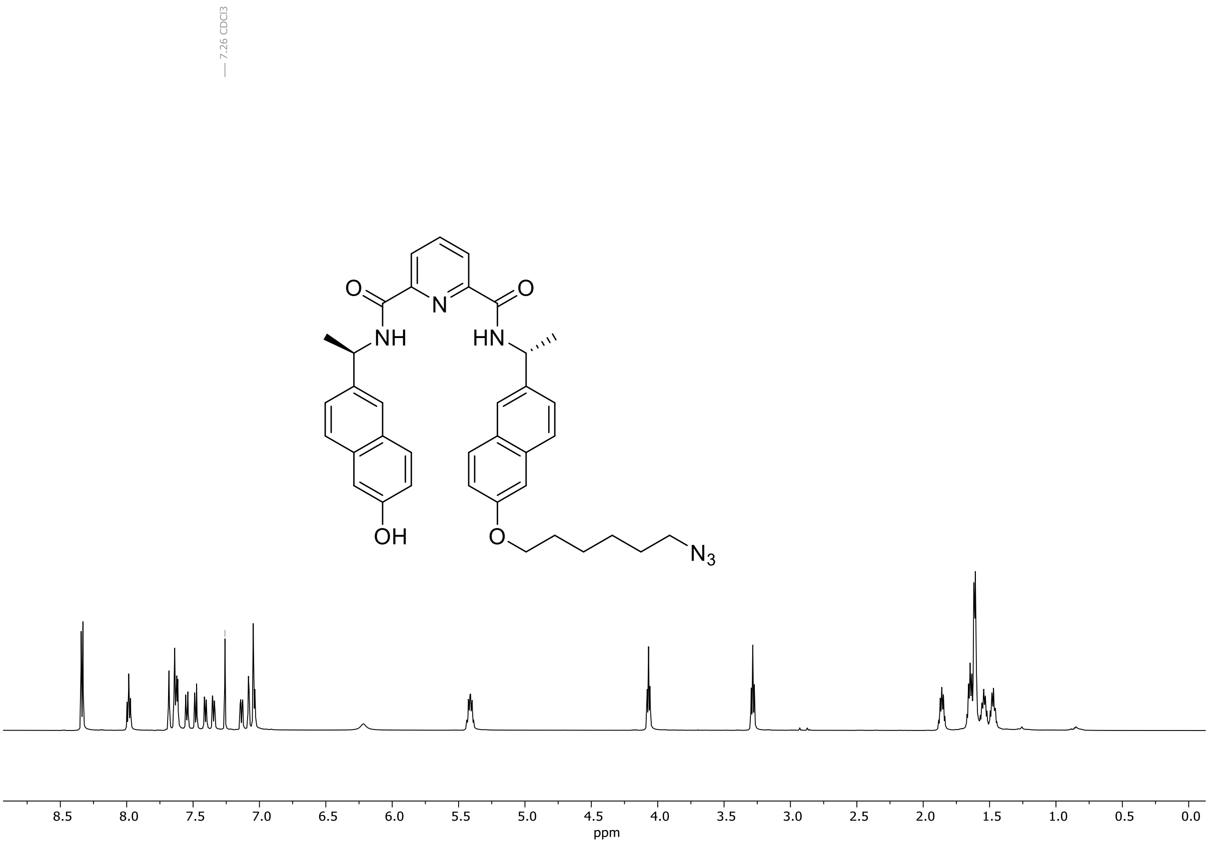

Spectrum 49. ^{1}H NMR spectrum (600 MHz, $CDCl_3$, 298 K) of compound **S14**.

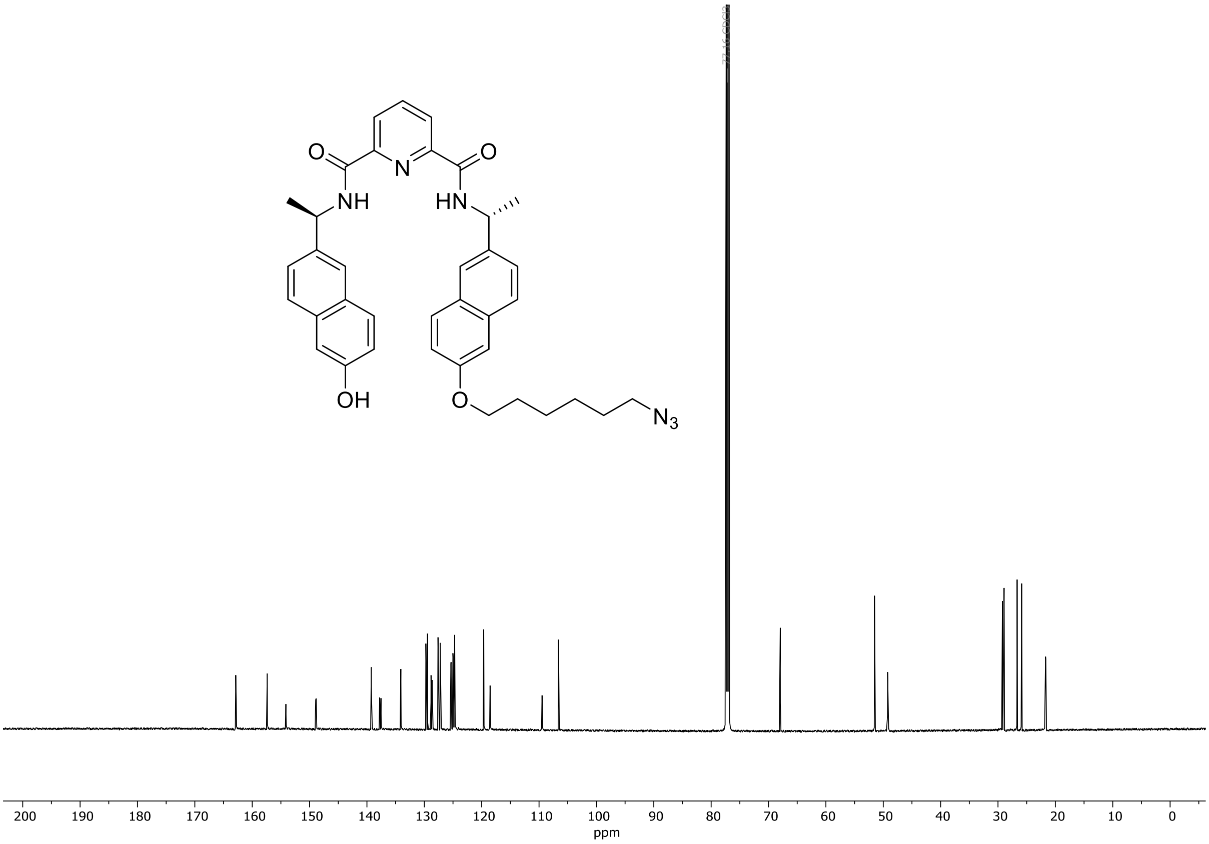

Spectrum 50. ^{13}C NMR spectrum (151 MHz, $CDCl_3$, 298 K) of compound **S14**.

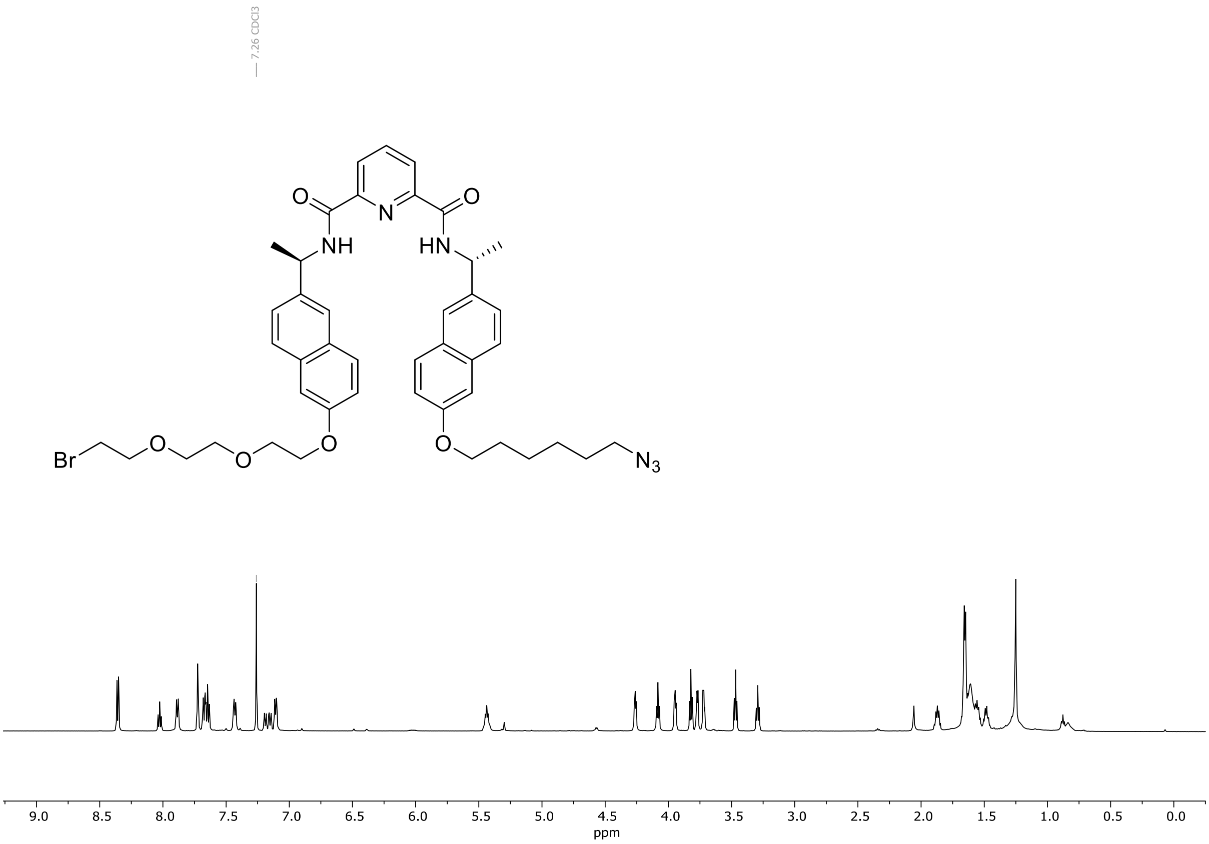

Spectrum 51. ^{1}H NMR spectrum (600 MHz, $CDCl_3$, 298 K) of compound **S15**.

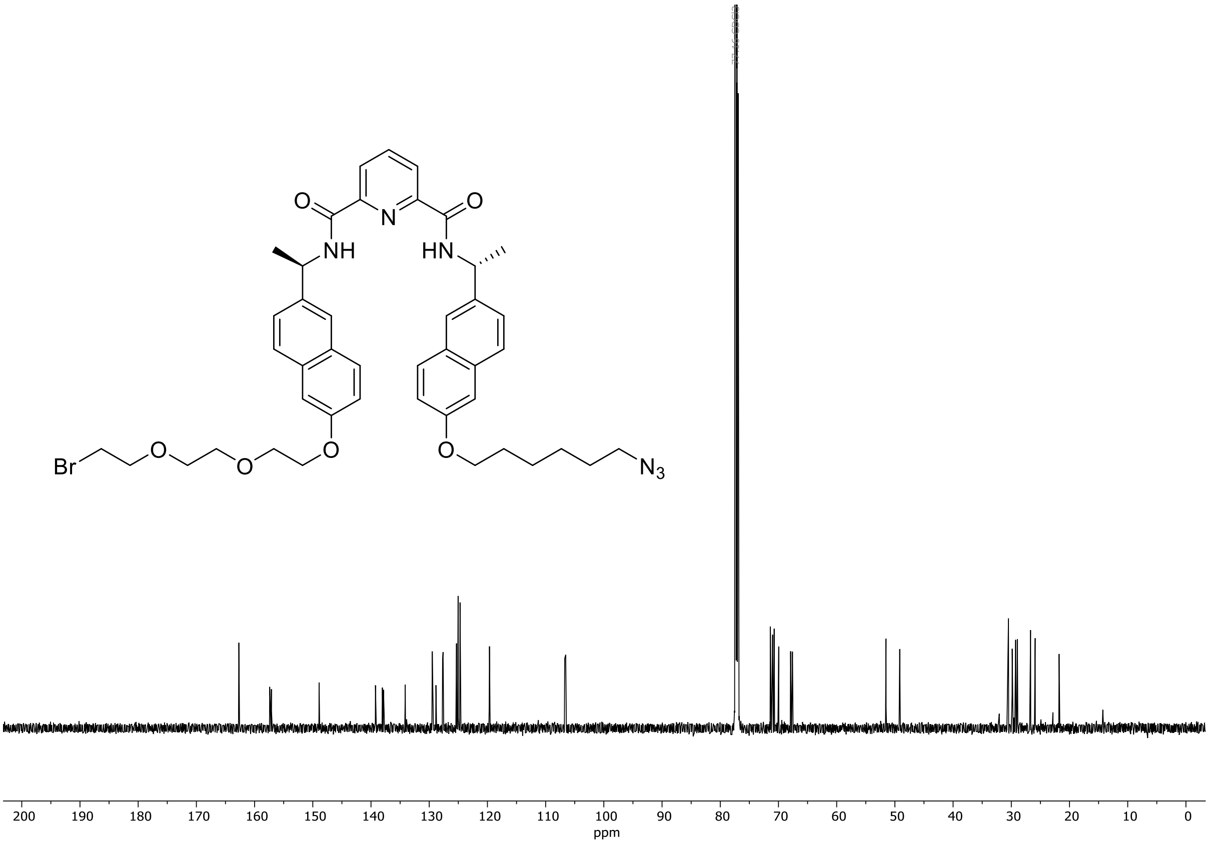

Spectrum 52. ^{13}C NMR spectrum (151 MHz, $CDCl_3$, 298 K) of compound **S15**.

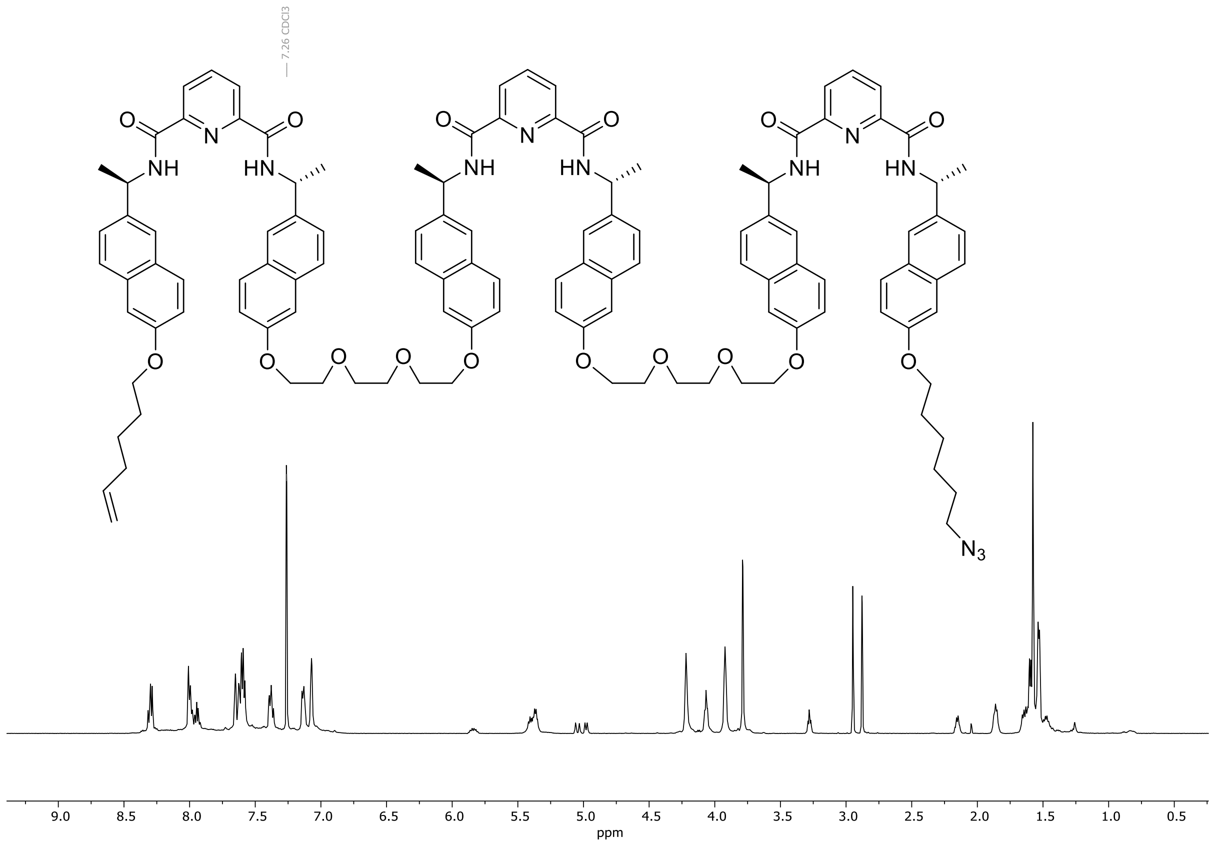

Spectrum 53. ^{1}H NMR spectrum (600 MHz, $CDCl_3$, 298 K) of compound **S16**.

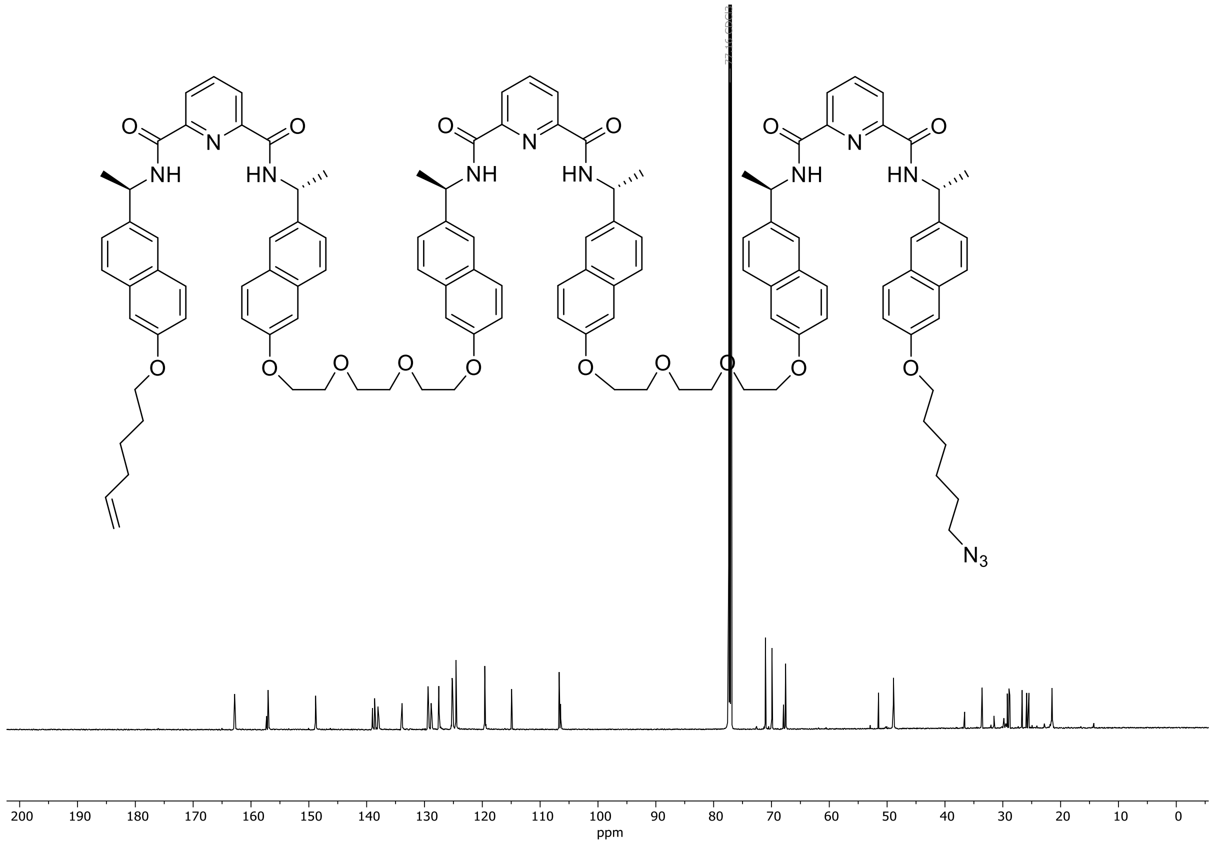

Spectrum 54. ^{13}C NMR spectrum (151 MHz, $CDCl_3$, 298 K) of compound **S16**.

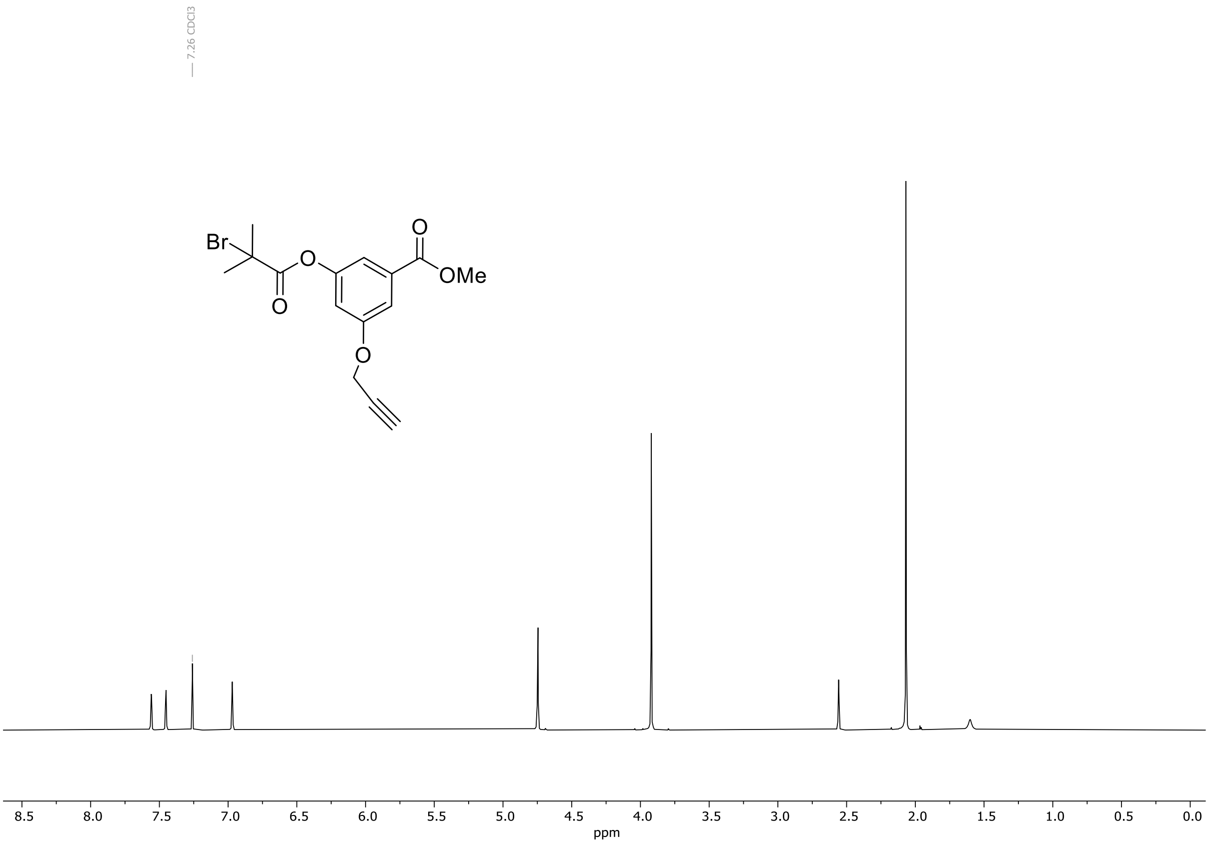

Spectrum 55. ^{1}H NMR spectrum (600 MHz, $CDCl_3$, 298 K) of polymer **S18**.

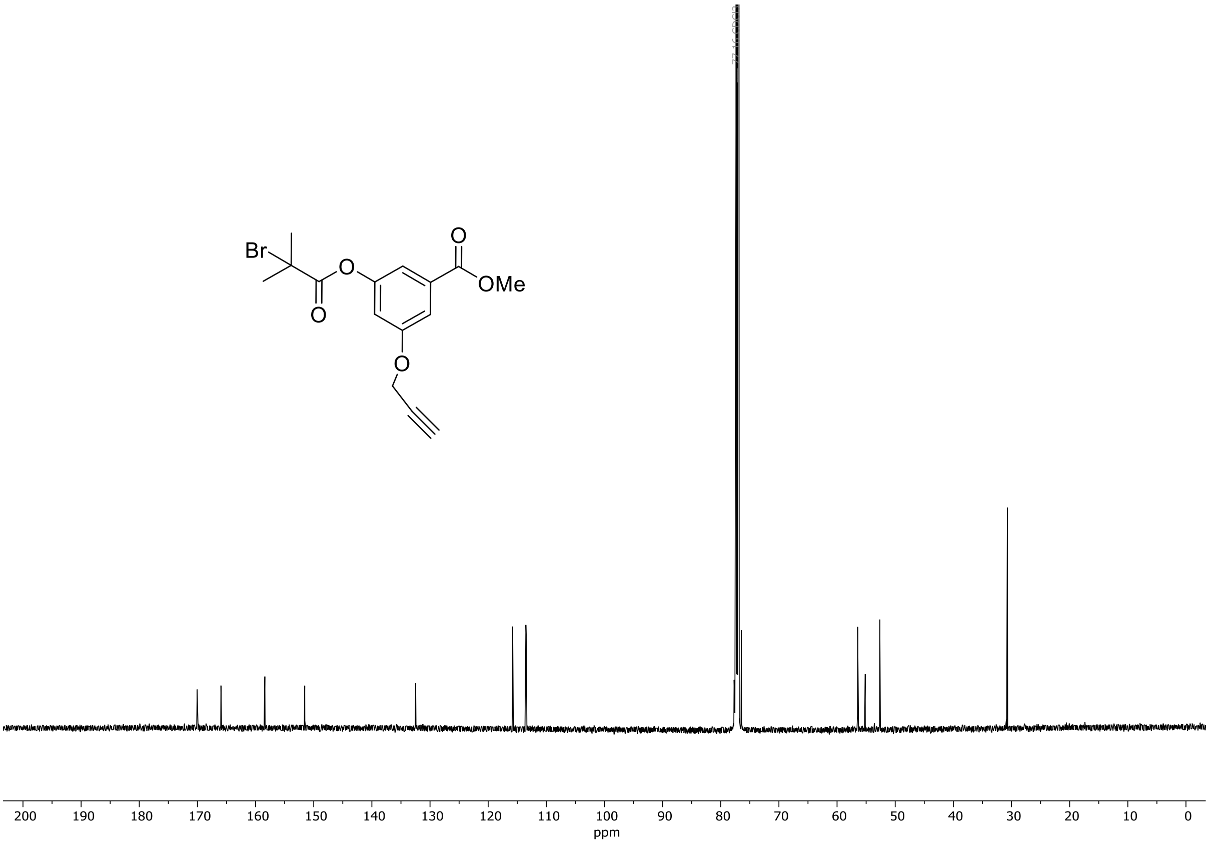

Spectrum 56. ^{13}C NMR spectrum (151 MHz, $CDCl_3$, 298 K) of compound **S18**.

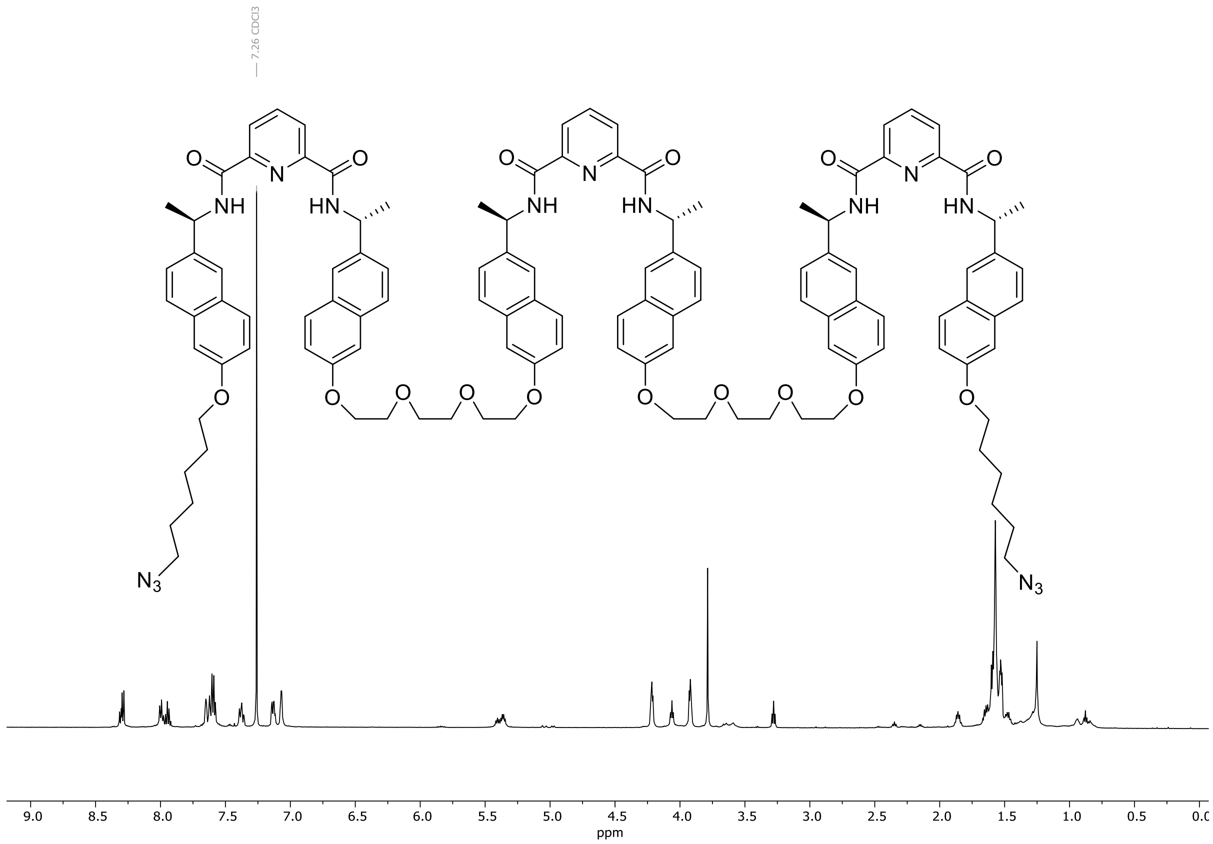

Spectrum 57. ^{1}H NMR spectrum (600 MHz, $CDCl_3$, 298 K) of compound **S19**.

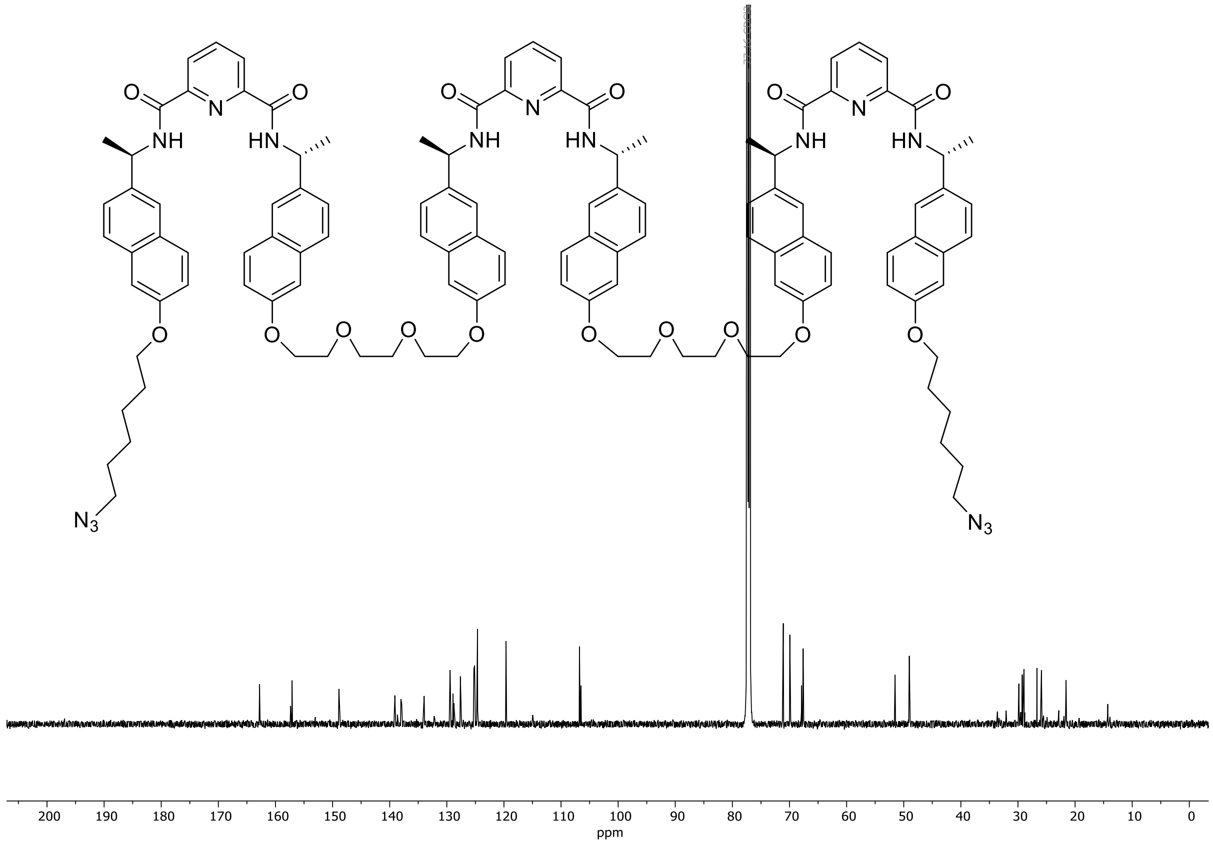

Spectrum 58. ^{13}C NMR spectrum (151 MHz, $CDCl_3$, 298 K) of compound **S19**.

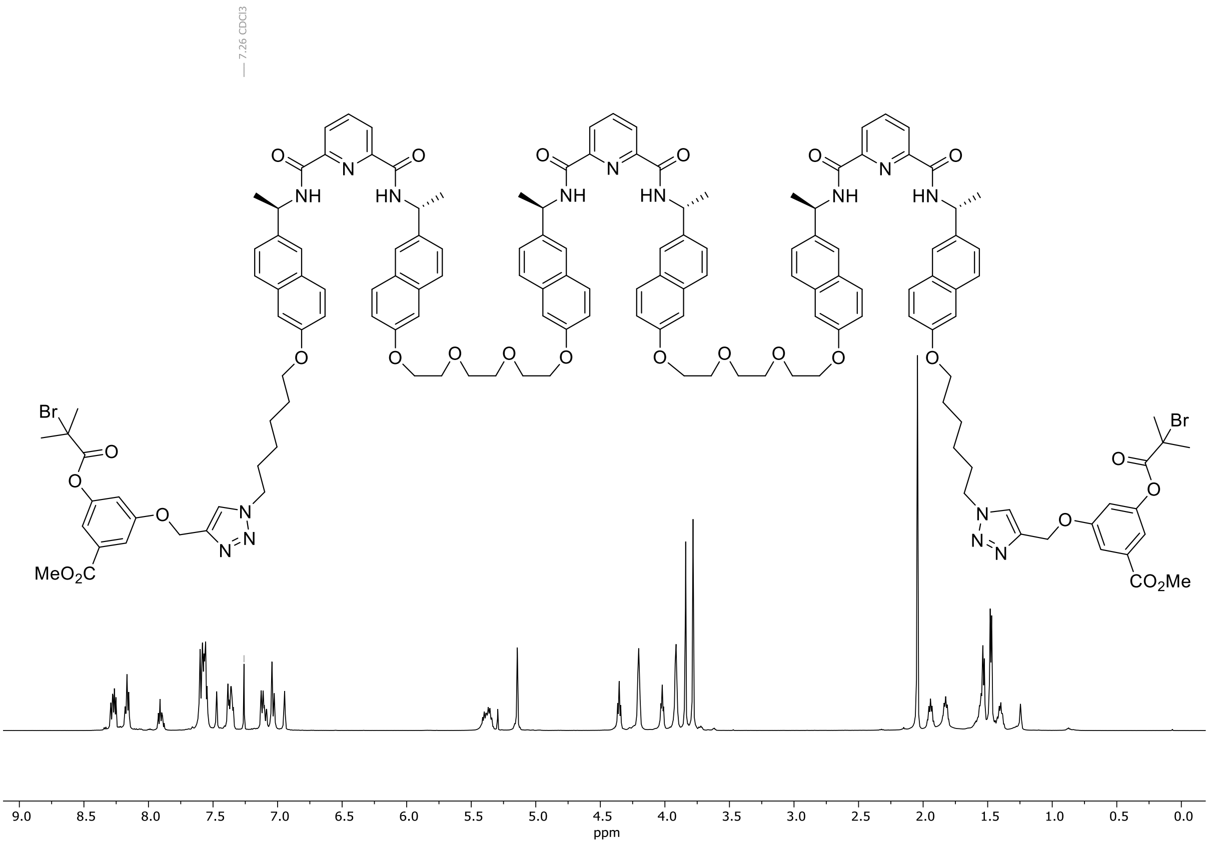

Spectrum 59. ^{1}H NMR spectrum (600 MHz, $CDCl_3$, 298 K) of compound **S20**.

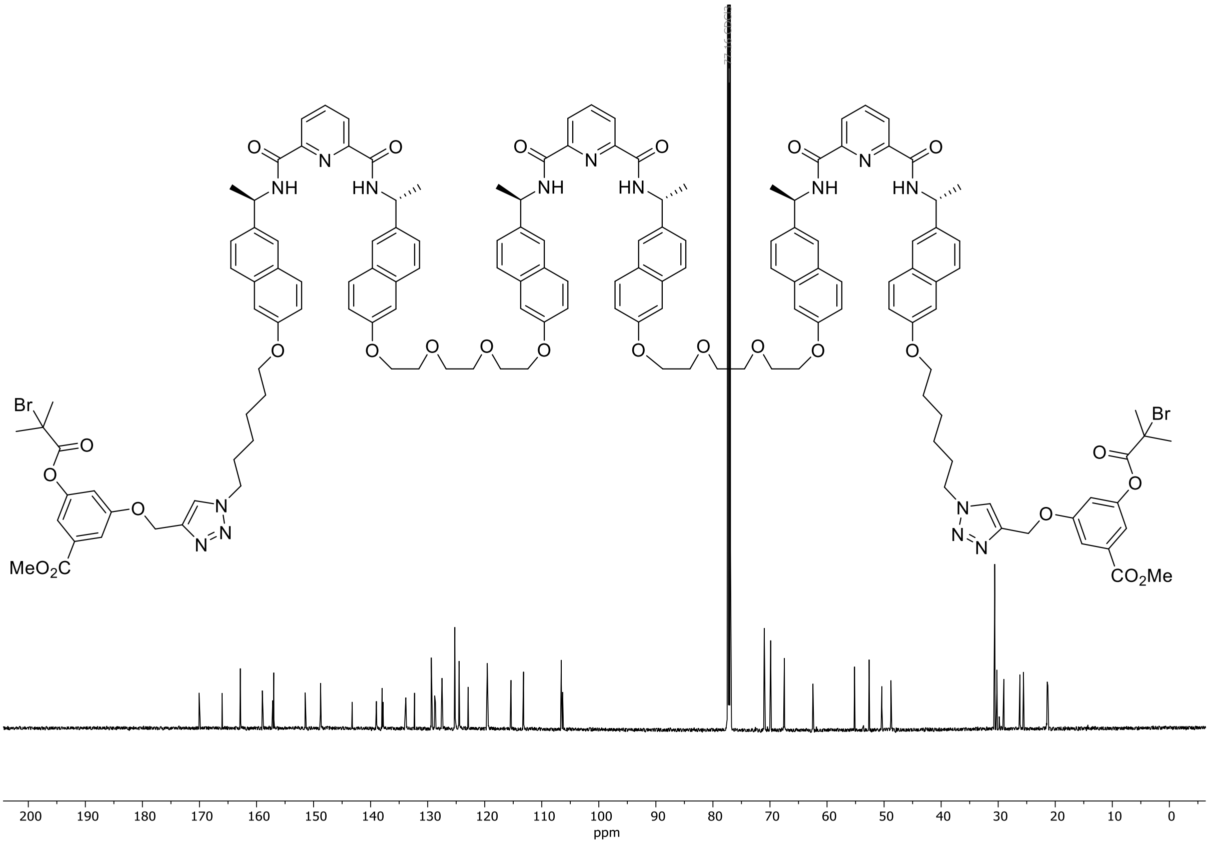

Spectrum 60. ^{13}C NMR spectrum (151 MHz, $CDCl_3$, 298 K) of compound **S20**.

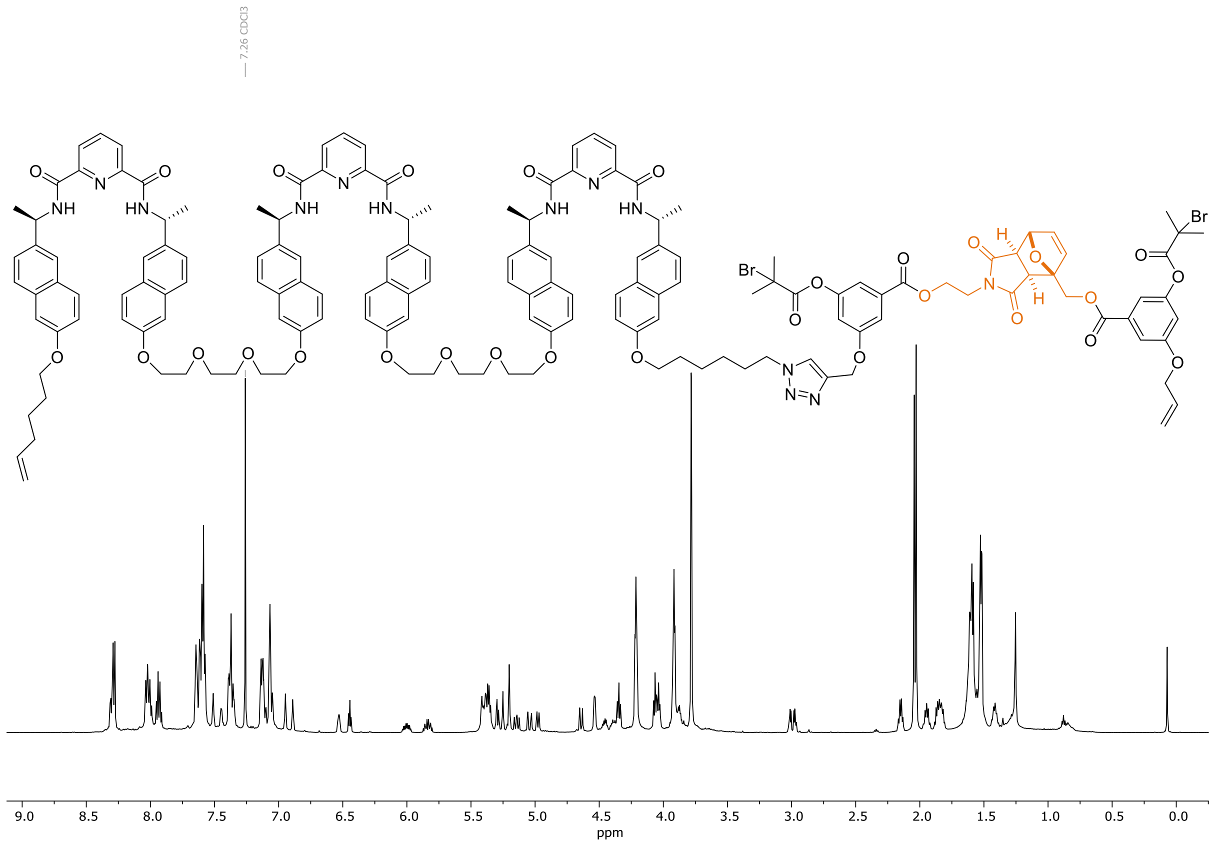

Spectrum 61. ^{1}H NMR spectrum (600 MHz, $CDCl_3$, 298 K) of compound **S21**.

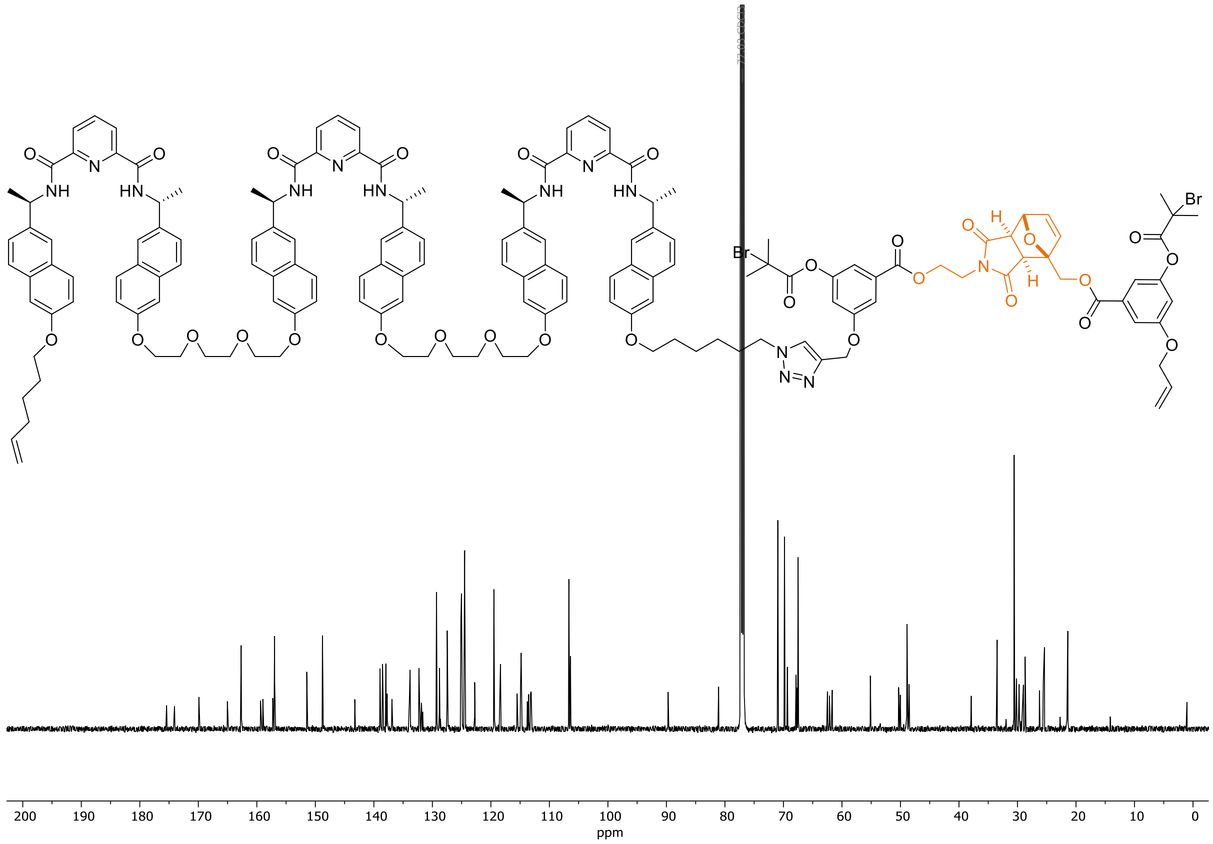

Spectrum 62. ^{13}C NMR spectrum (151 MHz, $CDCl_3$, 298 K) of compound **S21**.

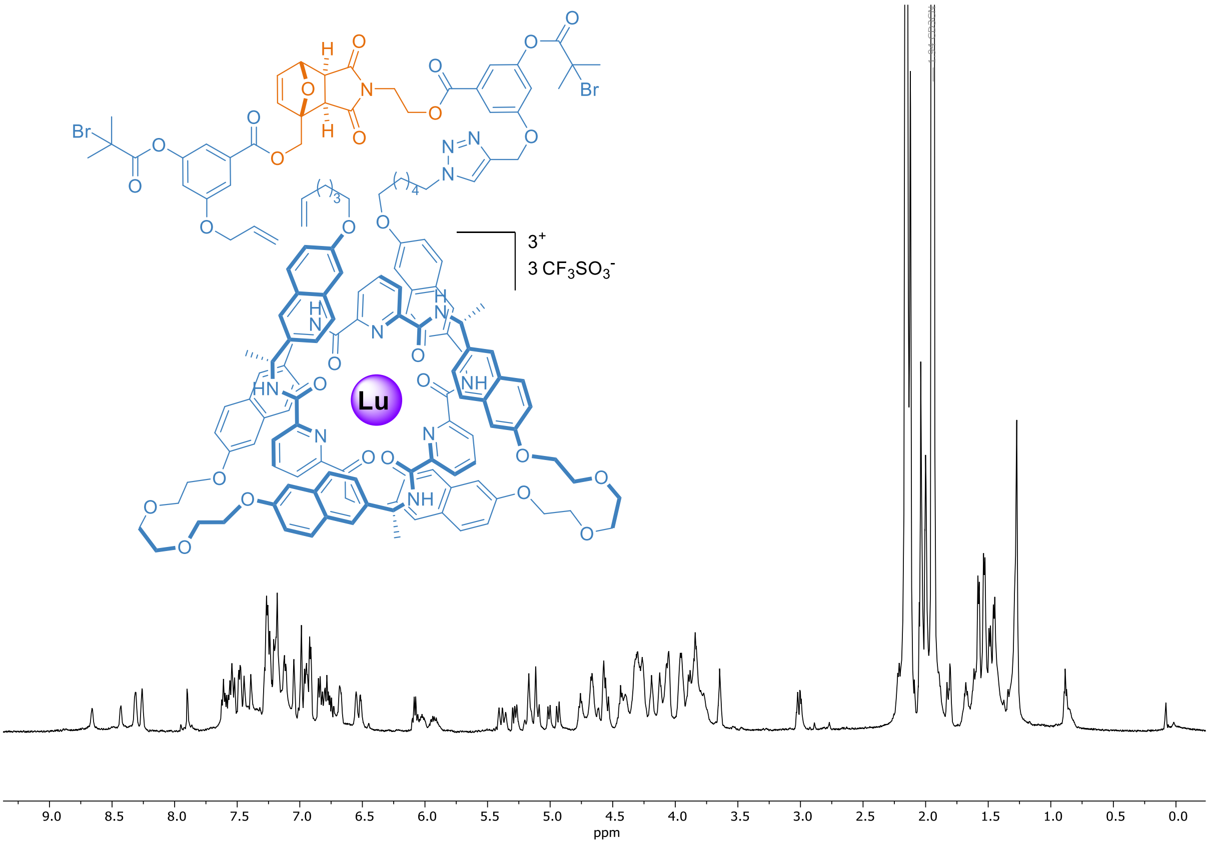

Spectrum 63. ^{1}H NMR spectrum (600 MHz, CD_3CN, 298 K) of overhand knot **S22**.

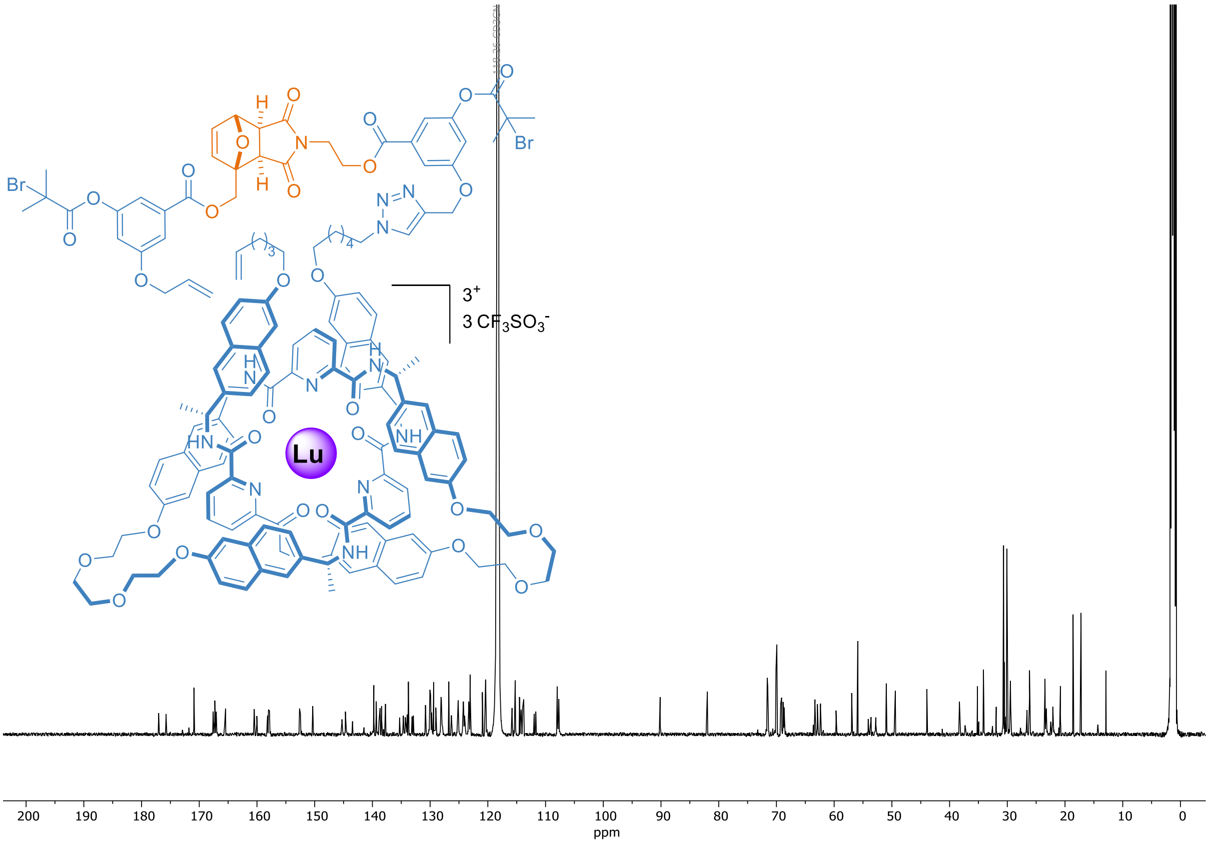

Spectrum 64. ^{13}C NMR spectrum (151 MHz, CD_3CN, 298 K) of overhand knot **S22**.

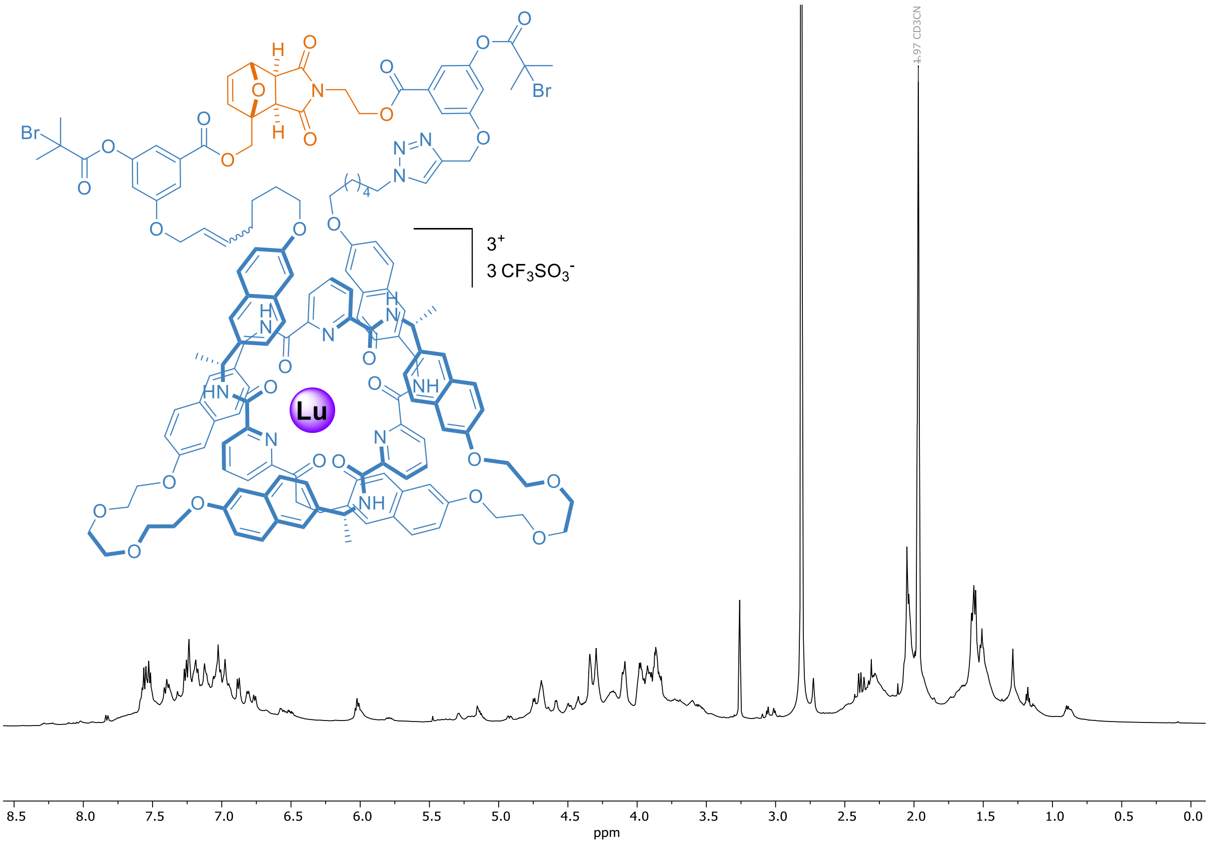

Spectrum 65. ^{1}H NMR spectrum (600 MHz, CD_3CN/MeOD 10:1, 298 K) of gated trefoil knot Lu-3_1 **S23**.

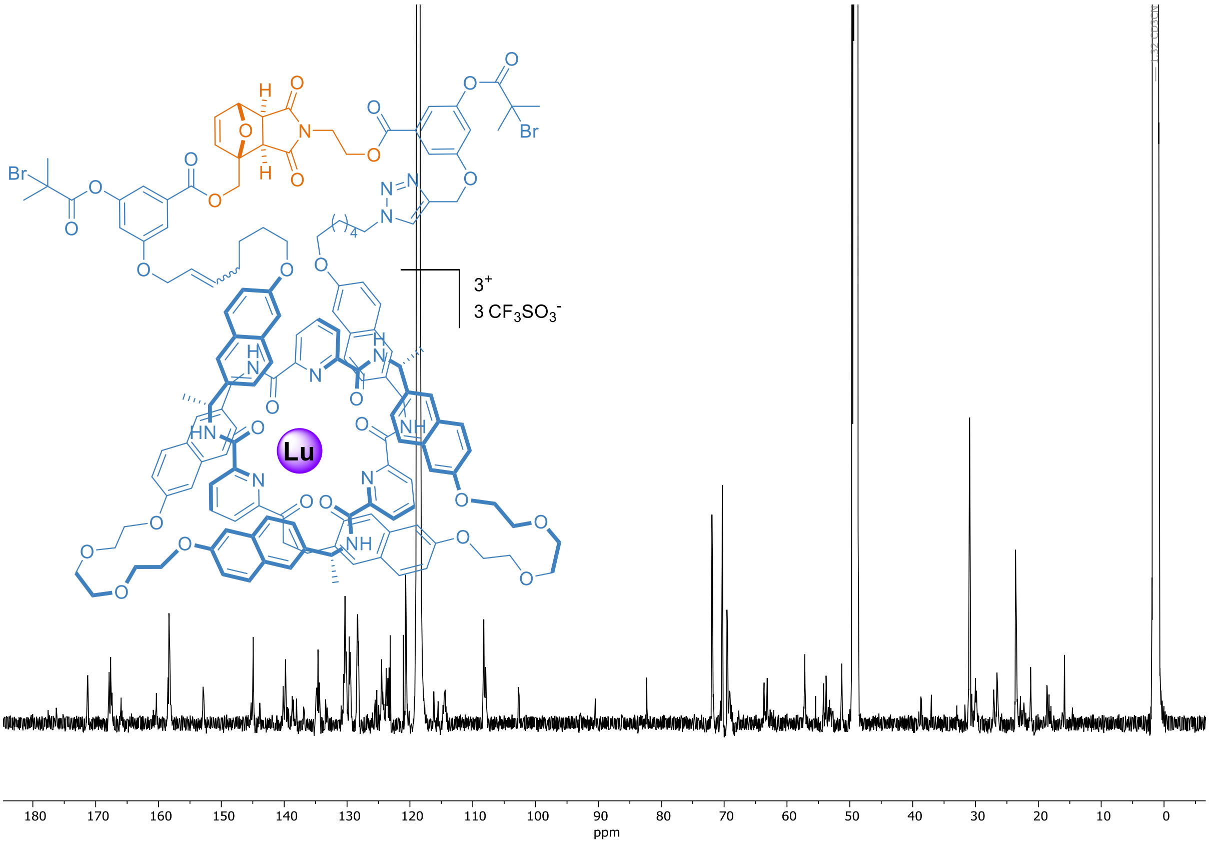

Spectrum 66. ^{13}C NMR spectrum (151 MHz, CD_3CN/MeOD 10:1, 298 K) of gated trefoil knot Lu-3_1 **S23**.

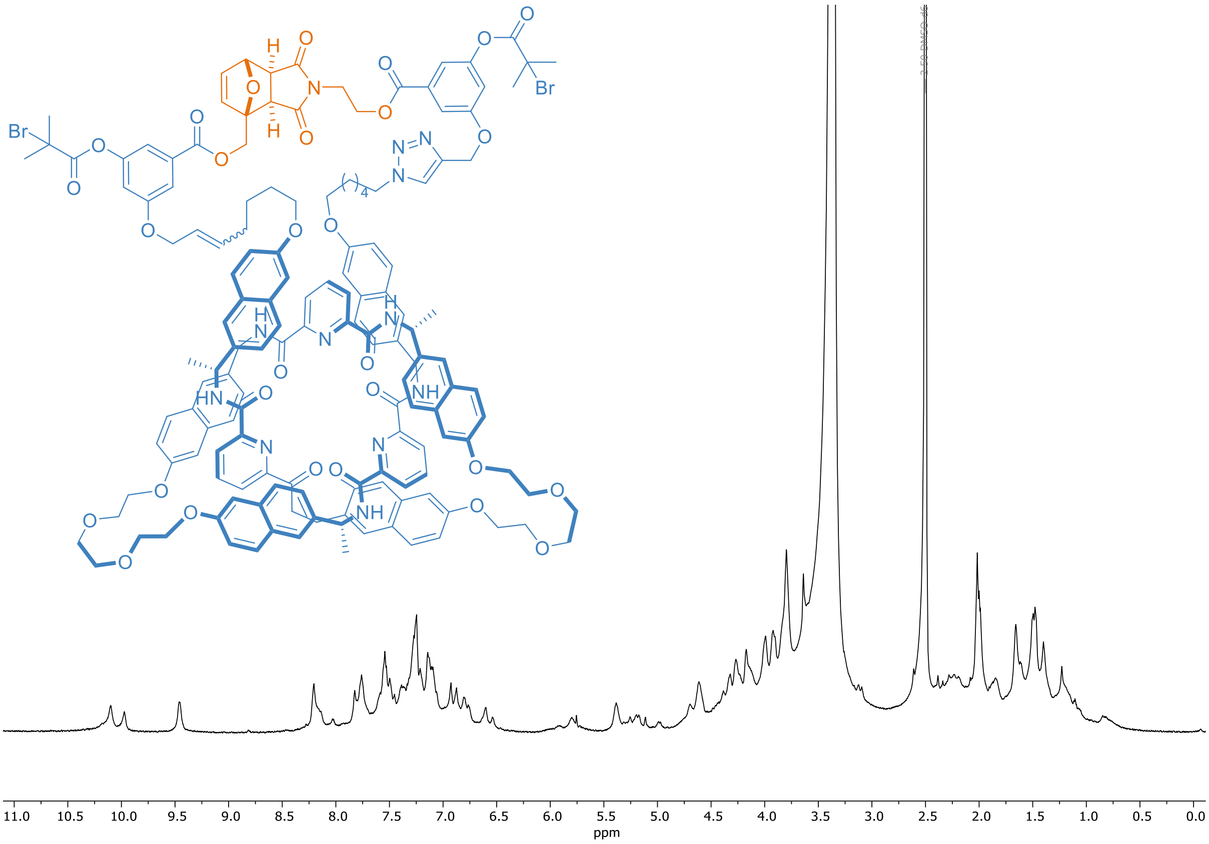

Spectrum 67. ^{1}H NMR spectrum (600 MHz, DMSO-d_6, 298 K) of gated organic trefoil knot **S24**.

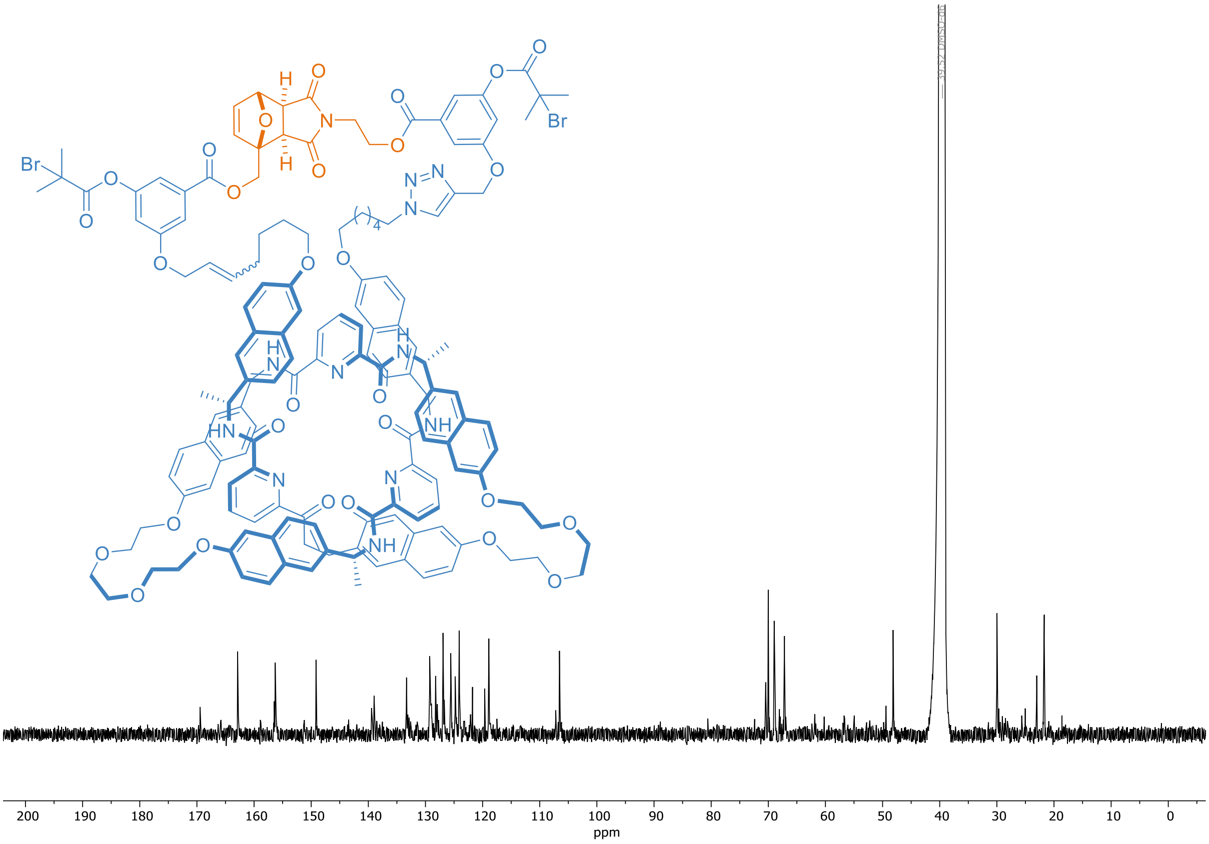

Spectrum 68. ^{13}C NMR spectrum (151 MHz, DMSO-d_6, 298 K) of gated organic trefoil knot **S24**.

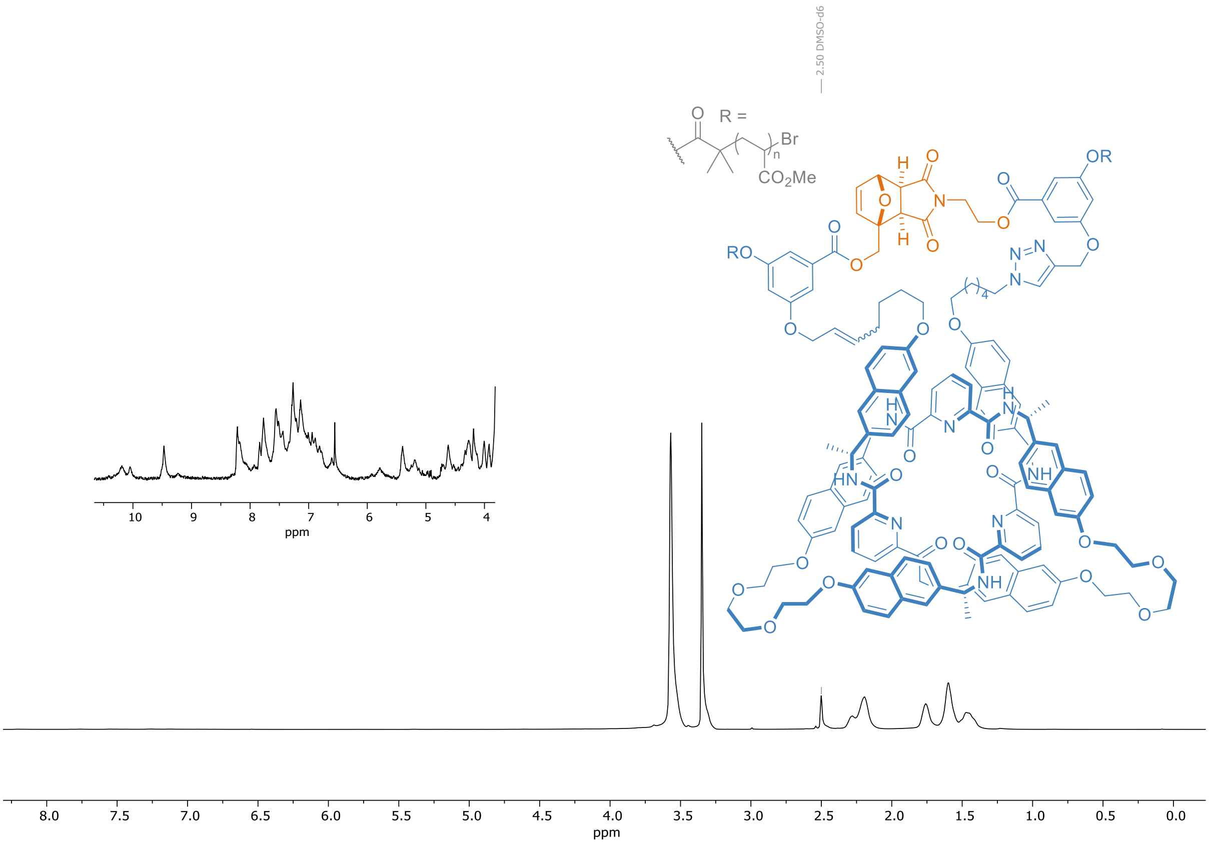

Spectrum 69. ^{1}H NMR spectrum (600 MHz, DMSO-d_6, 298 K) of gated knot polymer **1**.

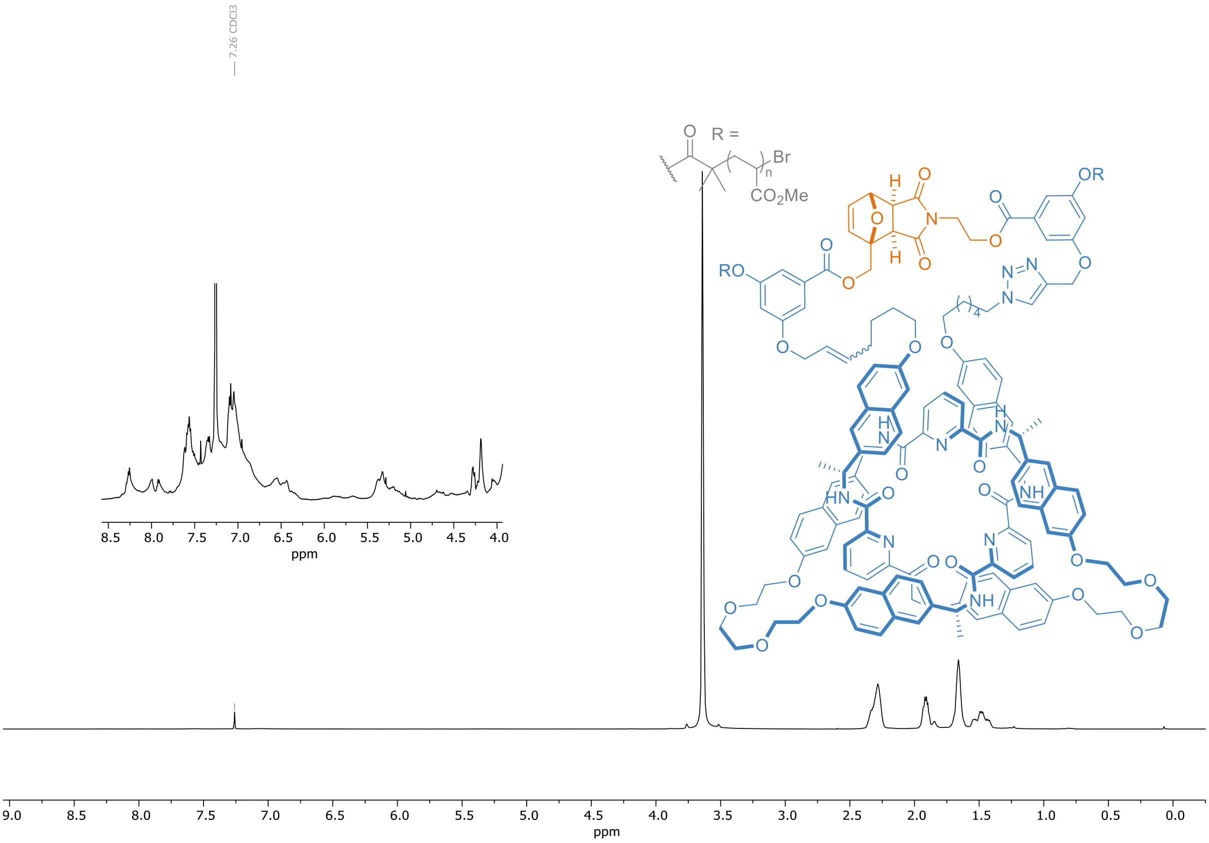

Spectrum 70. ^{1}H NMR spectrum (600 MHz, $CDCl_3$, 298 K) of gated knot polymer **1**.

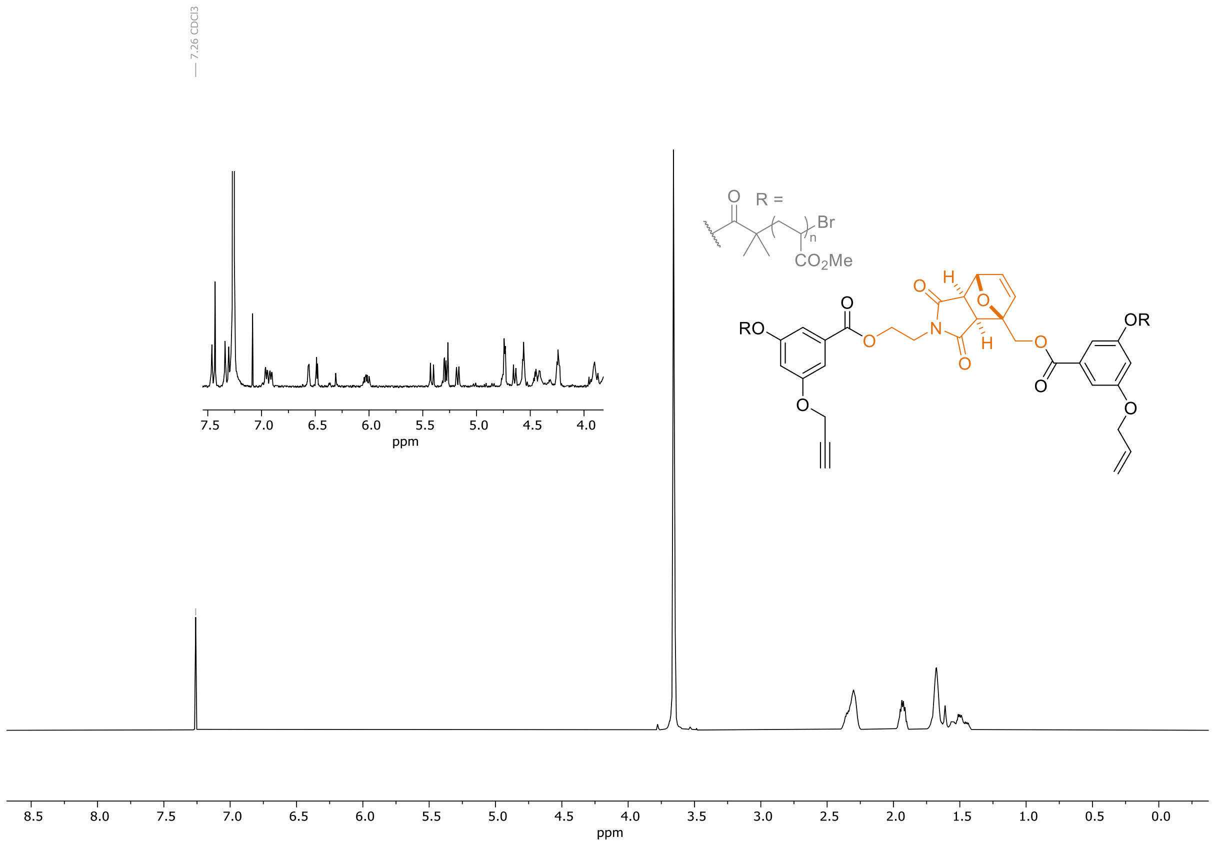

Spectrum 71. ^{1}H NMR spectrum (600 MHz, $CDCl_3$, 298 K) of gate polymer **2**.

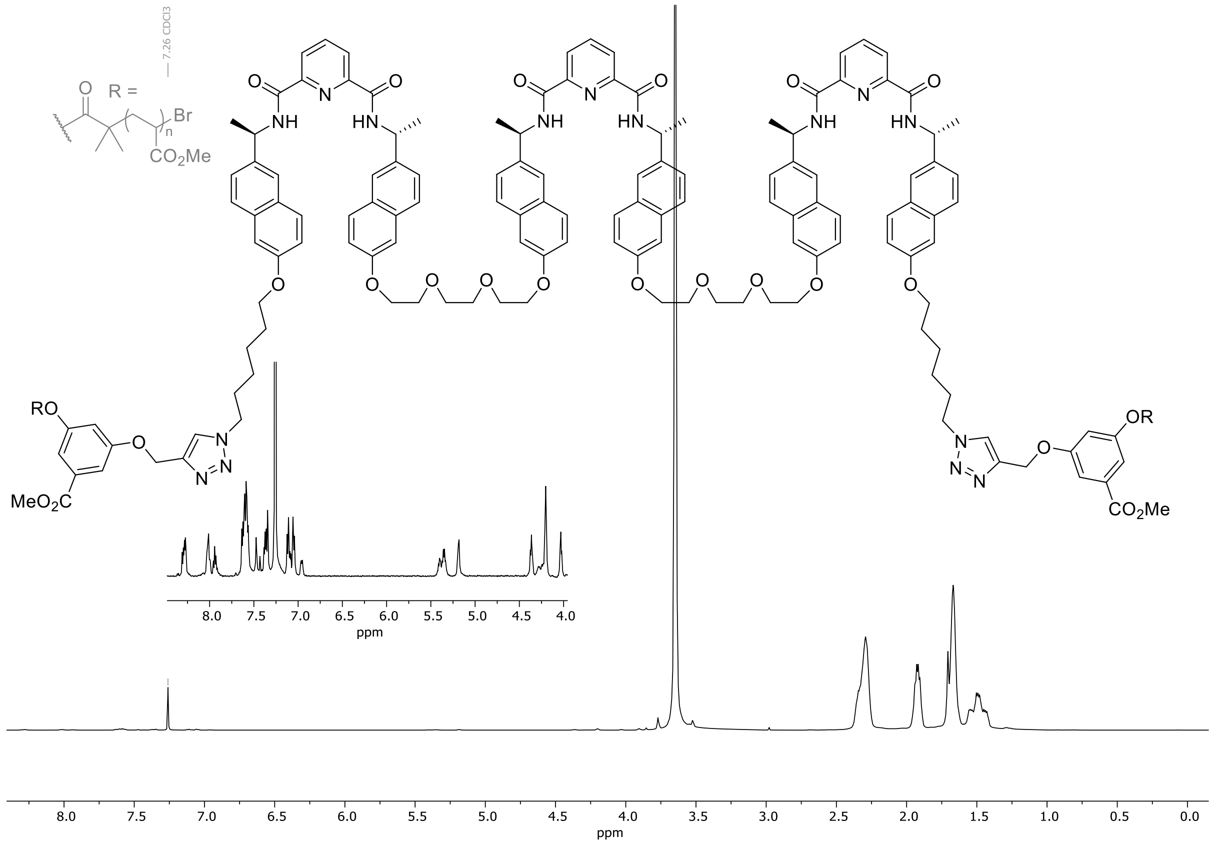

Spectrum 72. ^{1}H NMR spectrum (600 MHz, $CDCl_3$, 298 K) of linear ligand polymer **3**.

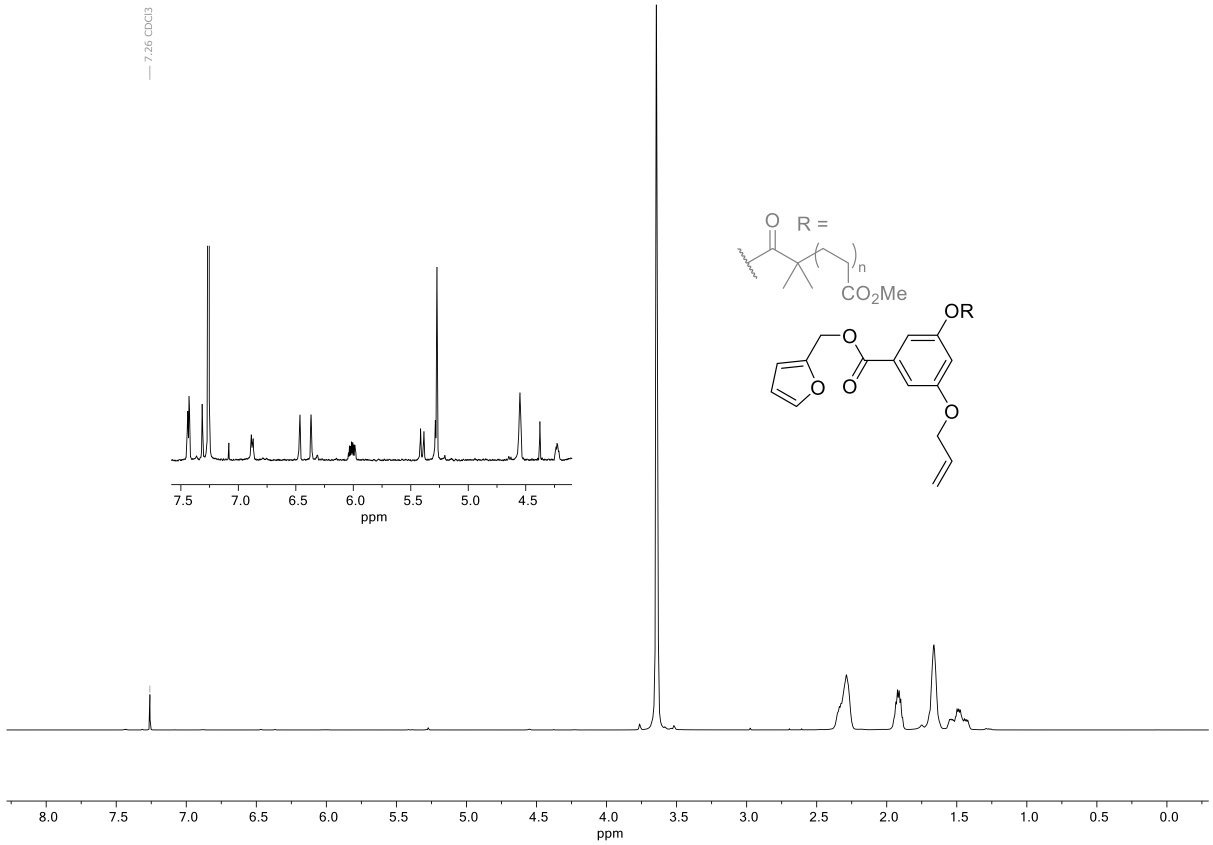

Spectrum 73. ^{1}H NMR spectrum (600 MHz, $CDCl_3$, 298 K) of reference polymer **S30**.

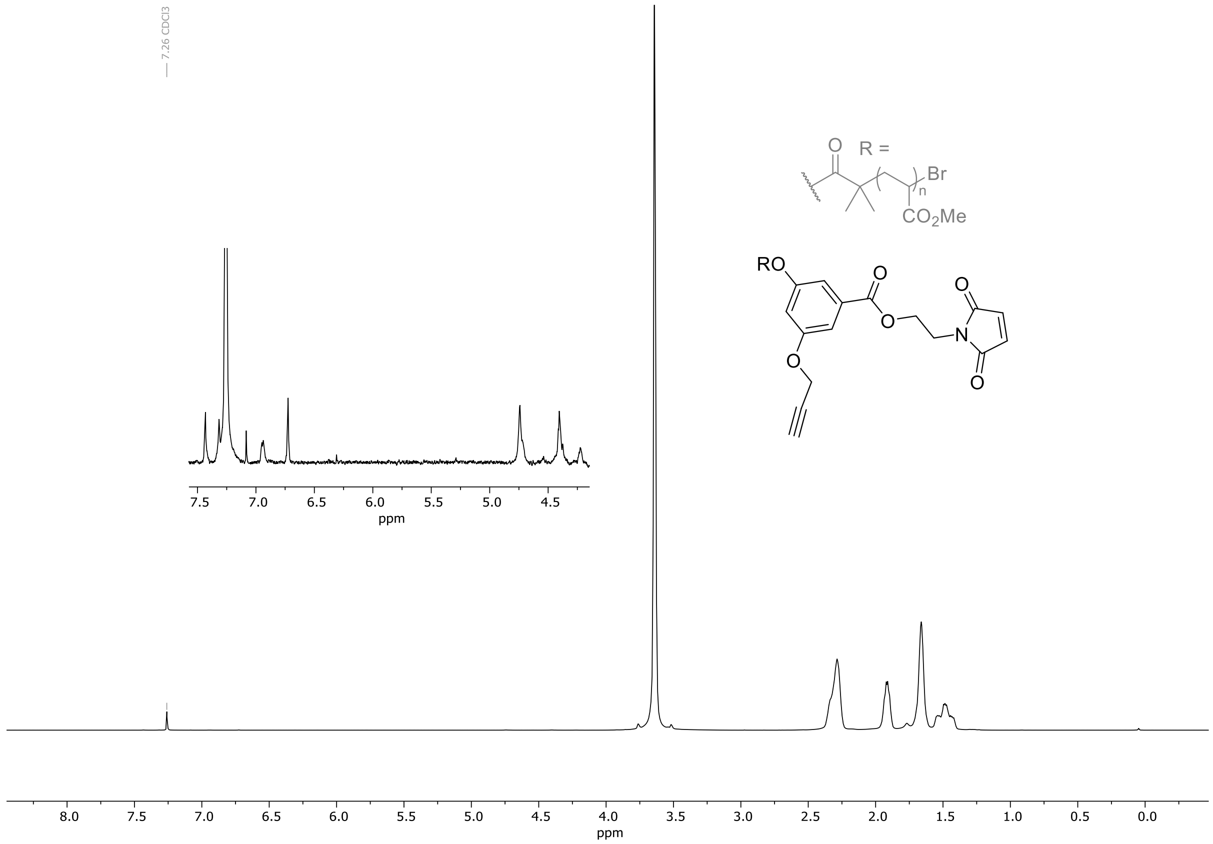

Spectrum 74. ^{1}H NMR spectrum (600 MHz, $CDCl_3$, 298 K) of reference polymer **S31**.

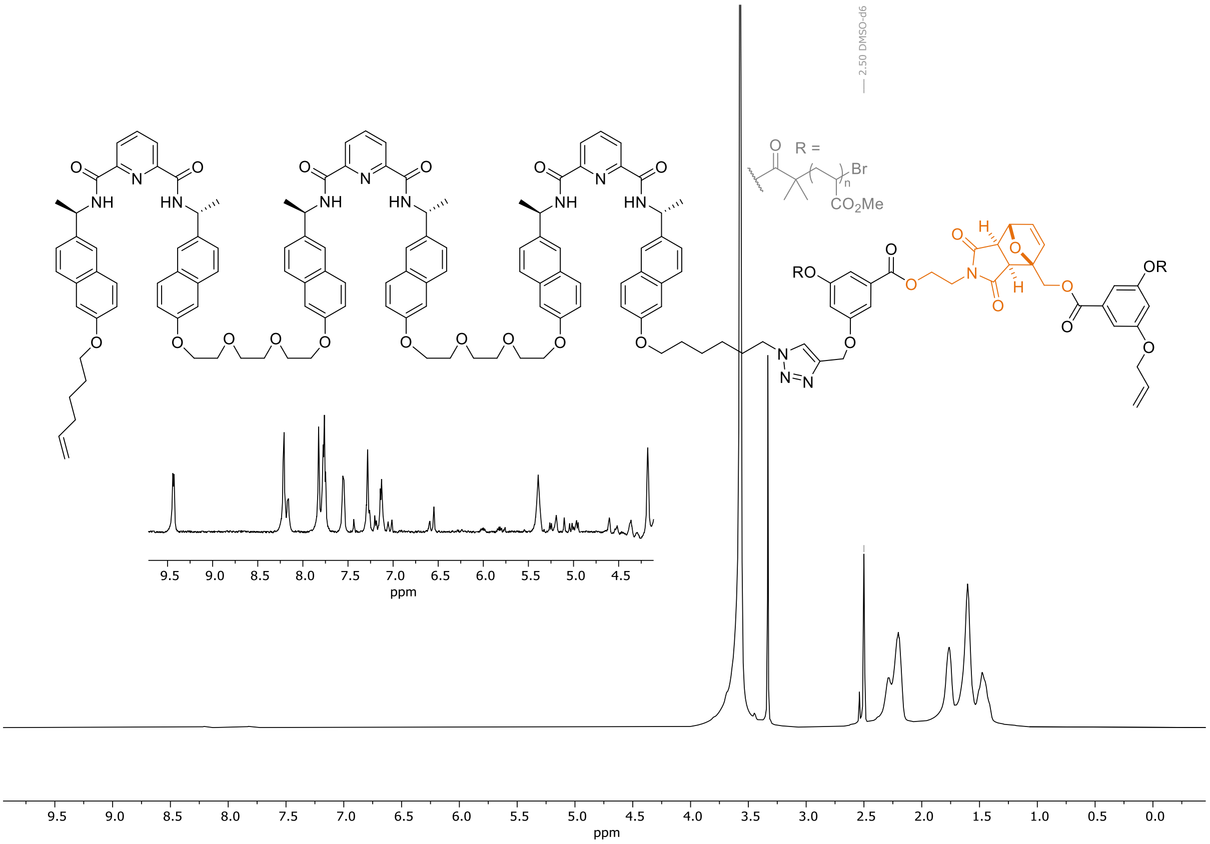

Spectrum 75. ^{1}H NMR spectrum (600 MHz, DMSO-d_6, 298 K) of reference polymer **S32**.

9 CoGEF Calculations

9.1 General procedure

CoGEF calculations were performed on Spartan ’14 (MMFF) and Spartan ‘18/’20 (DFT) following Beyer’s method.[7] The structure of each compound was built in Spartan ’14 and minimized using molecular mechanics (MMFF). The distance between the anchor atoms (highlighted with a pink disk) ester was constrained and increased by increments of 0.2Å to 1Å depending on the compound. At each step, the energy was minimized by molecular mechanics (MMFF) then DFT (B3LYP/6-31G*) in vacuum. The relative energy of each intermediate was determined by setting the energy of the initial state at 0 kJ/mol. F_{max} values were determined from the slope of the final 40% of the energy/elongation curve (i.e. from E_{max} to 0.6 E_{max} or nearest point).

9.2 Gate model

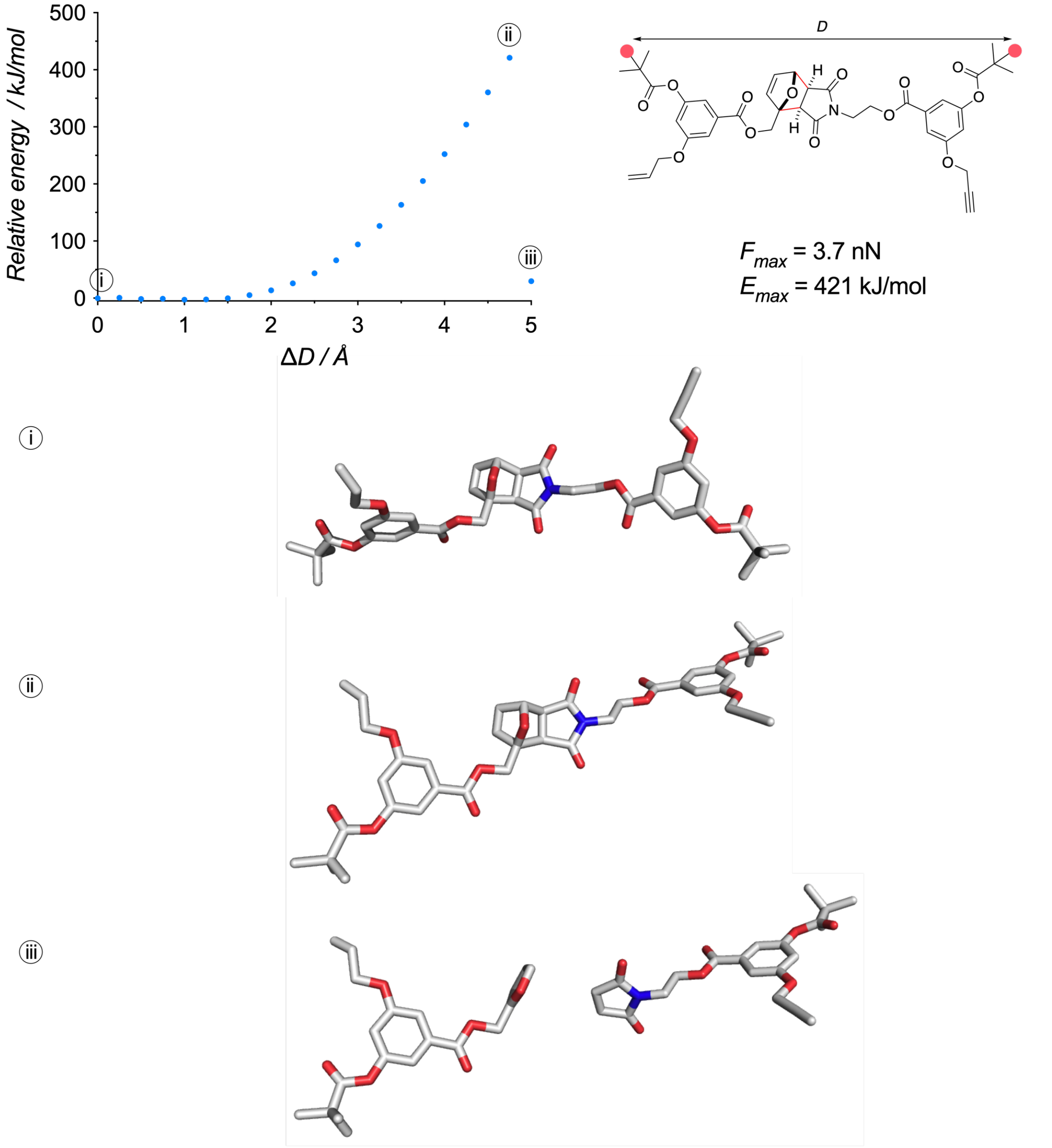

Figure 4. Evolution of energy of the gate model upon simulated elongation (CoGEF, DFT B3LYP/6-31G*) and structures at E_0 (i) E_{max} (ii), and scission (iii). F_{max} determined from the slope of the final 40% of the energy/elongation curve. Scissile bonds shown in red in the top structure.

9.3 Ligand model 1

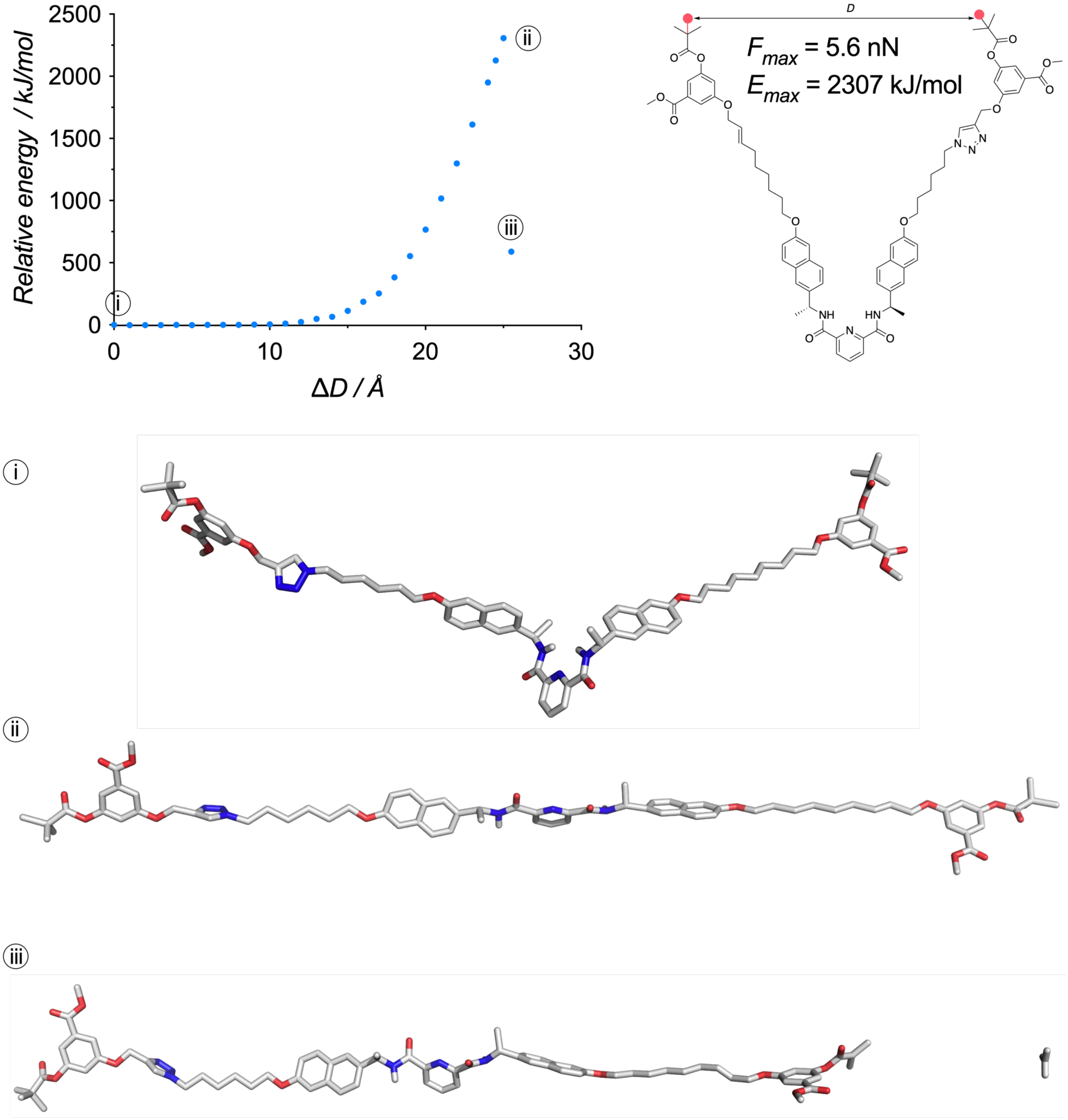

Figure 5. Evolution of energy of the ligand model 1 upon simulated elongation (CoGEF, DFT B3LYP/6-31G*) and structures at E_0 (i) E_{max} (ii), and scission (iii). F_{max} determined from the slope of the final 40% of the energy/elongation curve. Scissile bonds shown in red in the top structure.

9.4 Ligand model 2

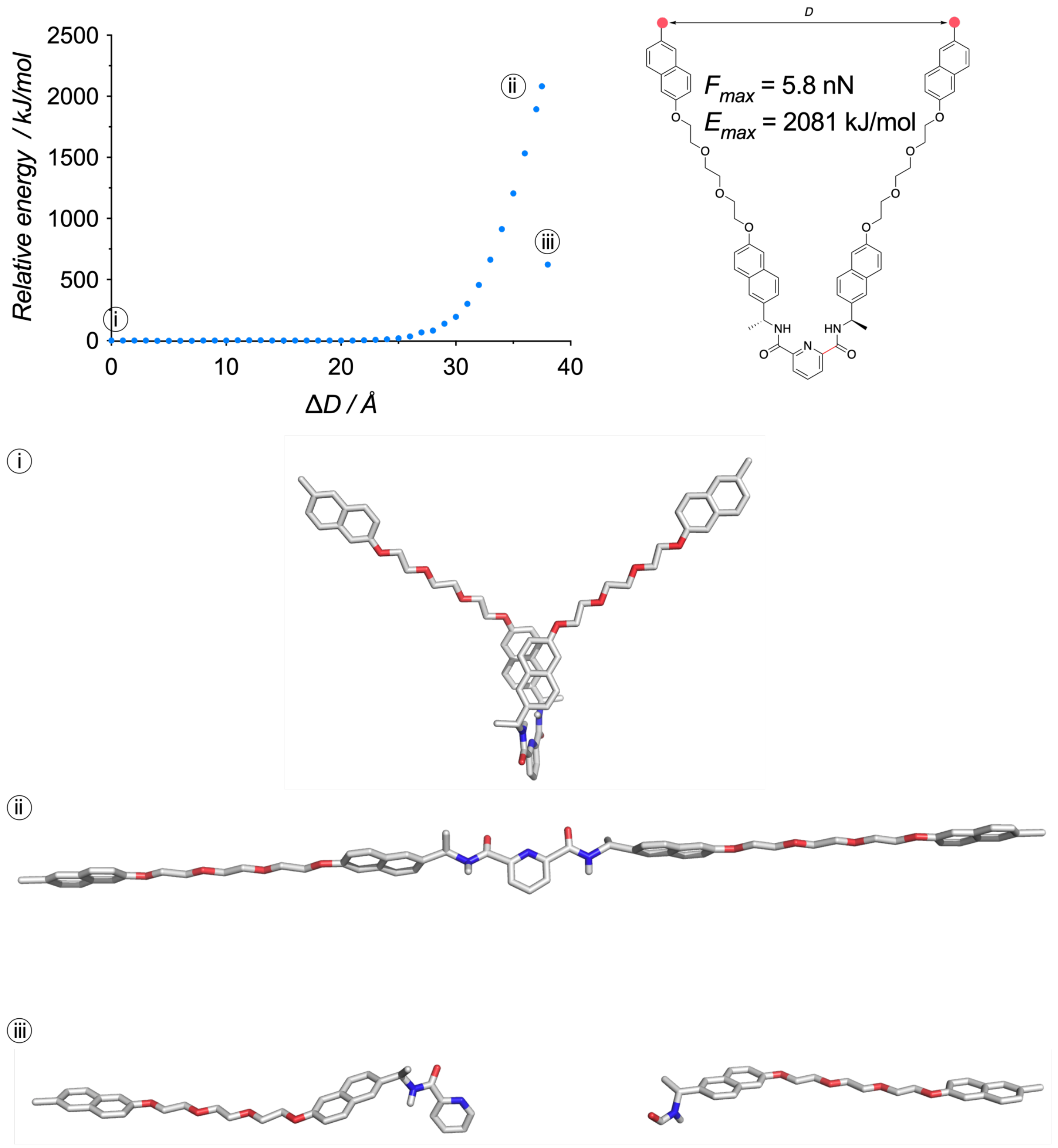

Figure 6. Evolution of energy of the ligand model 2 upon simulated elongation (CoGEF, DFT B3LYP/6-31G*) and structures at E_0 (i) E_{max} (ii), and scission (iii). F_{max} determined from the slope of the final 40% of the energy/elongation curve. Scissile bonds shown in red in the top structure.

9.5 Knot

9.5.1 MMFF

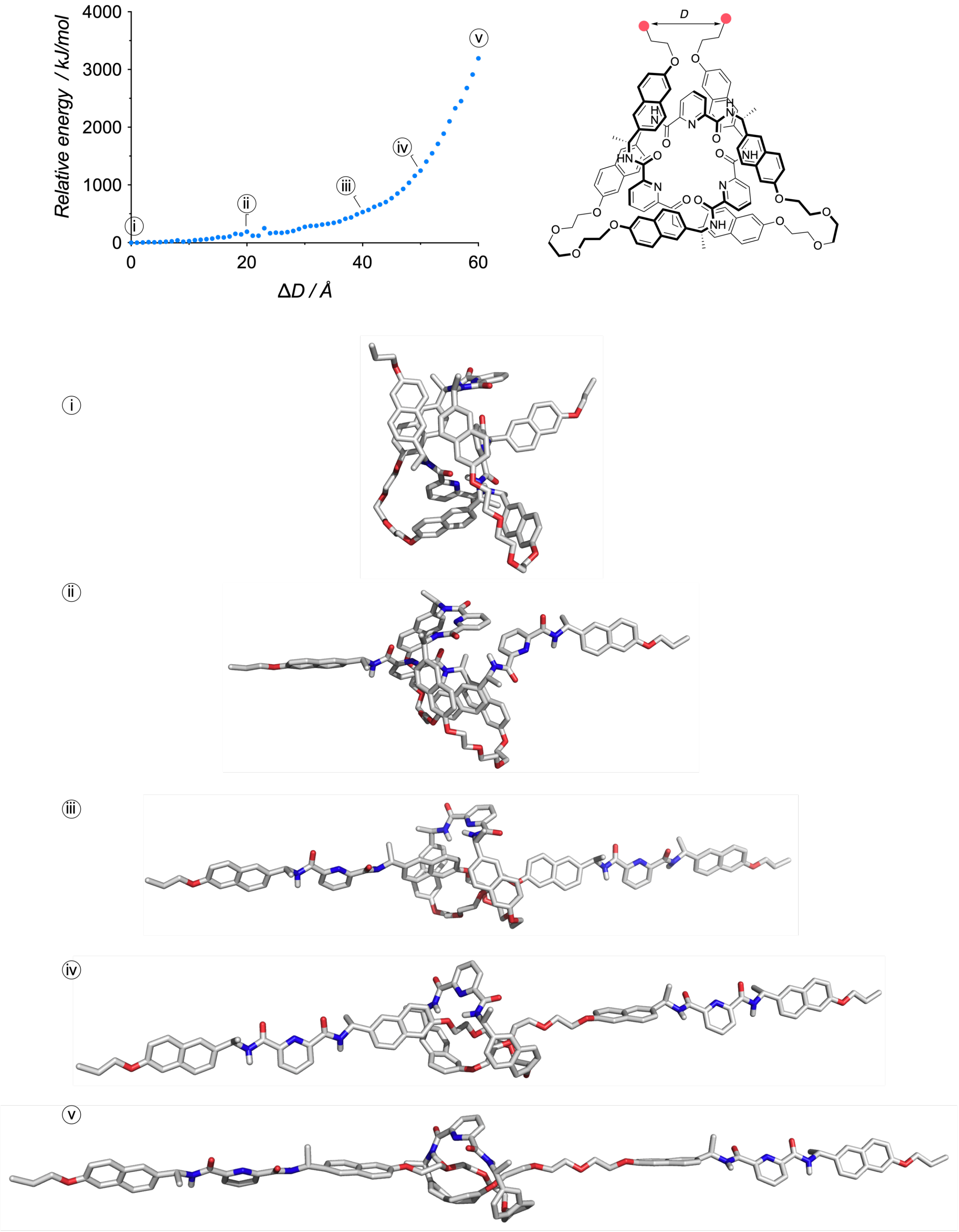

Figure 7. Evolution of energy of the knot model upon simulated elongation (CoGEF, MMFF) and structures at (i) E_0, (ii) 20 Å, (iii) 40 Å, (iv) 50 Å, and (v) 60 Å.

9.5.1 DFT (short model)

Unknotted sections were excised from structure (iii) of the MMFF profile to generate a shorter model (Supplementary Figure 8a). The elongation of this shorter model was then simulated by molecular mechanics (MMFF) then unrestricted DFT (UB3LYP/6-31G*) in vacuum until bond scission occurred. The distance between the anchor atoms (highlighted with a pink disk) ester was constrained and increased by increments of 0.5 Å then 0.1 Å for the final 0.5 Å. The relative energy of each intermediate was determined by setting the energy of the unknotted state (Supplementary Fig. 8b) at 0 kJ/mol. F_{max} values were determined from the slope of the final 40% of the energy/elongation curve (i.e. from E_{max} to 0.6 E_{max} or nearest point).

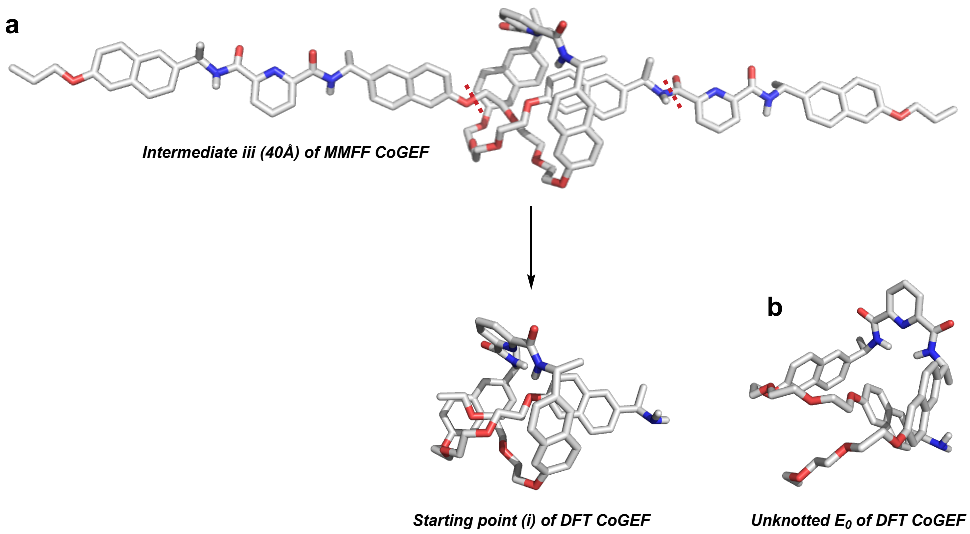

Figure 8. (a) A shorter model of the knot was generated by excluding the unknotted segments of intermediate (iii) from Supplementary Figure 7. (b) Unknotted model used as E_0 reference.

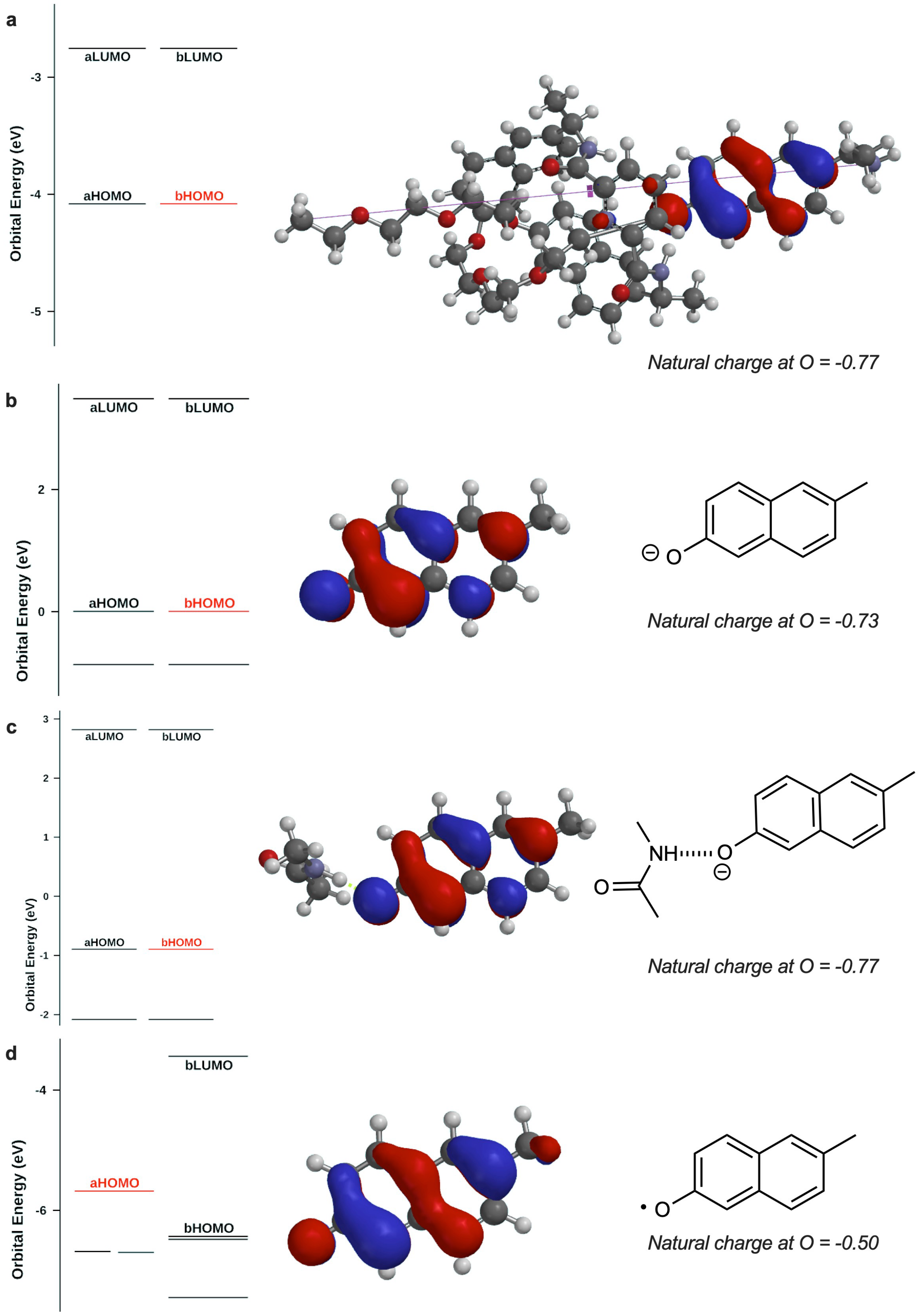

Figure 9. Natural charges at O and HOMO orbitals of (a) the broken short knot model (iii in Supplementary Figure 8), (b) a model anion, (c) a model anion hydrogen bonded with *N*-methylacetamide, and (c) a model radical, suggest a heterolytic scission (DFT UB3LYP/6-31G*, gas).

10 References

(1) Syrett, J. A., Mantovani, G., Barton, W. R. S., Price, D. & Haddleton, D. M. Self-healing polymers prepared via living radical polymerisation. *Polym. Chem.* **1**, 102–106 (2016.)
(2) Zhang, G., Gil-Ramírez, G., Markevicius, A., Browne, C., Vitorica-Yrezabal, I. J. & Leigh, D. A. Lanthanide template synthesis of trefoil knots of single handedness. *J. Am. Chem. Soc.* **137**, 10437–10442 (2015).
(3) Amir, F., Jia, Z. & Monteiro, M. J. Sequence control of macromers via iterative sequential and exponential growth. *J. Am. Chem. Soc.* **138**, 16600–16603 (2016).
(4) Hickenboth, C. R., Moore, J. S., White, S. R., Sottos, N. R., Baudry, J. & Wilson, S. R. Biasing reaction pathways with mechanical force *Nature* **446**, 423–427 (2007).
(5) Saitta, A., Soper, P., Wasserman, E. & Klein, M. Influence of a knot on the strength of a polymer strand. *Nature* **399**, 46–48 (1999).
(6) a) Bevington, J. C., Eaves, D. E. & Vale, R. L. Tests on the hydrolysis of certain synthetic polymers. *J. Poly. Sci.* **32**, 317–322 (1958). b) Talu, M. & Özgün, H. B. Alkaline hydrolysis of poly(ethyl acrylate) and styrene-ethyl acrylate copolymer. *Eur. Polym. J.* **26**, 5–7 (1990).
(7) Beyer, M. The mechanical strength of a covalent bond calculated by density functional theory. *J. Chem. Phys.* **112**, 7307–7312 (2000).
